# Diversity of Eukaryotic DNA Replication Origins Revealed by Genome-Wide Analysis of Chromatin Structure

**DOI:** 10.1371/journal.pgen.1001092

**Published:** 2010-09-02

**Authors:** Nicolas M. Berbenetz, Corey Nislow, Grant W. Brown

**Affiliations:** 1 Department of Molecular Genetics, University of Toronto, Toronto, Canada; 2 Donnelly Centre for Cellular and Biomolecular Research, University of Toronto, Toronto, Canada; 3 Department of Biochemistry, University of Toronto, Toronto, Canada; National Institute of Diabetes and Digestive and Kidney Diseases, United States of America

## Abstract

Eukaryotic DNA replication origins differ both in their efficiency and in the characteristic time during S phase when they become active. The biological basis for these differences remains unknown, but they could be a consequence of chromatin structure. The availability of genome-wide maps of nucleosome positions has led to an explosion of information about how nucleosomes are assembled at transcription start sites, but no similar maps exist for DNA replication origins. Here we combine high-resolution genome-wide nucleosome maps with comprehensive annotations of DNA replication origins to identify patterns of nucleosome occupancy at eukaryotic replication origins. On average, replication origins contain a nucleosome depleted region centered next to the ACS element, flanked on both sides by arrays of well-positioned nucleosomes. Our analysis identified DNA sequence properties that correlate with nucleosome occupancy at replication origins genome-wide and that are correlated with the nucleosome-depleted region. Clustering analysis of all annotated replication origins revealed a surprising diversity of nucleosome occupancy patterns. We provide evidence that the origin recognition complex, which binds to the origin, acts as a barrier element to position and phase nucleosomes on both sides of the origin. Finally, analysis of chromatin reconstituted *in vitro* reveals that origins are inherently nucleosome depleted. Together our data provide a comprehensive, genome-wide view of chromatin structure at replication origins and suggest a model of nucleosome positioning at replication origins in which the underlying sequence occludes nucleosomes to permit binding of the origin recognition complex, which then (likely in concert with nucleosome modifiers and remodelers) positions nucleosomes adjacent to the origin to promote replication origin function.

## Author Summary

Eukaryotic DNA replication begins at specific sites in the genome called replication origins, which are bound by the proteins that comprise the origin recognition complex (ORC). In budding yeast, there are more replication origins available than are used in any particular cell division cycle. Each origin has a characteristic time during the cell division cycle when the DNA replication machinery is assembled at a particular origin and begins to replicate DNA. Previous studies have indicated that differences in replication timing and origin use/availability may be a consequence of the chromatin structure surrounding an origin. Here we present a genome-wide analysis of nucleosome architecture of replication origins aligned by their ORC-binding site. We find that origins can be built with a variety of nucleosome occupancy patterns, and that these patterns are influenced by adjacent genomic features. Finally, we determined the genome-wide consequences of ORC depletion on nucleosome architecture at origins. ORC depletion allowed encroachment of flanking nucleosomes towards the origin and changed the nucleosome phasing, indicating that ORC acts as a barrier to position and phase nucleosomes. Our analysis provides a comprehensive, genome-wide view of replication origins that reveals a previously unappreciated diversity in origin structure.

## Introduction

All DNA transactions in living cells occur in the context of a highly regulated and dynamic chromatin structure. Not surprisingly, there is considerable evidence of functional relationships between nucleosomes, which are the basic repeating unit of chromosome structure, and origins of DNA replication. These relationships have been studied most extensively in the budding yeast *Saccharomyces cerevisiae*, largely due to the presence of well-defined replication origins in this organism, many of which have been identified on the basis of their ability to support plasmid maintenance *in vivo*. These sequences have been termed autonomously replicating sequences, or ARSs and many function as origins of replication in their chromosomal context. Budding yeast ARSs consist of an essential element, the ARS consensus sequence (ACS) as well as three elements that, while non-essential, contribute to origin function [1]. The ACS contains the binding site for the origin recognition complex (ORC), a six-member protein complex that is essential for the initiation of DNA replication [2]. A number of studies have sought to identify which ARSs function as *bona fide* replication origins in the chromosomal context *in vivo*. These include approaches in which the genomic location of newly-replicated DNA is identified using high resolution tiling microarrays [3], [4], and studies in which binding sites for ORC or other critical replication factors are mapped across the genome [5]–[7]. The most comprehensive annotation of functional replication origins currently available combines these datasets with phylogenetic analysis and functional analysis to define 228 functional ARSs, and to locate the ACS within each of these [8], [9].

Analysis of the canonical budding yeast replication origin *ARS1* shows that this origin is flanked by two positioned nucleosomes and that the ACS is located in a nucleosome-depleted region (NDR) [10]. Mutations in the origin which cause the ACS to become occupied by a nucleosome compromise origin function [11], presumably by occluding the ORC binding site. Mutations in the ORC binding site in both *ARS1* and *ARS307* allow nucleosomes to encroach upon the origin, indicating a role for ORC in maintaining a NDR at origins [12]. Interestingly, positioning nucleosomes away from the ORC binding site also compromise *ARS1* function without affecting ORC binding [12]. Together, these studies with single origins indicated that nucleosomes can have both a negative and a positive role in regulating origin function, and that ORC is important for positioning nucleosomes that flank the origin. The extent to which these properties are generalizable across all replication origins remains unclear. Here we ask if the predictive power of newly available genome-wide datasets can address the extensibility of these findings to each well-defined origin.

The availability of genome-wide maps of nucleosome positions in budding yeast has made it possible to investigate the relationship between replication origin function and nucleosome positioning on a global scale. The construction of these maps relies first on traditional nucleosome mapping tools whereby nucleosomes are cross-linked to DNA *in vivo*, followed by digestion with micrococcal nuclease (MNase) to degrade the linker DNA between nucleosomes. The mononucleosomal DNA, corresponding to the DNA contained within individual nucleosomes, is then hybridized to a high-resolution tiling microarray [13], [14] or sequenced either directly or after antibody immunoprecipitation [15]–[17] to identify the regions of the genome that are occupied by nucleosomes. Average views of such data across large numbers (82 to 248) of annotated replication origins [15]–[17] or views of several individual replication origins [8] largely confirm the single-origin view derived from studies of *ARS1*: replication origins tend to contain a nucleosome-depleted region (NDR) flanked by nucleosomes.

Here we use a comprehensively curated set of functional replication origins from budding yeast [8] combined with nucleosome maps constructed from tiling array hybridization of mononucleosome DNA [13] to analyze the chromatin structure at replication origins genome-wide. We find that the average view of chromatin organization at origins hides a surprising degree of diversity at individual origins. Since these origins are active in the chromosomal context, it suggests that functional origins can be built with a wide range of nucleosome positions relative to the ORC binding site. Genetic perturbation of ORC function caused origins to become more nucleosome-occupied and changed the phasing of the flanking nucleosomes. However, ORC-depleted origins did not become fully occupied by nucleosomes, likely because the underlying sequence at replication origins is resistant to nucleosome occupancy. Together these data provide a comprehensive view of the diversity of chromatin structure at replication origins, and suggest a model of nucleosome positioning at replication origins in which the underlying DNA sequence occludes nucleosomes to create a permissive environment for ORC binding, after which ORC positions nucleosomes in regular arrays on both sides of the ACS.

## Results

### Nucleosome organization at replication origins

Considerable insight into chromatin structure at promoter elements has been gleaned from recent analyses of whole-genome nucleosome maps in the budding yeast *S. cerevisiae*. These analyses are facilitated by the ability to align all of the promoters in the yeast genome centered on a single functional element, the transcription start site (TSS). Although some analysis of the nucleosome structure at DNA replication origins has been performed [8], [15]–[17], current views have not benefited from a systematic alignment of replication origins by a single functional element, analogous to the TSS for promoter analysis. Consequently, in the absence of such a fiduciary mark, these studies lack resolution. The most obvious feature with which to align replication origins is the ARS consensus sequence (ACS), a 15 bp motif present in all budding yeast origins characterized to date. Additionally, replication origins have an intrinsic asymmetry, with the B1 element positioned 3′ of the ACS when the ACS is oriented with the T-rich strand as the 5′ to 3′ strand. We used a comprehensively curated set of 228 ACSs [8], plus 50 ACSs annotated in the *Saccharomyces* Genome Database to generate ACS-aligned nucleosome maps for 222 ARSs. ARSs containing more than 9 duplicated microarray probes in the 800-bp region centered on the ACS where not included in our analysis. The nucleosome maps were aligned by the T residue at position 1 of the ACS and were oriented in the same direction. Although not all of these ACSs have been confirmed experimentally, those that have not are derived from the integration of three independent datasets: mapping of nascent replicating DNA [4], [18], genome-wide binding profiles of the essential initiation factors ORC and MCM complex [6], and evolutionary conservation among the *sensu stricto* yeast species [8], and so represent a high-quality dataset with extremely low levels of false-positive ACSs predicted.

**Figure 1:**
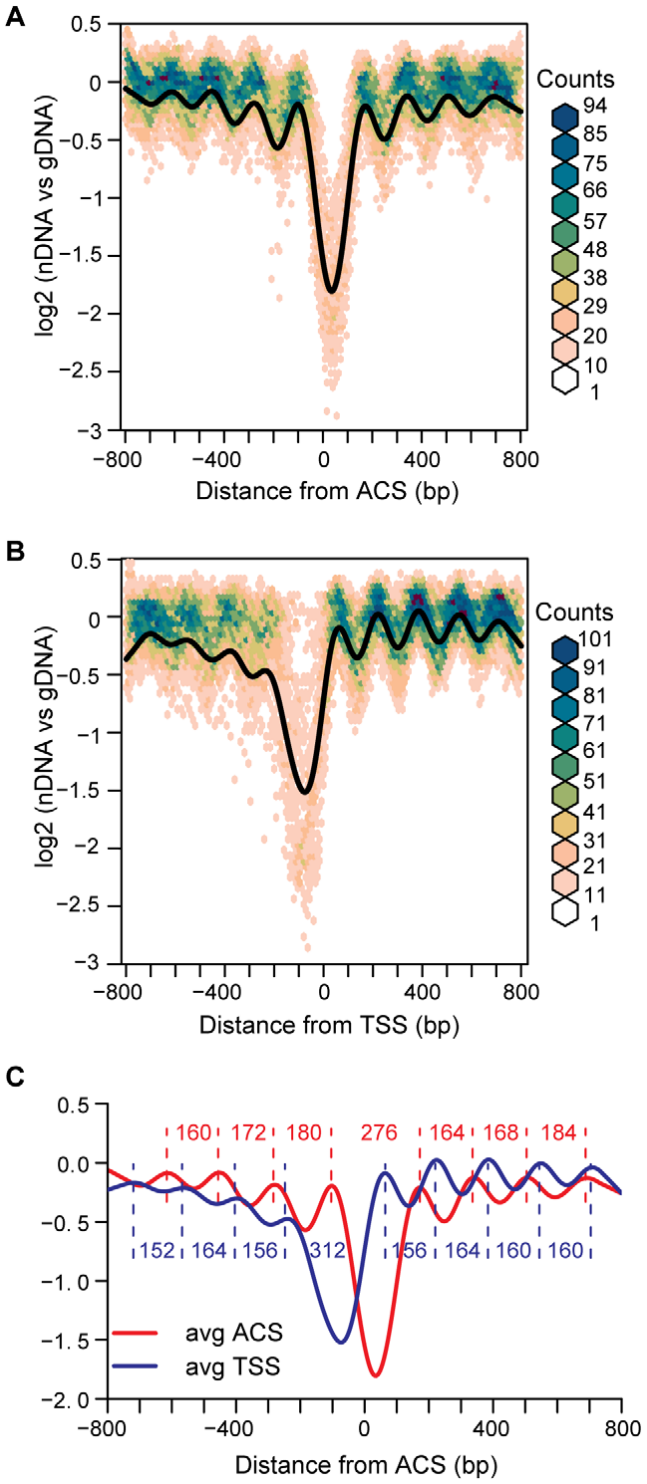
Average views of nucleosome occupancy at replication origins and transcription start sites. (A) Nucleosome maps at 222 replication origins were aligned by the ACS and oriented by the T-rich strand. The average is shown in black, overlaid on a bivariate histogram in which color indicates the density of the data at each point. (B) Nucleosome maps at 222 randomly-selected promoters, aligned by the transcription start site. (C) The average NDR widths for the ACS (N = 222) and the random subset of TSSs (N = 222). Distances in bp between nucleosome midpoints are indicated for the ACSs (red) and the TSSs (blue).

We first applied this alignment to high-resolution nucleosome maps derived from microarray analysis of nucleosomal DNA [13]. We compared this ACS-centered view of 222 replication origins to a TSS-centered view of 222 randomly selected promoters (Figure 1). As expected, the aggregate ACS-centered view, presented as an average plot in Figure 1A revealed a significant nucleosome depleted region (NDR) centered 36 bp to the right of the ACS. We measured the peak-to-peak distance between the nucleosomes flanking the NDR in the average plots (Figure 1C). When the ACS-centered average of the origins was compared to the TSS-centered view of 222 promoters (Figure 1B) several differences were apparent. The NDR for origins is, on average, narrower than that for promoters (∼276 bp vs ∼312 bp), and dramatically narrower than that previously reported for origins (500 bp) when origins were analyzed without the benefit of ACS alignment and without being oriented with respect to the T-rich strand [17]. The size of the average NDR that we measured contains ∼146 bp of DNA sequence that would be within the two flanking nucleosomes. Therefore ∼130 bp of DNA is free of nucleosomes at the average replication origin in budding yeast. This is significantly larger than the length of DNA that is protected by ORC [2], [19].

The ACS-centered average nucleosome map also reveals the presence of arrays of positioned nucleosomes extending away from the origin in both directions. The presence of a positioned nucleosome on each side of an NDR at replication origins has been previously noted [16], but the phased arrays of nucleosomes that are apparent in our analysis have not been described. By analogy with TSSs, we refer to the upstream flanking nucleosome as -1, and the downstream flanking nucleosome as +1. Although both origins and promoters are flanked by positioned nucleosomes, they differ in their spacing. The linker between the first two nucleosomes flanking ACSs (+1 and +2 or −1 and −2) is larger than that between the nucleosomes downstream of TSSs (Figure 1C). Additionally, the asymmetric organization of nucleosomes surrounding TSSs, with more discrete positioning of nucleosomes downstream of the TSS than upstream, is not apparent around ACSs, which have phasing that is equally discrete both upstream and downstream. This symmetrical arrangement of nucleosomes at origins might be functionally relevant, given that origins act in a symmetrical fashion in establishing bi-directional replication forks. A decay of the nucleosome phasing is apparent as one moves away from the ACS in both the “+” and “−” directions. This is similar to the decay of phasing seen at TSSs [13]–[15], [20] and, as proposed for TSSs [16], [21], suggests the nucleosomes upstream and downstream of the +1 and −1 flanking nucleosomes are statistically positioned. It is of interest that despite the propensity for replication origins to be located within intergenic regions [8], they do not adopt a nucleosome structure that is similar to that of the average promoter region. Finally, we plotted the data as bivariate histograms to display the diversity in the data that is not reflected in the average plot (Figure 1A and 1B). The considerable scatter in these plots suggests that there are substantial differences in nucleosome structure among the 222 ARSs analyzed.

### Nucleosome occupancy at replication origins correlates with DNA sequence and structural features

**Figure 2:**
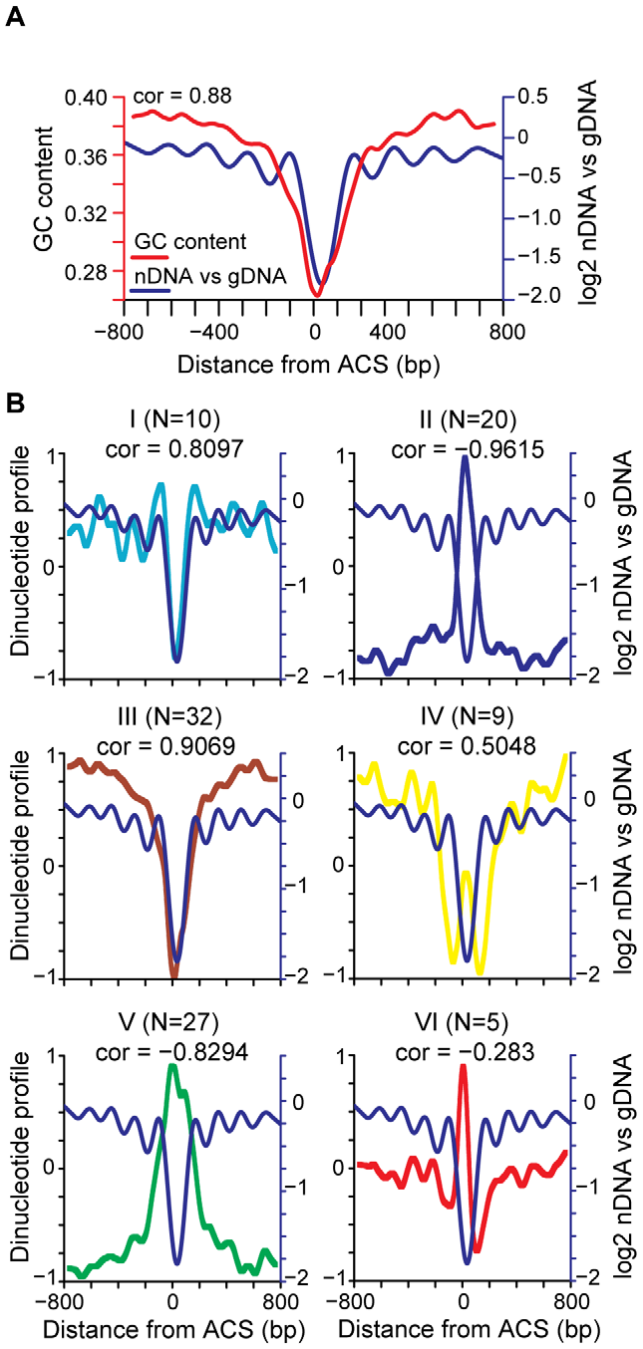
Correlation of nucleosome position with DNA sequence features. (A) GC content (red line) and nucleosome occupancy are plotted (blue line), with the Pearson correlation indicated. (B) Average dinucleotide profile is plotted for each of the six groups of dinucleotide properties (I–VI). The average nucleosome occupancy is also plotted for comparison (blue line). The average DNA dinucleotide profiles were partitioned into 6 groups using k-means clustering. The subcluster average DNA dinucleotide profile, re-scaled to a range of +1 to −1, is shown for each. The number of dinucleotide properties in each group (N) is indicated along with the Pearson correlation of each group with the average ACS profile.

Current models of nucleosome positioning suggest that nucleosome occupancy patterns are the combined result of contributions of DNA sequence, including periodic dinucleotide patterns and other structural and sequence features of DNA, and of protein factors, including chromatin remodeling factors and other DNA binding proteins [22]. For example, regions of high AT content are known to exclude nucleosomes [23] and poly (dA∶dT) tracts correlate with exclusion of nucleosomes at replication origins [17]. We sought to identify sequence features that correlate with the average nucleosome occupancy pattern at origins of replication. As expected we found that GC content was highly correlated with nucleosome depletion at the ACS, but described a much larger NDR and did not recapitulate the phasing adjacent to the ACS (Figure 2A). We compared 103 different dinucleotide properties [24] and found a number of dinucleotide properties that correlated with the average replication origin nucleosome occupancy pattern. The dinucleotide properties were grouped using k-means clustering (Figure S1) to show 6 general patterns. The average dinucleotide profile of each group is shown in Figure 2B, compared to the ACS-centered average nucleosome occupancy. Features in group I, which contains DNA structural features such as twist+rise and minor groove distance, describe the NDR width accurately, describe the positioned nucleosomes flanking the NDR, and to a lesser extent describe the positions of nucleosomes flanking the +1 and −1 nucleosomes. Dinucleotide features in group II anti-correlate with the NDR but do not describe the flanking nucleosomes. Group III and V features, such as melting temperature and free energy, tend to describe a more extensive NDR than observed in our average nucleosome map. Finally, groups IV and VI contain dinucleotide features that anti-correlate with the NDR, with the +1 and −1 nucleosomes, and to a lesser extent the flanking nucleosomes. We conclude that DNA sequence features contribute to nucleosome occupancy patterns at replication origins, particularly with respect to the NDR.

### A diversity of nucleosome occupancy patterns at replication origins

**Figure 3:**
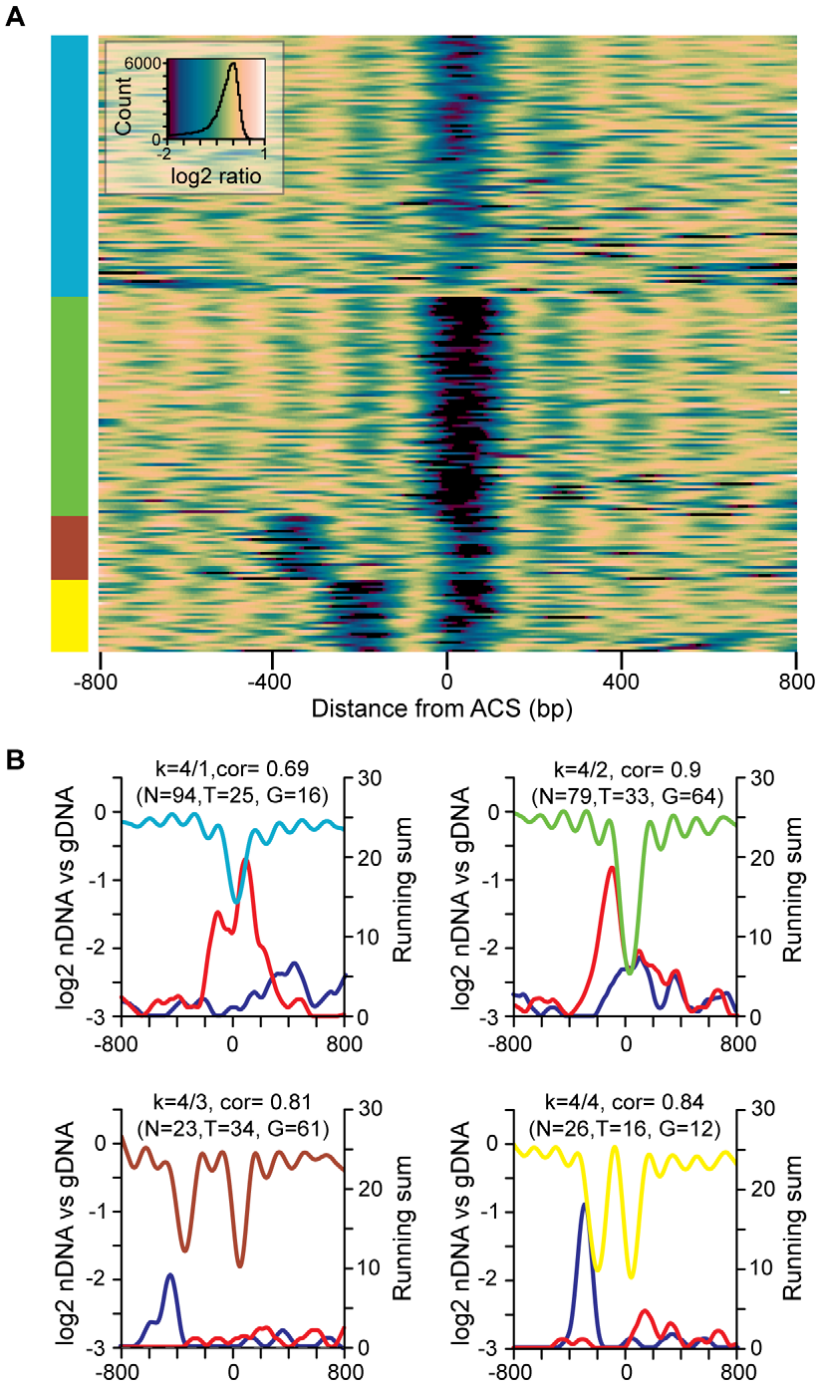
The diversity of nucleosome occupancy patterns at replication origins. (A) Heatmap of k-means clustered replication origins. ARSs are aligned on the Y axis, and the distance from the ACS is indicated on the X axis. Colors correspond to the log2 value of data points at a given position: in general, nucleosome occupancy is indicated in yellow and nucleosome depletion is indicated in blue/green. (B) Cluster averages for each of the four groups from the k-means clustering are plotted. Within each subcluster average plot the LOESS-smoothed moving sum of gene ends or TSSs located within 25 probe windows are plotted in blue (TSSs) or red (gene ends). The number of origins (N), TSS elements (T), and gene ends (G) in each group is indicated.

To categorize replication origins across the genome and to visualize the diversity suggested by the bivariate histograms, we used k-means clustering to group origins by the similarities of their nucleosome occupancy patterns surrounding the ACS. Analysis using 2 to 7 groups revealed several patterns of nucleosome occupancy surrounding the ACS (Figure S2). To highlight some of the diversity in individual origin profiles we produced a heatmap of origins assembled into 4 groups (Figure 3A). This grouping was chosen because it had low average inter-group correlations (all were below 0.7) indicating that the identified groups are relatively distinct. In the heat map, blue regions correspond to the NDR and linker regions while yellow regions are occupied by nucleosomes. Considerable diversity is apparent: the extent of the NDR varies as does the length of the linker 3′ to the flanking nucleosome, some origins have a second NDR either one or two nucleosomes 5′ of the major NDR, and some origins lack a clear NDR.

Although replication origins occur most often in intergenic regions, the different nucleosome patterns could represent the influence of other chromosomal features. We mapped the positions of nearby TSSs and translation stop sites (gene ends) for each of the nucleosome occupancy patterns (Figure 3B). For the two groups of origins (groups 3 and 4) that contain a second NDR to the left of the ACS there is a peak of TSS elements immediately 5′ to the second NDR. The NDR associated with TSSs is typically centered −50 to −100 bp relative to the TSS [13]–[17]. Therefore the position of the TSS elements 5′ to the second NDR of the origin profile indicates that the transcription units are oriented away from the ACS, as would be expected for origins positioned within intergenic regions. This orientation ensures that replication and transcription are co-directional. The NDR at the ACS for replication origins in groups 1 and 2 is associated with a peak in gene ends, again consistent with the intergenic location of most origins. Gene ends (i.e. 3′UTRs) are associated with low nucleosome occupancy [16], [25], which could contribute to the propensity of the region surrounding the ACS to remain unoccupied by nucleosomes. Together these data suggest that nucleosome occupancy at replication origins reflects proximity to TSSs and to gene ends. We did not detect a relationship between nucleosome occupancy and proximity to other prominent chromosomal features such as centromeres, telomeres, and adjacent ARSs.

**Figure 4:**
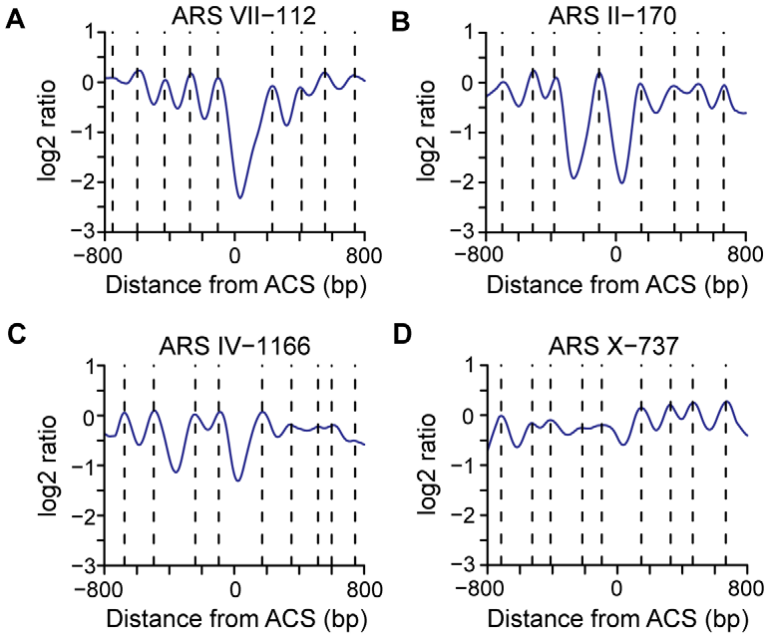
Representative nucleosome profiles and nucleosome calls for 4 origins. (A) A nucleosome profile (ARS VII-112) similar to the average ACS profile. (B) A nucleosome profile (ARS II-170) that contains a second NDR to the left of ACS-proximal NDR with a single nucleosome gap. (C) A nucleosome profile (ARS IV-1166) that contains a two nucleosome gap between two NDRs. (D) A nucleosome profile (ARS X-737) that lacks an NDR at the ACS. Dotted lines indicate the positions of nucleosome midpoints.

The diverse nature of nucleosome positions was also apparent when individual origins were analyzed (Figure 4 and Figure S3). In this analysis, the log2 ratios from the microarrays were used to determine the position of each individual nucleosome midpoint (indicated by broken lines, Figure 4, and red squares, Figure S3). Replication origins such as ARSVII–112 show a pattern similar to the average pattern. Origins such as ARSII–170 and ARSIV–1166 have a second NDR adjacent to the ACS. Some origins such as ARSX–737 lack a clear NDR. Since essentially all of the origins in this study are considered to be efficient, our data indicate that active, functional replication origins can be built with a variety of nucleosome occupancy patterns.

### Relationships among TSSs, gene ends, NDR width, and replication timing

**Figure 5:**
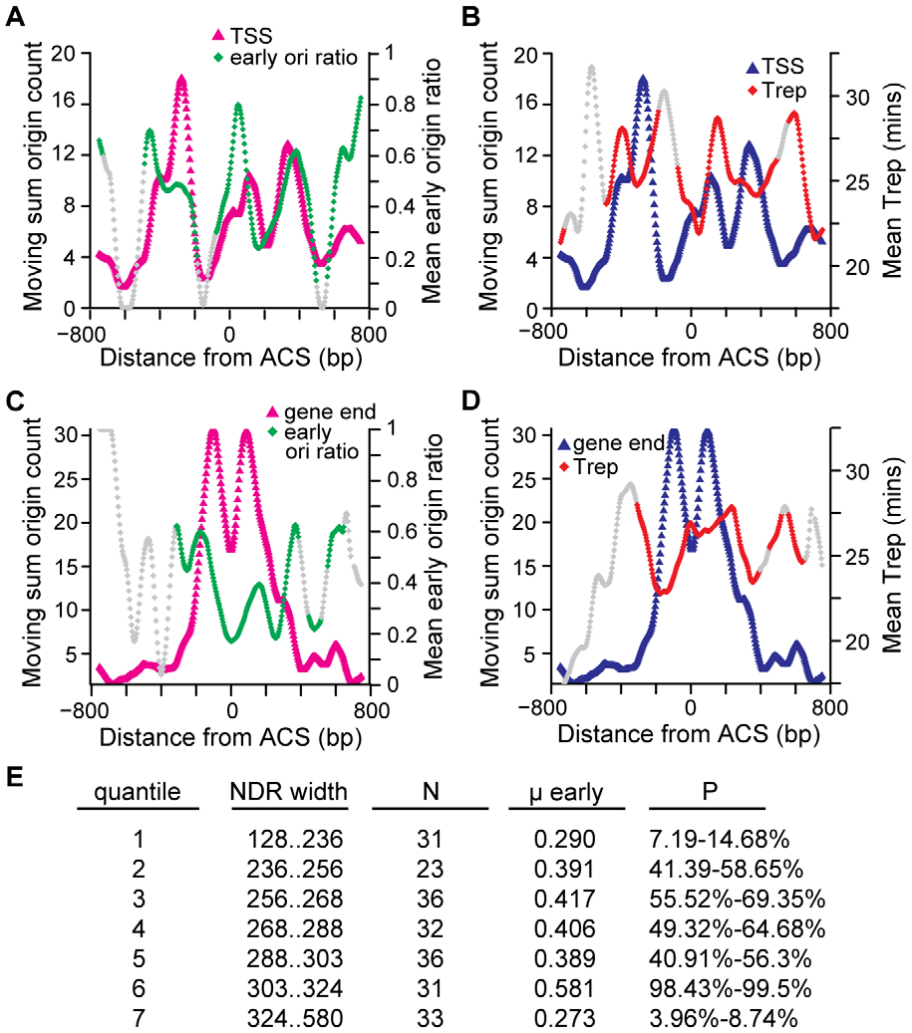
The relationship among replication timing and the locations of ACS-proximal TSSs and gene ends, and NDR widths. Distribution of 107 TSS locations (A,B) or 152 gene end locations (C,D) within 800bp of the ACS is plotted with early origin ratio (A,C) or replication time (B,D). TSS or gene end positions were counted within a moving window of 25 probes. Each position within the TSS or gene end distribution corresponds to the midpoint of the moving window and includes the total number of gene end or TSS locations counted within that window. The TSS or gene end distributions were LOESS-smoothed. Replication time was determined by identifying origins that contain a TSS or gene end within each 25 probe window and calculating the proportion of early origins [26] (mean early origin ratio, (A,C)) or replication time (mean Trep, panel B and D) [18]. Each position within the replication timing distribution corresponds to the midpoint of the 25 probe window and includes all of the origins which contained either a TSS or gene end within these probes. Windows with fewer than 5 origins are shaded grey so that effects from small numbers of origins are not considered. (E) NDR widths were divided into 7 quantiles and the proportion (μ early) of early origins [26] was compared to 10,000 similar sized samples of the original 222 origins in order to determine how many groups contained a less extreme proportion of early origins. P-values indicate the % of randomly re-sampled groups that had the same proportion of early origins. Extreme values, either close to 0 or close to 100, indicate the early origin proportion is at the low end (late firing) or the high end (early firing) of the distribution.

Our nucleosome mapping data indicated that there is a preferred arrangement of TSSs or gene ends with respect to a subset of origins. Analysis of individual origins (Figure S3) also suggested that there is variation in NDR width among replication origins. Accordingly, we asked whether there was any relationship between TSS and gene end locations or NDR width and the timing of replication origin firing, using genome-wide datasets that quantify origin timing *in vivo* [3], [4], [26]. Using the dataset of Feng et al. in which origin firing in the presence of HU (one definition of early origins) was determined by mapping the location of nascent ssDNA genome-wide [26] we found that TSS-proximal origins, those origins with a TSS within 800bp of the ACS, had a greater proportion of early origins, 0.47 (N = 107), than the entire ACS-containing origin data set, 0.39 (N = 222). This difference is significant as this proportion occurs relatively rarely, in the upper tail of the timing distribution (i.e., 98.3–99.1% of 10000 re-samples of 107 origins from the set of 222 origins have a lower proportion of early origins). For these 107 origins with a TSS within 800 bp of the ACS, we used a moving sum to describe the distribution of TSSs (Figure 5A, pink triangles). The TSS distribution was non-uniform, with peaks occurring at −276bp, +112bp, and +328bp relative to the ACS. We then determined the proportion of early origins [26] across the TSS distribution (Figure 5A, green diamonds). Local maxima in HU timing overlap the peaks in TSS distribution: origins with a TSS at these positions tend to fire early. A similar trend is apparent in the dataset of Raghuraman et al, which is derived from mapping of newly-replicated DNA using microarrays [3]. The TSS peaks overlap with the earliest replication times (local minima) in the timing dataset (Figure 5B). Interestingly, this trend was not evident in the final genome-wide replication timing dataset [4]. This may reflect the different methodologies used to determine replication timing. Indeed, the correlation among the different timing datasets is low. We conclude that the location of TSS elements relative to the ACS can influence replication timing. In particular, origins with a TSS ∼46 bp or ∼380 bp to the right of the ACS have a higher than average proportion of early origins and an earlier mean replication time.

A similar analysis was performed for gene ends (Figure 5C and 5D). As was the case for TSSs, the distribution of gene ends with respect to the ACS was non-uniform (Figure 5C, pink triangles). The clearest trend in our comparison to the Feng et al dataset [26] was that origins with a gene end positioned at the ACS tended to be late firing (a local minimum in the proportion of early origins; Figure 5C, green diamonds). This pattern was also observed when the Raghuraman et al dataset [3] was analyzed (Figure 5D). Thus, the location of gene ends relative to the ACS also influences replication timing, and in particular origins with a gene end at the ACS tend to fire late in the cell cycle.

Finally, we examined the distribution of NDR widths for the 222 origins. The NDR width distribution was divided into 7 quantiles (Figure 5E) and the proportion of early origins [26] was calculated for each. The quantile representing the narrowest NDRs (128 to 236 bp) had a low proportion of early origins (i.e., these origins tended to be late firing). Similarly, the origins with the widest NDRs (324 to 580 bp) also tended to be late firing. The earliest origins were found to have NDR widths between 303 and 324 bp. These data suggest that there is an NDR width that is optimal for early origin firing.

### Binding of the origin recognition complex positions nucleosomes at origins

**Figure 6:**
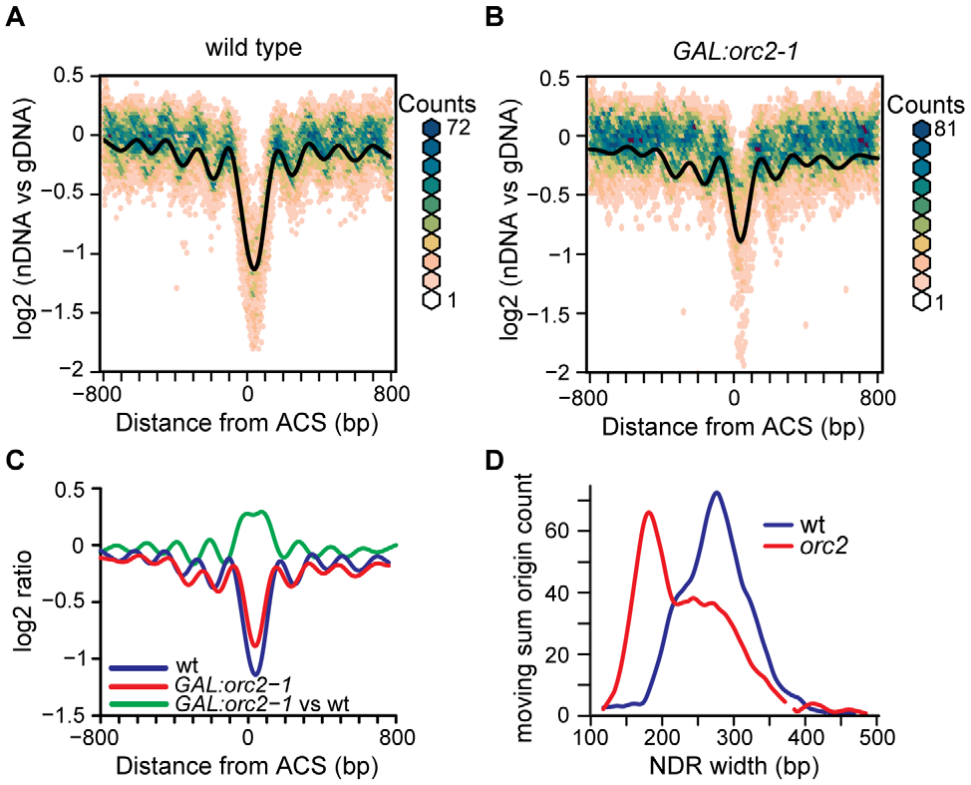
The effect of ORC depletion on nucleosome occupancy. (A) Nucleosome occupancy map of the wild-type control strain, plotted as in Figure 1. (B) Nucleosome occupancy map following ORC depletion in the *GAL:orc2-1* strain. (C) Average nucleosome occupancy plotted for wild-type (blue), *GAL:orc2-1* (red), and a difference plot comparing nucleosomal DNA from *GAL:orc2-1* to that from the wild-type strain (green). (D) NDR width distributions were calculated using a moving sum for windows containing 9 probes (36 bp) and LOESS-smoothed. The distribution in wild-type (blue line) and following Orc2 depletion (red line) is shown.

One reasonable candidate for a barrier element that establishes nucleosome positioning at replication origins is the binding of the origin recognition complex (ORC). To genetically perturb ORC function we took advantage of a *GAL1* promoter-driven *orc2-1* allele [27]. This allele produces Orc2 with a very short half-life [28] that is rapidly depleted when the *GAL1* promoter is repressed by the addition of glucose to the culture medium [27]. Depletion of Orc2 in mitosis greatly reduces ORC function, as the depleted cells accumulate in late G1 phase of the subsequent cell cycle, unable to initiate DNA replication [27] (Figure S4). We isolated nucleosomal DNA from Orc2-depleted and control cells, hybridized this DNA to tiling microarrays, and generated nucleosome occupancy maps (Figure 6A and 6B). As expected, the control nucleosome map is highly similar (correlation of 0.998) to that shown in Figure 1 and shows a similar NDR width distribution (Figure S5). By contrast, nucleosome positioning is altered when Orc2 is depleted (Figure 6B). To highlight the differences between WT and the Orc2 depletion strain we compared the nucleosome profile of the control cells to that of the Orc2-depleted cells across the 222 replication origins analyzed (Figure 6C, green line). The primary effect of Orc2 depletion was a shift of nucleosomes inward towards the ACS and an accompanying increase in nucleosome occupancy at the ACS. To quantify this change in NDR width, the microarray log2 ratios were used to determine the location of nucleosome midpoints. The nucleosome calls for each origin in the Orc2 depletion strain and wild-type (Figure S6) give an indication of nucleosome occupancy changes at each individual origin. Using these nucleosome calls we analyzed the influence of Orc2 depletion on the distance between the two nucleosomes flanking the ACS for each origin (Figure 6D) and determined that NDR width was reduced in a large fraction of origins. On average, the NDR was reduced from 276 bp in wild-type to 228 bp upon Orc2 depletion. The peak-to-trough height of the nucleosomes flanking the ARS was also slightly reduced, indicating that the nucleosomes became more delocalized upon Orc2 depletion. Together, these observations suggest that ORC contributes to the establishment of nucleosome positioning at replication origins. As a control we compared TSS-centered nucleosome maps of *GAL:orc2-1* and wild-type and found the maps to be almost identical (Figure S7). Although there was small decrease in nucleosome occupancy at TSSs (an effect opposite to that seen at ACSs), the positions of the nucleosomes flanking TSSs were unchanged, indicating that the effect of ORC depletion is specific to replication origins.

### The ACS remains nucleosome-free when chromatin is assembled *in vitro*

**Figure 7:**
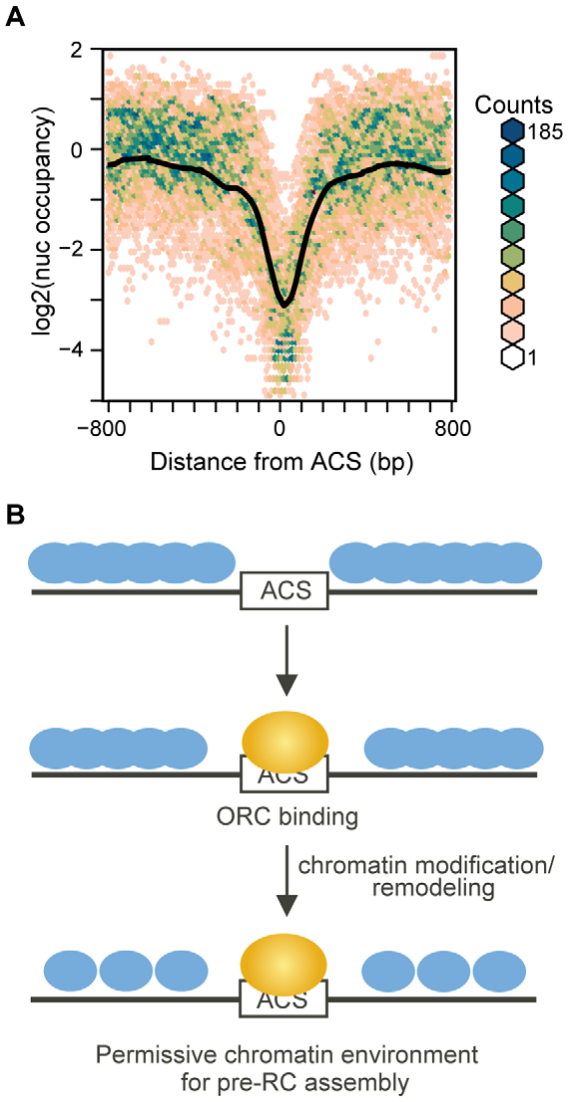
Nucleosome occupancy at replication origins in chromatin assembled *in vitro*. (A) Nucleosome maps at 174 replication origins in the *in vitro* nucleosome dataset [29] were aligned by the ACS and oriented by the T-rich strand. The average is shown in black, overlayed on a bivariate histogram in which color indicates the density of the data at each point. (B) Model of stepwise establishment of nucleosome positioning at replication origins. The DNA sequence surrounding the ACS specifies a low nucleosome occupancy, creating a permissive environment for ORC binding. Upon binding by ORC and recruitment of chromatin remodeling and modification activities the +1 and −1 nucleosomes are positioned. The adjacent nucleosomes then pack in uniformly-spaced arrays.

We noted that upon Orc2 depletion the ACS did not, on average, become completely nucleosome occupied. Although this could in part be due to incomplete inactivation of ORC, it is also possible that even in the complete absence of ORC the ACS would not become nucleosome-bound. This extreme case of complete ORC depletion is difficult to achieve *in vivo* because ORC genes are essential. We turned instead to analysis of maps of nucleosomes assembled on *S. cerevisiae* genomic DNA *in vitro* in the complete absence of non-histone proteins [29]. The average ACS-centered view of 174 ARSs in this dataset is shown in Figure 7A. When nucleosomes are assembled in the complete absence of ORC a large NDR remains at the ACS, indicating that the underlying sequence of the origin is a critical element that specifies the low nucleosome occupancy at the ACS, and offering an explanation for the persistent NDR we observed after ORC depletion. This is also consistent with our observation that a number of DNA sequence properties correlate with the low occupancy at the NDR (Figure 2B and Figure S1). It is worth noting, however, that the NDR in the *in vitro* map is substantially larger than those in the *in vivo* maps (445 bp vs 276 bp), similar to the case for promoters in this dataset. Interestingly, a number of dinucleotide sequence parameters also described NDRs larger than in the *in vivo* map (Figure 2B and Figure S1). One reasonable possibility is that the sequence surrounding the ACS occludes nucleosomes over a wider region than that observed in the *in vivo* maps, but the contributions of other proteins *in vivo* likely results in a denser nucleosome packing than is achieved in the *in vitro* reconstitutions [29], resulting in a greater encroachment of nucleosomes into the ACS region. Lastly, in the *in vitro* data, there was a complete absence of phasing of the nucleosomes adjacent to the ACS, indicating that while sequence plays a large role in preventing nucleosome formation at the ACS, the assembly of a phased array of positioned nucleosomes at replication origins likely requires the contribution of non-histone protein factors or higher histone density than was achieved *in vitro*.

## Discussion

We have produced a comprehensive nucleosome map of DNA replication origins in *S. cerevisiae*. Our analysis is distinct from previous genome-wide views of nucleosome position at replication origins [15]–[17] in that we combined a comprehensively curated set of origins in which the ACS element was accurately mapped [8] with the most comprehensive genome-wide nucleosome maps. In this manner, we detected the NDR flanked by nucleosomes that was evident in previous views (derived without critical alignment parameters [15]–[17]). But more importantly, we extend this view by detecting phased arrays of positioned nucleosomes extending from either side of the origin NDR.

Considerable diversity was evident in the replication origin nucleosome maps, reinforcing the notion that the average view does not reflect the different nucleosome occupancy patterns that exist at active, functional replication origins. We found that adjacent genomic features, most notably TSS elements and gene ends, can influence the nucleosome patterns at replication origins. In particular, the presence of an adjacent TSS can result in a second NDR in addition to the NDR at the ACS. We found that TSSs are distributed asymmetrically at replication origins and that maxima in the TSS distribution correlate with early origin firing. The presence of a second NDR could improve the accessibility of the replication origin for ORC or the proteins that are recruited by ORC, or factors bound at the promoter element within the second NDR could play a direct role in recruiting replication proteins to the pre-initiation complex. In either case the activity of the replication origin would be promoted, consistent with increased likelihood that these origins will be active in early S phase. We also found that extremes of NDR width, either narrow or wide, were characteristic of late origins. For example, origins with the narrowest NDR have higher than average occupancy at the ACS. This architecture could lead to a competition between nucleosomes and ORC for binding at the ACS, resulting in a reduced efficiency of origin firing, as previously suggested [11], [17], [30]. We conclude that functional replication origins can be built with different chromatin architectures, and that adjacent genomic features can influence the timing of replication origin firing.

Unfortunately, due to a lack of appropriate genome-wide datasets we were unable to test more sophisticated measures of origin robustness. Origin efficiency, or the likelihood that a given origin will fire in a given cell cycle, is an important parameter to test with respect to origin nucleosome architecture. This parameter is quite complex, however, encompassing not simply the intrinsic efficiency of an origin, but also the time during S phase when it fires (as later firing origins are more likely to be replicated passively from a neighboring origin), as well as the proximity of other origins, which also have unique efficiencies. As genome-wide origin efficiency datasets become available in *S. cerevisiae* our classification of different nucleosome patterns at replication origins will be an important tool for further investigating the impact of nucleosome structure on origin function. Accordingly, we expect the analysis presented here to represent a benchmark for future large-scale studies.

One attractive model of nucleosome positioning posits that uniformly-spaced arrays of nucleosomes, such as those seen downstream of TSSs, are the result of nucleosome packing adjacent to a barrier element [16], [20], [21], [31], [32]. This uniform spacing decays further away from the barrier element, and this decay is seen as a decrease in the peak to trough height. Our data suggests that, on average, replication origins conform to this statistical positioning model. The average ACS-centered view of replication origins revealed strongly positioned +1 and −1 nucleosomes flanked by arrays of phased nucleosomes in which the uniform spacing decays as one moves away from the ACS. As is the case with the +1 nucleosome at TSSs [21], the key to understanding nucleosome positioning at replication origins likely lies in understanding the elements responsible for positioning the +1 and −1 nucleosomes that flank the ACS. Analysis of the underlying sequence at replication origins gave conflicting results. On one hand, assembly of nucleosomes *in vitro* (in the complete absence of ORC) resulted in a larger NDR at the ACS than that observed *in vivo*, indicating that the intrinsic sequence preference of histones does not accurately describe the positions of the +1 and −1 nucleosomes. However, this large NDR is likely the result of lower nucleosome density (approximately 50% of the *in vivo* density) in the chromatin assembled *in vitro* [29], which might prevent the more dramatic encroachment of nucleosomes towards the ACS that is observed *in vivo*. Analysis of dinucleotide patterns revealed some sequence properties that predicted both an NDR of the expected size, and the positions of the +1 and −1 nucleosomes, indicating a role for sequence in positioning these critical nucleosomes. Perhaps the most compelling evidence that DNA sequence alone does not position the +1 and −1 nucleosomes at replication origins comes from genetic perturbation of the origin recognition complex. Upon depletion of ORC we found that most origins displayed a change in the position of the nucleosomes flanking the ACS, with nucleosomes shifting inwards towards the ACS. In addition, in many cases the flanking nucleosomes became delocalized. These changes result in a shift in the phasing of adjacent nucleosomes and in delocalization of adjacent nucleosomes. Thus, when ORC binding is compromised the position of the +1 and −1 nucleosomes is altered, consistent with ACS-bound ORC serving as a barrier element component. However, the nucleosome-free region that we observe *in vivo* when ORC is present is, at ∼130 bp, considerably larger than both the *in vitro* binding footprint of purified ORC [2] and the ORC footprint seen *in vivo* [33], [34], suggesting that bound ORC is not the sole barrier element. We propose that ORC, in concert with additional protein factors recruited by ORC, positions the nucleosomes that flank the NDR at origins of replication.

Together our data suggest a model of nucleosome assembly at replication origins (Figure 7B) in which the NDR is specified by the DNA sequence of the ARS. This NDR is narrower *in vivo* than *in vitro* due to the presence of chromatin remodeling and modifying activities, yet wider than the ORC binding site. This sequence-specified NDR creates a chromatin environment that is permissive for ORC binding to the ACS. Binding of ORC, and perhaps recruitment of chromatin remodelers and modifiers by ORC (such as Rpd3, Sir1, Hat1, and Hat2 [35]–[38]) specifies the position of the +1 and −1 nucleosomes, resulting in arrays of phased nucleosomes on either side of the ACS. These positioned nucleosomes then become important for the assembly of the pre-replicative complex of replication initiation proteins [12] prior to origin firing. One particularly attractive feature of this model is that it is consistent with the suspected role of chromatin structure in regulating replication origins in metazoans [39]–[42]. Perhaps in the more complex replication origins of higher eukaryotes the role of DNA sequence recognition by ORC has been partially replaced by a more direct interplay between nucleosomes and the origin recognition complex. That the bromo-adjacent homology domain, which interacts with nucleosomes in some contexts [43], facilitates the binding of human ORC with chromosomes [44] suggests a mechanism by which this could be achieved. It will of course be of tremendous interest to test whether nucleosome positioning at DNA replication origins is dictated by a combination of DNA sequence and ORC binding in other organisms, as appears to be the case in yeast.

Note added in proof: While this manuscript was under review a similar study was published [45]. Although the studies utilized different (but largely overlapping) origin/ACS lists and methodologies (sequencing vs. microarray hybridization), they reached complementary conclusions.

## Materials and Methods

### ACS-centered nucleosome maps

In this section, wild-type refers to the S288C nucleosomal dataset (http://chemogenomics.stanford.edu/supplements/03nuc/files/analyzed_data_complete_bw20.txt) [13]. The tiling array coordinates within this dataset refer to a February 2006 genome release. Nieduszynski et al., proACS coordinates for 228 origins refer to an October 2003 release [8]. To locate these ACSs within the February 2006 genome (http://hugheslab.ccbr.utoronto.ca/supplementary-data/tillo/nucleosomes/), the 15bp proACS for each origin was used to search the corresponding chromosomal sequence in order to find its location(s). In cases where more than one match was found (N = 8 origins), the closest ACS to the described ACS was chosen as the 2006 proACS. A coordinate was assigned to each ACS, as the minimum of its start/end proACS coordinates. Using SGD chromosomal features from February 2006, 65 ACSs were located. SGD proACS calls are 11bp long. To locate the 15bp proACS, the minimum of ACS start/end sites were subtracted by 2. These ACSs were annotated with their ORIdb identifier, and the entire list of Nieduszynski et al., and SGD ACSs was filtered for duplicate origin calls. This resulted in a list of 278 ACS calls (228 Nieduszynski + 50 SGD). This list was then filtered based on the criteria that at least 800bp of flanking sequence is located on either side of the ACS to give a list of 255 ACSs. The final list was obtained after origins which contained more than 9 duplicated probe sequences were removed. Duplicated sequences were identified from the tiling array BPMAP file using the R affy package to parse the BPMAP file. The coordinates and identities of origins are summarized in Table S1. The ACS coordinates can be used to extract nucleosome position information for individual origins from the web-accessible compendium of nucleosome positions at http://refnucl.atlas.bx.psu.edu [46].

ACS proximal probes, all probes within 800bp of the ACS were localized and converted to a text file where each position 0, represents the nearest ACS probe. When a probe is not located within a 4bp window, the value was assigned as NA. The orientation of the ACS, which strand is the T-rich strand, was taken into account by flipping the entire list of extracted (−)-sense, T-rich strand on the C strand, log2 values. This list (Table S2) was imported into R (R Development Core Team, Vienna, Austria; http://www.r-project.org/), and LOESS-smoothed using a span that encompassed 36 probes.

Using R, the mean-ACS centered ACS profile was generated and overlaid onto a bivariate histogram, generated using the R hexbin package. The hexbin serves as a two-dimensional error bar for each point within the mean ACS profile. As a comparison, a random subset of coding genes was obtained using a random number generator to pick 222 origins from a list of 5015 coding genes [13]. To calculate the average size of nucleosomes NDRs in ARSs and coding gene profiles, the locations of nucleosome midpoints, peak log2 values, were visually selected using R and the distance between points was determined.

### Analysis of dinucleotide sequence features

A list of 103 DNA dinucleotide properties were obtained from the DiProDB website [24]. The sequence of 222 oriented origins was used to count dinucleotides within 75bp windows using the count function of the Seqinr package [47]. At each window, the dinucleotide counts were multiplied by the corresponding property value, summed for all dinucleotides and divided by the total number of dinucleotides in the window. This value was then assigned to the central probe. In order to cluster the data the average DNA dinucleotide profile was rescaled, a linear conversion of a set of numbers so that the values lie in the range of -1 to 1, and LOESS-smoothed using a span of 76bp. Using the average and scaled DNA dinucleotide properties, the values between −372 to +424 around the ACS were clustered into 6 groups using the R-implementation of k-means clustering with 10000 iterations. The data were visualized using a heatmap in which each average DNA dinucleotide property is sorted by correlation with its k-means assigned subcluster average DNA dinucleotide property.

### A diversity of nucleosome occupancy patterns at replication origins

The ∼800-bp region (−372 to 424bp) which on average encompasses the region containing two nucleosomes surrounding the ACS was clustered using the R-implementation of k-means clustering with 10000 iterations. The heatmap was constructed using the heatmap.2 function of the R gplots package. Subclustered nucleosome occupancy patterns are based on the per-position average log2 value of origins within a particular cluster.

The genomic context of each origin in our dataset (N = 222) was determined by comparing the location of the ACS against a list of genomic features (Table S3): coding gene start/end sites (http://chemogenomics.stanford.edu/supplements/03nuc/files/clusters/polyA_segments_verified_coords.txt), telomeres and centromeres (http://downloads.yeastgenome.org/chromosomal_feature/archive/SGD_features.tab.200602.gz), and the locations of all ARSs (http://www.oridb.org) localized to the February 2006 genome release using BLAT (http://genome-test.cse.ucsc.edu/~kent/exe/). Genomic context was analyzed for each origin by determining the location of the closest centromere (CDEII element), telomeric region, origin region (ORIdb), gene start and gene end sites with respect to the ACS (Table S4). The orientation of genomic features was taken into account by determining the orientation of each genomic feature with respect to aligned origins (T-rich side of the ACS on the Watson strand). For each subcluster, the locations of gene ends and TSSs within 800bp of the ACS were determined using a moving sum count in which the number of TSSs or gene ends were counted within a 25 probe window. The moving sum distribution was LOESS-smoothed using a span encompassing 26 probes.

### Relationship among TSSs, gene ends, NDR width, and replication timing

Replication timing from Raghuraman et al. (N = 170) as well as origin activity in hydroxyurea from Yabuki et al (N = 222) and Feng et al. (N = 222) [3], [4], [26] were obtained from OriDB [9]. In order to compare the replication timing of all 222 origins, replication data (http://www.sciencemag.org/feature/data/raghu1064351/PooledHLData/pooledHLdata.html) was used to identify the nearest replication time data point closest to the ACS location. One caveat of this approach is the differences in genome builds between the ACS coordinates and the Raghuraman et al. data. The influence of genome build differences was not strong because replication timing data was smoothed in a 10-kb window: 159 of 170 origins were assigned replication times identical to those assigned by ORIdb and the remaining 11 origins differ by only ∼2.3 minutes.

The replication timing and origin activity in HU data was used to determine the average replication timing within 25 probe windows of TSSs or gene ends distributed within the 800bp region surrounding the ACS. The proportion of early origins and replication time was determined when a region of 25 probes contained more than 4 origins with either a TSS or a gene end. The early origin proportion distribution was LOESS-smoothed using a span which encompassed 26 probes.

NDR width was determined using microarray log2 ratios to determine the location of nucleosome midpoints. The nucleosome midpoint was defined in the 800bp region surrounding the ACS by determining the correlation of 26 probe windows against the 26 probes which encompassed the average log2 maxima on either side of the ACS. The local maxima which passed a correlation cutoff of 0.45 were defined as nucleosome midpoint locations. The ACS-proximal nucleosome calls on either side of the ACS were used to calculate the NDR width. The width distribution was determined using a moving sum with a window of 35bp. The proportion of early origins within each 35bp window was determined using the Feng et al. dataset. The NDR widths were divided into 7 quantiles in order to highlight changes in replication timing for different NDR widths. The proportion of early origins was found for each NDR width group and the P-value was determined by resampling 10,000 groups of identical size and determining how many samples contained early origin proportions that were less extreme.

### Nucleosome maps following ORC depletion

Nucleosomal DNA was obtained as described via micrococcal nuclease digestion [13] with the following modifications: increasing the size of cultures from 50mL to 200mL and modifications to nucleosomal DNA purification. Single colonies of either W303-1A or *GAL:orc2-1* [28] were inoculated into 25mL of YPAG and grown overnight (∼20h) at 30°C. The cultures were diluted to an OD ∼0.1/mL in a final volume of 200mL YPAG in a baffled 1L flask. Cultures were grown until an OD600 ∼0.6/mL and then blocked with nocodazole (Sigma) at a final concentration of 5µg/mL with a final concentration of 1% DMSO. Cells were blocked for 90 minutes, collected and resuspended in 200mL YPAD containing 5µg/mL nocodazole and 1% DMSO. Cells were blocked in YPAD for 60 minutes, collected and released into YPAD. Samples were collected every 15 minutes from 30 minutes to 2 hours after the release from the nocodazole, and analyzed by flow cytometry using a Guava EasyCyte (Massachusetts, US) following sample preparation as described [48].The final sample, at 2 hours post-release, was cross-linked using methanol-free formaldehyde at a final concentration of 2%. After the formaldehyde was quenched using 125 mM glycine for 5 minutes, the cells were collected, washed with 1× PBS, collected into a 50 mL Falcon tube, frozen using liquid N_2_ and stored at −80°C.

Following spheroplasting and micrococcal nuclease digestion, nucleosomal or genomic DNA was isolated using a phenol-extraction, followed by a phenol-chloroform extraction [13], followed by ethanol precipitation and resuspension in 50µL of dH_2_O and 4µL 10 mg/mL RNase A. RNA was digested for 3h at 37°C followed by ethanol precipitation and resuspension in 45µL H_2_O. The quality of DNA was assessed using 2% w/v agarose gels and an Agilent BioAnalyzer. DNA labeling and hybridization to 4bp resolution Affymetrix tiling arrays was as described [13].

Two biological replicates of *GAL:orc2-1* and W303-1A nucleosomal DNA microarrays were obtained along with one biological replicate of W303-1A genomic DNA (http://www.ebi.ac.uk/microarray-as/ae/, Accession Number: E-MEXP-2369). To get a view of nucleosome positioning within *GAL:orc2-1* or W303-1A the nucleosomal DNA CEL files were compared against the CEL file of W303-1A genomic DNA as described [13]. The text files from TAS were parsed in a similar manner as the Lee et al. wild-type data: the 1600bp window-centered on the ACS was extracted and oriented based on which strand contained the T-rich ACS sequence (Tables S5, S6, S7). To highlight differences between *GAL:orc2-1* and W303-1A origins, the text file obtained by comparing nucleosomal arrays *GAL:orc2-1* vs W303-1A was LOESS-smoothed and nucleosome locations were determined using the same criteria used to identify nucleosomes in the Lee et al. 2007 dataset.

### Nucleosome map for nucleosomes assembled *in vitro*

The normalized genome-wide locations of nucleosomes assembled onto naked yeast genomic DNA data file (http://genie.weizmann.ac.il/pubs/nucleosomes08/nucleosomes08_data.html) [29] was parsed to obtain the normalized log2 value of the 1600bp surrounding the ACS start coordinate. This dataset is missing values that are present in the tiling array data. Thus, origins which had at most 40 missing calls in the 800 bp region (N = 801 calls) surrounding the ACS (N = 174) were used to construct the average ACS profile of *in vitro* nucleosomes and bivariate histogram as for the wild type profile.

## Supporting Information

Figure S1 A heatmap of 103 dinucleotide sequence features arranged into 6 groups by k-means clustering.
(3.98 MB PDF)

Figure S2 Sub-cluster average nucleosome occupancy, TSS distribution, and gene end distribution for 222 origins grouped into k = 2 through k = 7 groups using k-means clustering.
(0.76 MB PDF)

Figure S3 ACS-centered nucleosome profiles for each origin in the wild type dataset.
(0.94 MB PDF)

Figure S4 Flow cytometric analysis of DNA content during Orc2 depletion.
(0.51 MB PDF)

Figure S5 NDR width distributions for wild type S288c and W303.
(0.18 MB PDF)

Figure S6 ACS-centered nucleosome profiles for each origin in GAL:orc2 and wild type control.
(1.53 MB PDF)

Figure S7 The effect of ORC depletion on nucleosome occupancy at TSS elements.
(0.23 MB PDF)

Table S1 ACS coordinates for all origins in the study.
(0.01 MB TXT)

Table S2 ACS-centered nucleosome signal and cluster membership for 222 origins from S288c
(0.75 MB TXT)

Table S3 Locations of telomeres, centromeres, ARSs, and coding genes used in the determination of the genomic neighbourhood surrounding origins.
(0.18 MB TXT)

Table S4 Relative locations of genomic features for origins (N = 278) identified from Nieduszynski et al. 2006 and the February 2006 SGD genome release.
(0.07 MB TXT)

Table S5 ACS-centered nucleosome signal and cluster membership for 222 origins from W303-1A
(0.76 MB TXT)

Table S6 ACS-centered nucleosome signal and cluster membership for 222 origins from *GAL:orc2-1*
(0.76 MB TXT)

Table S7 ACS-centered nucleosome signal and cluster membership for 222 origins from *GAL:orc2-1* versus W303-1A.
(0.75 MB TXT)
