# Supplementary material for: Diversity of Eukaryotic DNA Replication Origins Revealed by Genome-Wide Analysis of Chromatin Structure
Source: PLoS Genet. 2010 Sep 2;6(9):e1001092. doi: 10.1371/journal.pgen.1001092 (PMC2932696; doi:10.1371/journal.pgen.1001092)
Supplement: Figure S1 — A heatmap of 103 dinucleotide sequence features arranged into 6 groups by k-means clustering. (3.98 MB PDF) [file pgen.1001092.s001.pdf]

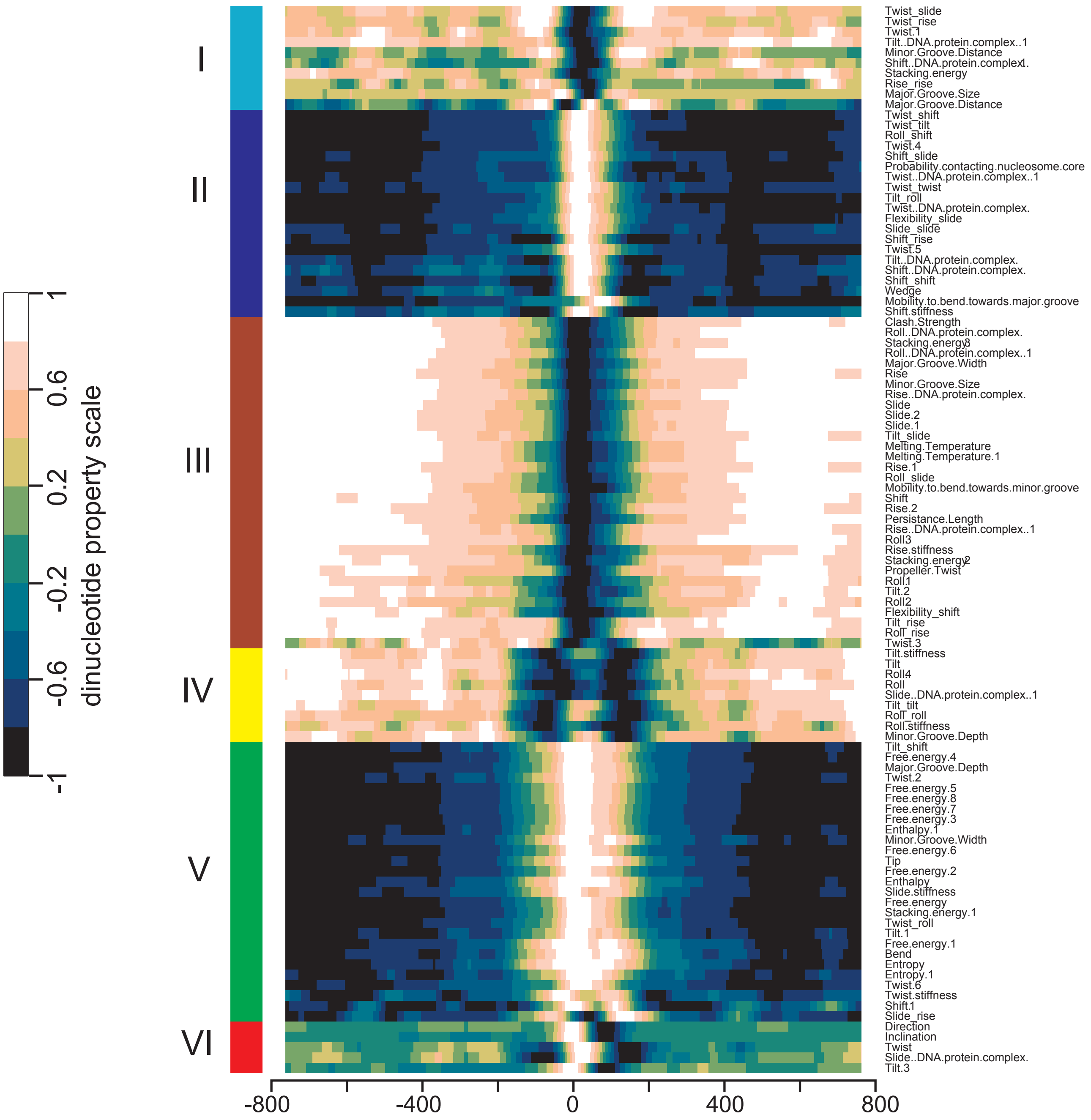

Figure S1: Heatmap of 103 DNA dinucleotide properties clustered into 6 groups using k-means clustering. Each average DNA dinucleotide profile was rescaled into the range -1 to 1, LOESS smoothed, and then clustered using k-means clustering (k=6) following 10000 iterations.
