# Supplementary material for: Diversity of Eukaryotic DNA Replication Origins Revealed by Genome-Wide Analysis of Chromatin Structure
Source: PLoS Genet. 2010 Sep 2;6(9):e1001092. doi: 10.1371/journal.pgen.1001092 (PMC2932696; doi:10.1371/journal.pgen.1001092)
Supplement: Figure S2 — Sub-cluster average nucleosome occupancy, TSS distribution, and gene end distribution for 222 origins grouped into k = 2 through k = 7 groups using k-means clustering. (0.76 MB PDF) [file pgen.1001092.s002.pdf]

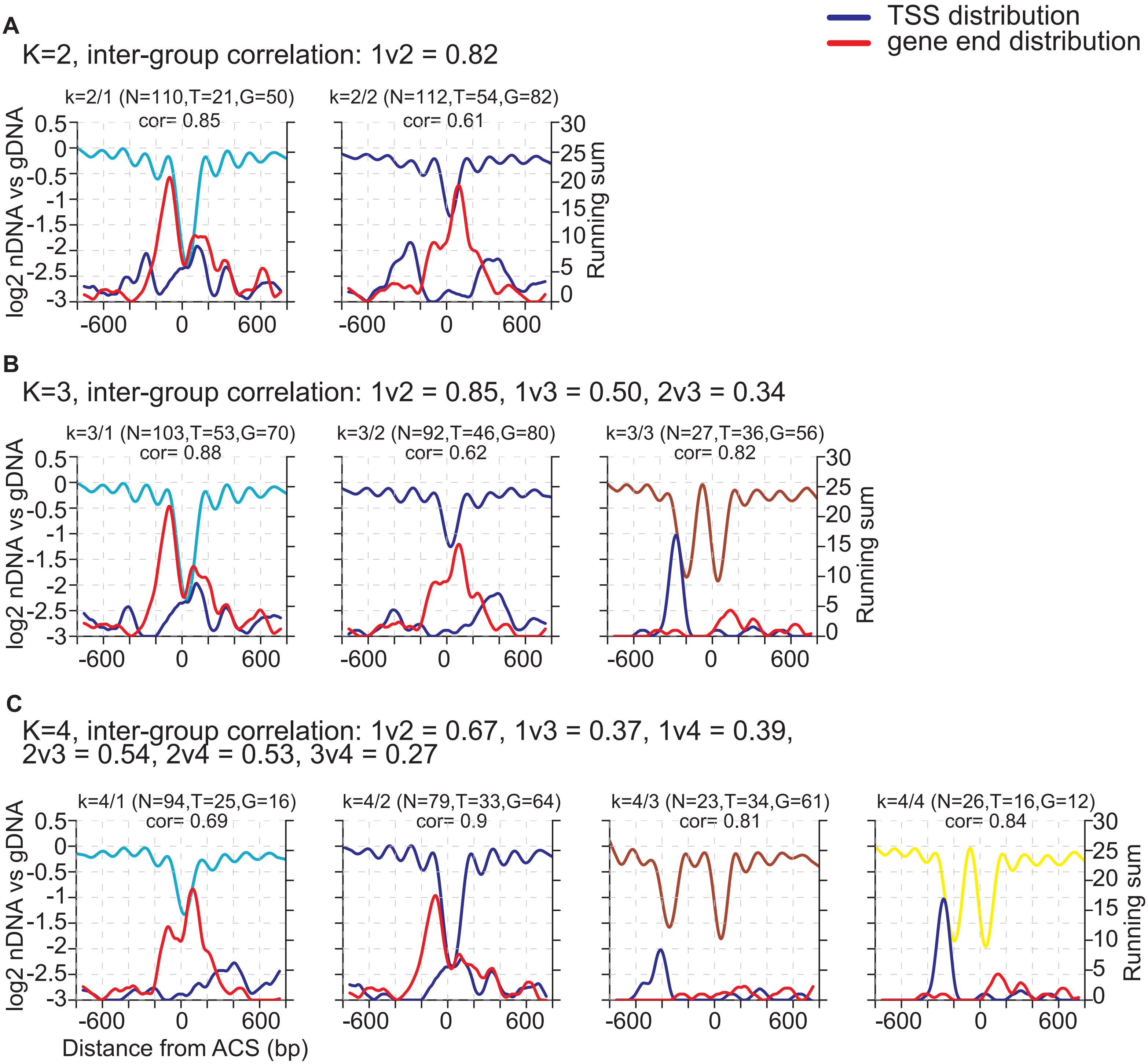

TSS distribution
gene end distribution
A
K=2, inter-group correlation: 1v2 = 0.82
k=2/1 (N=110,T=21,G=50)
cor= 0.85
k=2/2 (N=112,T=54,G=82)
cor= 0.61
B
K=3, inter-group correlation: 1v2 = 0.85, 1v3 = 0.50, 2v3 = 0.34
k=3/1 (N=103,T=53,G=70)
cor= 0.88
k=3/2 (N=92,T=46,G=80)
cor= 0.62
k=3/3 (N=27,T=36,G=56)
cor= 0.82
C
K=4, inter-group correlation: 1v2 = 0.67, 1v3 = 0.37, 1v4 = 0.39,
2v3 = 0.54, 2v4 = 0.53, 3v4 = 0.27
k=4/1 (N=94,T=25,G=16)
cor= 0.69
k=4/2 (N=79,T=33,G=64)
cor= 0.9
k=4/3 (N=23,T=34,G=61)
cor= 0.81
k=4/4 (N=26,T=16,G=12)
cor= 0.84
log2 nDNA vs gDNA
Running sum
Distance from ACS (bp)
0.5
0
-0.5
-1
-1.5
-2
-2.5
-3
30
25
20
15
10
5
0
-600
0
600

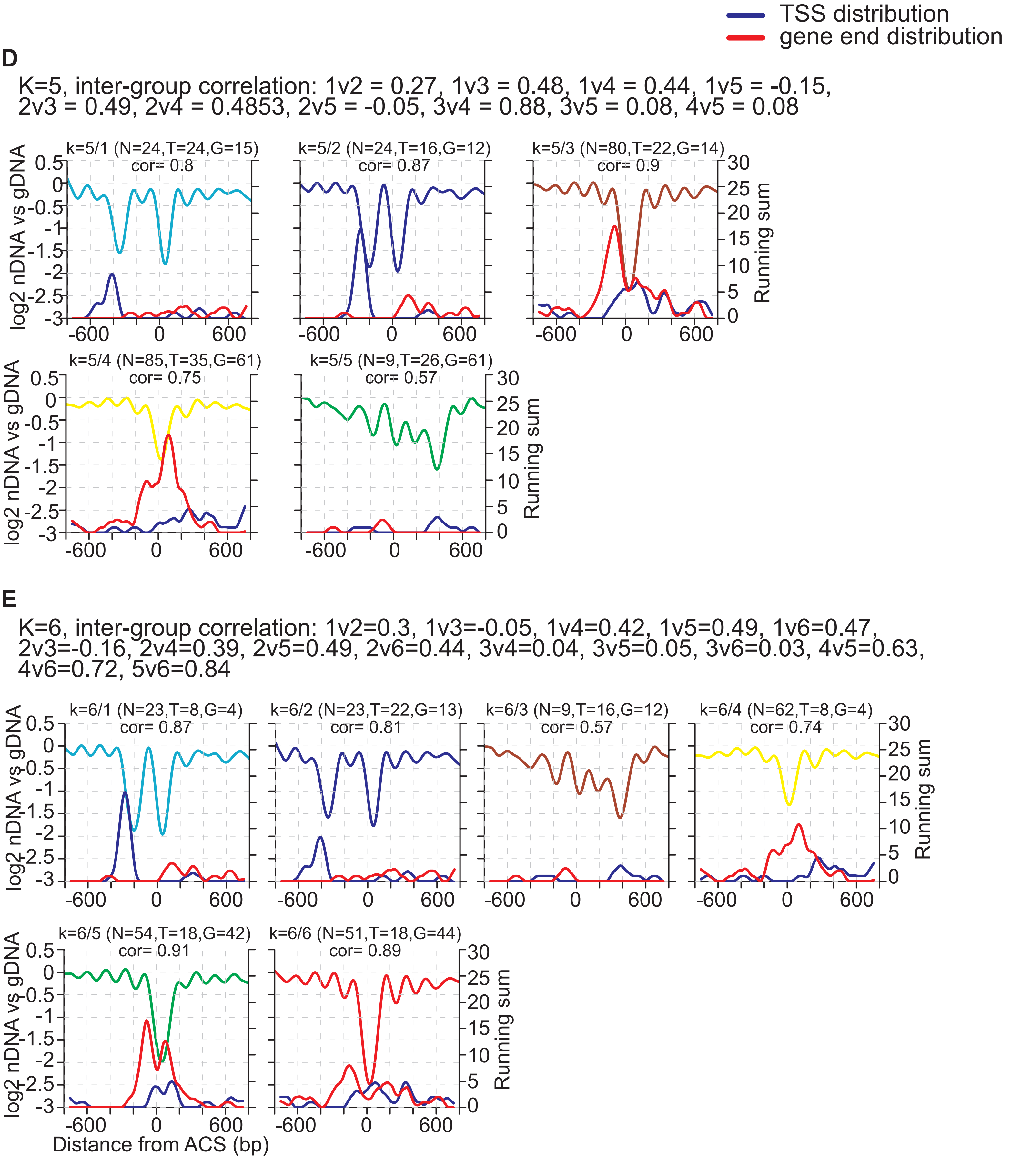

TSS distribution
gene end distribution
D
K=5, inter-group correlation: 1v2 = 0.27, 1v3 = 0.48, 1v4 = 0.44, 1v5 = -0.15, 2v3 = 0.49, 2v4 = 0.4853, 2v5 = -0.05, 3v4 = 0.88, 3v5 = 0.08, 4v5 = 0.08
k=5/1 (N=24,T=24,G=15) cor= 0.8
k=5/2 (N=24,T=16,G=12) cor= 0.87
k=5/3 (N=80,T=22,G=14) cor= 0.9
k=5/4 (N=85,T=35,G=61) cor= 0.75
k=5/5 (N=9,T=26,G=61) cor= 0.57
log2 nDNA vs gDNA
Running sum
E
K=6, inter-group correlation: 1v2=0.3, 1v3=-0.05, 1v4=0.42, 1v5=0.49, 1v6=0.47, 2v3=-0.16, 2v4=0.39, 2v5=0.49, 2v6=0.44, 3v4=0.04, 3v5=0.05, 3v6=0.03, 4v5=0.63, 4v6=0.72, 5v6=0.84
k=6/1 (N=23,T=8,G=4) cor= 0.87
k=6/2 (N=23,T=22,G=13) cor= 0.81
k=6/3 (N=9,T=16,G=12) cor= 0.57
k=6/4 (N=62,T=8,G=4) cor= 0.74
k=6/5 (N=54,T=18,G=42) cor= 0.91
k=6/6 (N=51,T=18,G=44) cor= 0.89
log2 nDNA vs gDNA
Running sum
Distance from ACS (bp)

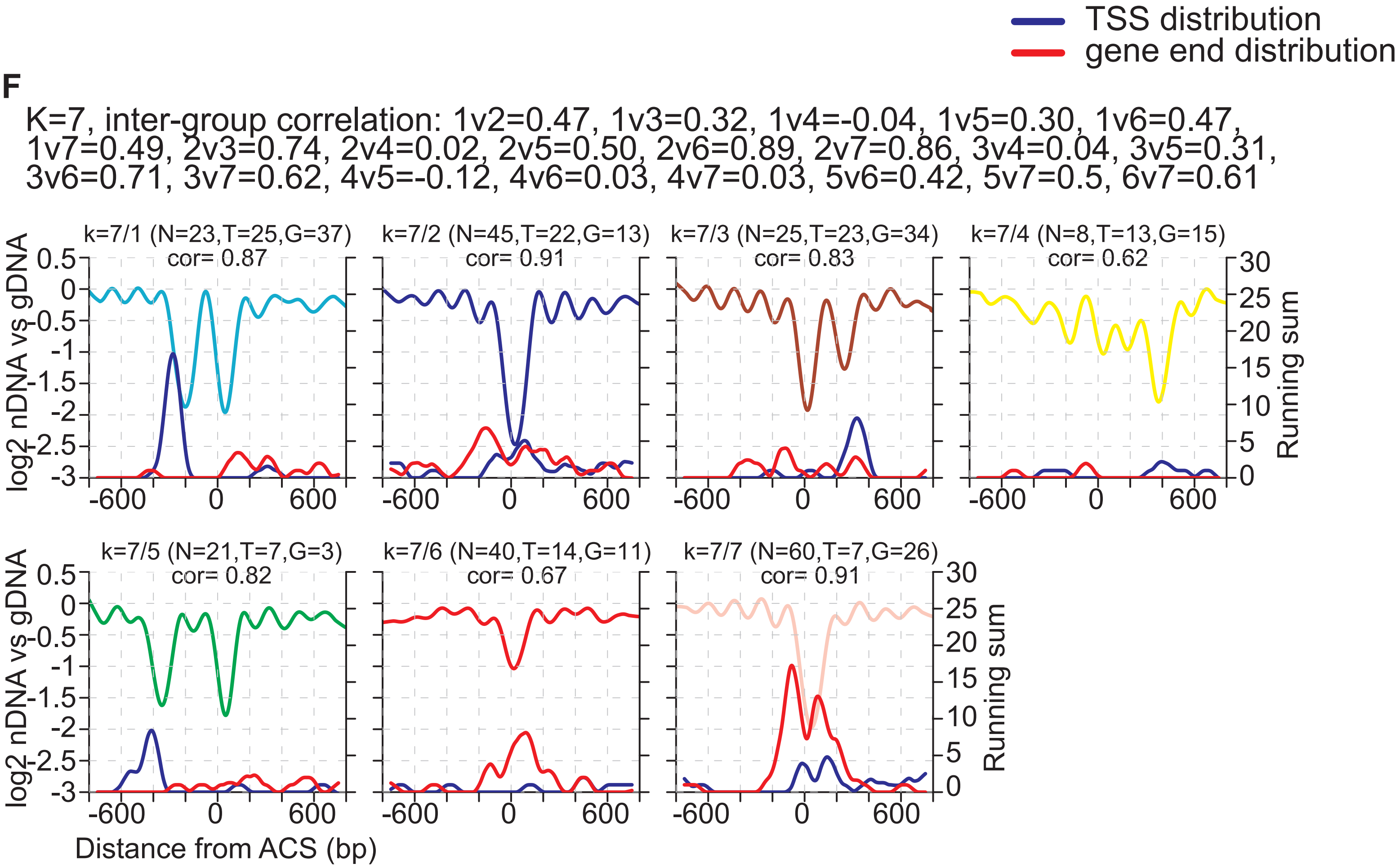

Figure S2. Subcluster average, TSS (—) and gene end (—) distribution obtained following kmeans clustering of the wild-type (k=2 to k=7) following 10,000 iterations. For each grouping the number of origins in each subcluster is indicated (N), along with the number of TSSs (T), gene ends (G), and the correlation of each subcluster against the average ACS profile (cor).
