# Supplementary material for: Diversity of Eukaryotic DNA Replication Origins Revealed by Genome-Wide Analysis of Chromatin Structure
Source: PLoS Genet. 2010 Sep 2;6(9):e1001092. doi: 10.1371/journal.pgen.1001092 (PMC2932696; doi:10.1371/journal.pgen.1001092)
Supplement: Figure S3 — ACS-centered nucleosome profiles for each origin in the wild type dataset. (0.94 MB PDF) [file pgen.1001092.s003.pdf]

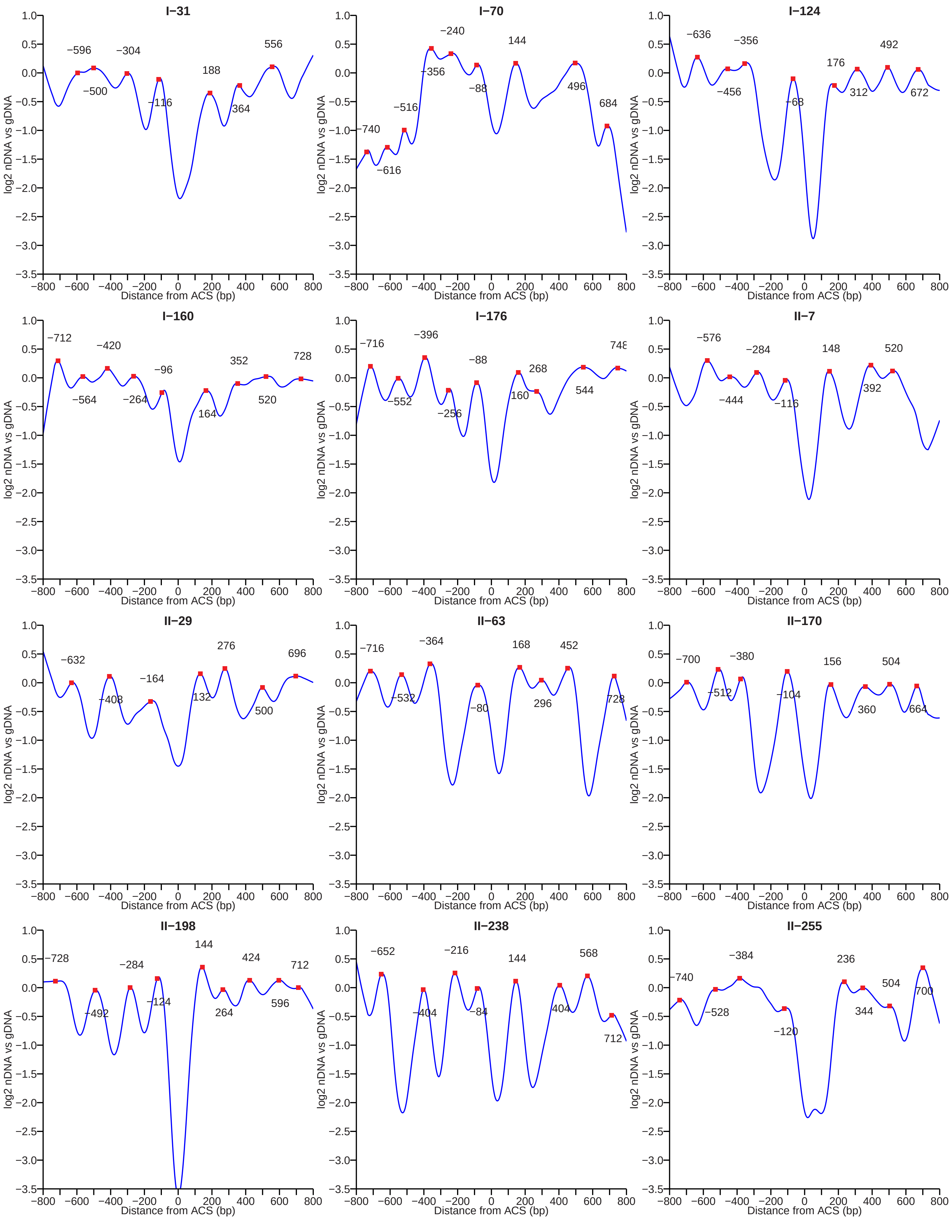
I−31
−596
−304
556
−500
188
−116
364
I−70
−240
144
−356
−516
−88
496
684
−740
−616
I−124
−636
−356
492
176
−456
312
672
−68
I−160
−712
−420
728
−564
−264
−96
352
520
164
I−176
−716
−396
748
−88
268
−552
−256
160
544
II−7
−576
−284
148
520
−444
−116
392
II−29
−632
−164
276
696
−408
132
500
II−63
−716
−364
168
452
−532
−80
296
728
II−170
−700
−380
156
504
−512
−104
360
664
II−198
−728
−284
144
424
712
−492
−124
264
596
II−238
−652
−216
144
568
−404
−84
404
712
II−255
−740
−384
236
504
−528
−120
344
700
log2 nDNA vs gDNA
Distance from ACS (bp)

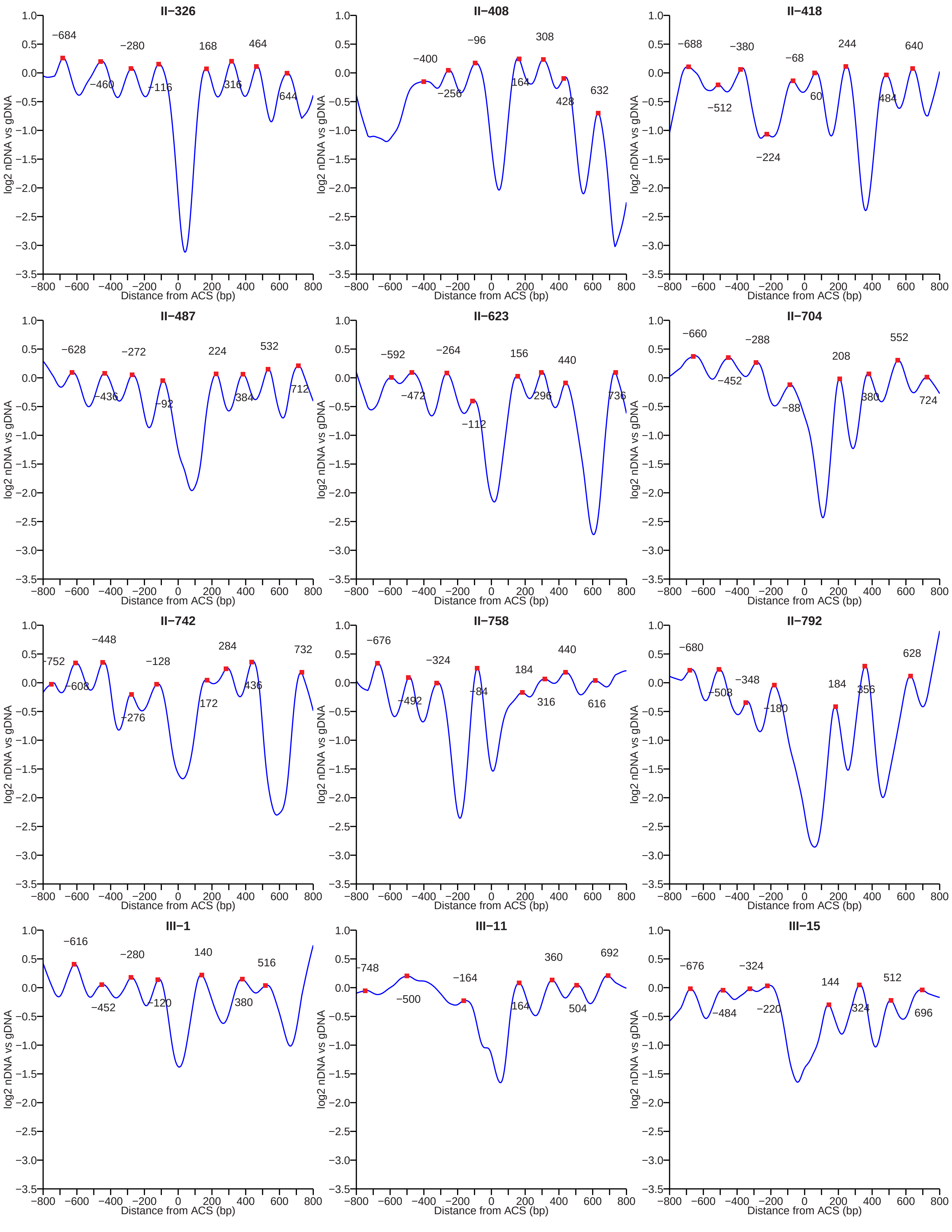
II−326
−684
−460
−280
−116
168
316
464
644
II−408
−400
−256
−96
164
308
428
632
II−418
−688
−512
−380
−224
−68
60
244
484
640
II−487
−628
−436
−272
−92
224
384
532
712
II−623
−592
−472
−264
−112
156
296
440
736
II−704
−660
−452
−288
−88
208
380
552
724
II−742
−752
−608
−448
−276
−128
172
284
436
732
II−758
−676
−492
−324
−84
184
316
440
616
II−792
−680
−508
−348
−180
184
356
628
III−1
−616
−452
−280
−120
140
380
516
III−11
−748
−500
−164
164
360
504
692
III−15
−676
−484
−324
−220
144
324
512
696
log2 nDNA vs gDNA
Distance from ACS (bp)

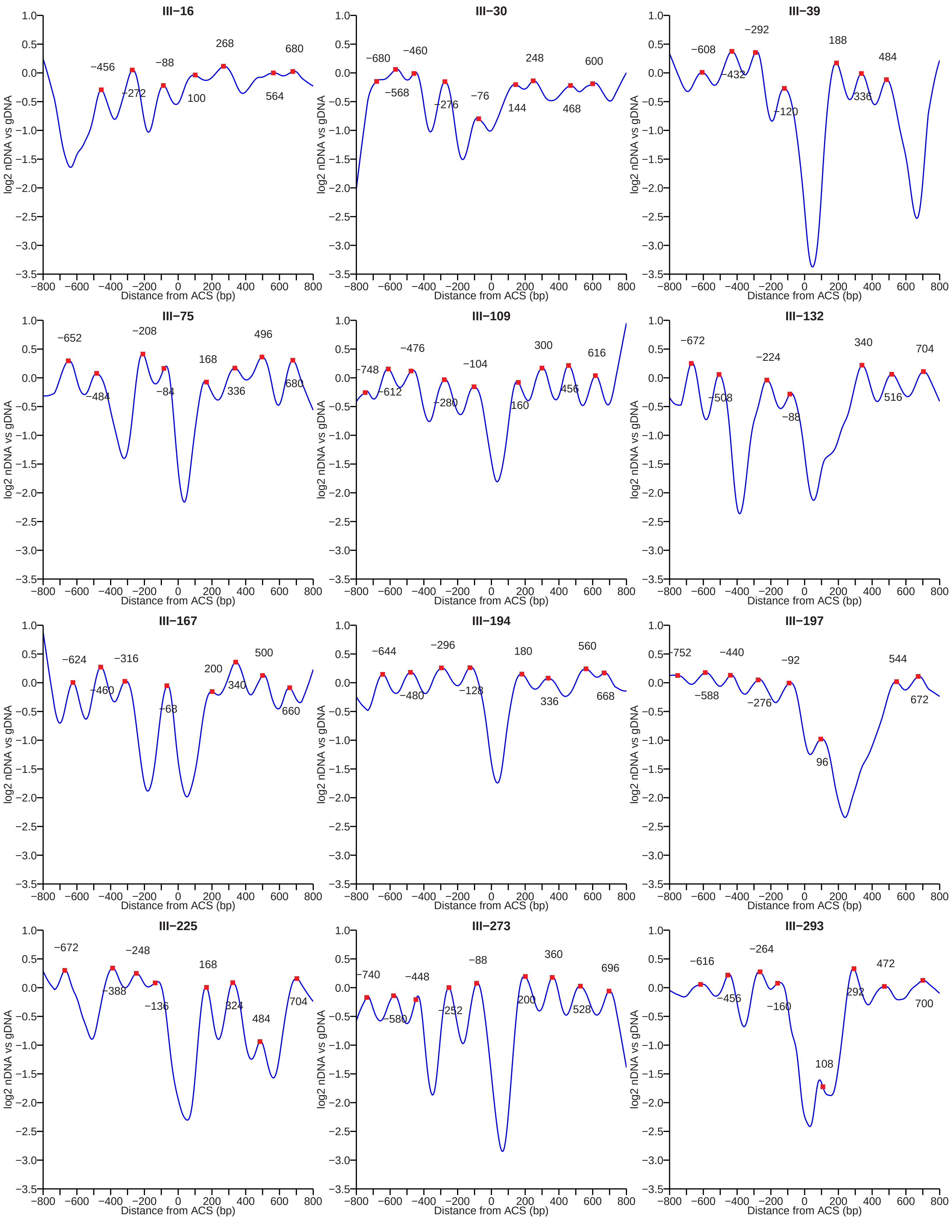
III−16
−456
−272
−88
100
268
564
680
III−30
−680
−568
−460
−276
−76
144
248
468
600
III−39
−608
−432
−292
−120
188
336
484
III−75
−652
−484
−208
−84
168
336
496
680
III−109
−748
−612
−476
−280
−104
160
300
456
616
III−132
−672
−508
−224
−88
340
516
704
III−167
−624
−460
−316
−68
200
340
500
660
III−194
−644
−480
−296
−128
180
336
560
668
III−197
−752
−588
−440
−276
−92
96
544
672
III−225
−672
−388
−248
−136
168
324
484
704
III−273
−740
−580
−448
−252
−88
200
360
528
696
III−293
−616
−456
−264
−160
108
292
472
700
log2 nDNA vs gDNA
Distance from ACS (bp)
1.0
0.5
0.0
−0.5
−1.0
−1.5
−2.0
−2.5
−3.0
−3.5
−800
−600
−400
−200
0
200
400
600
800

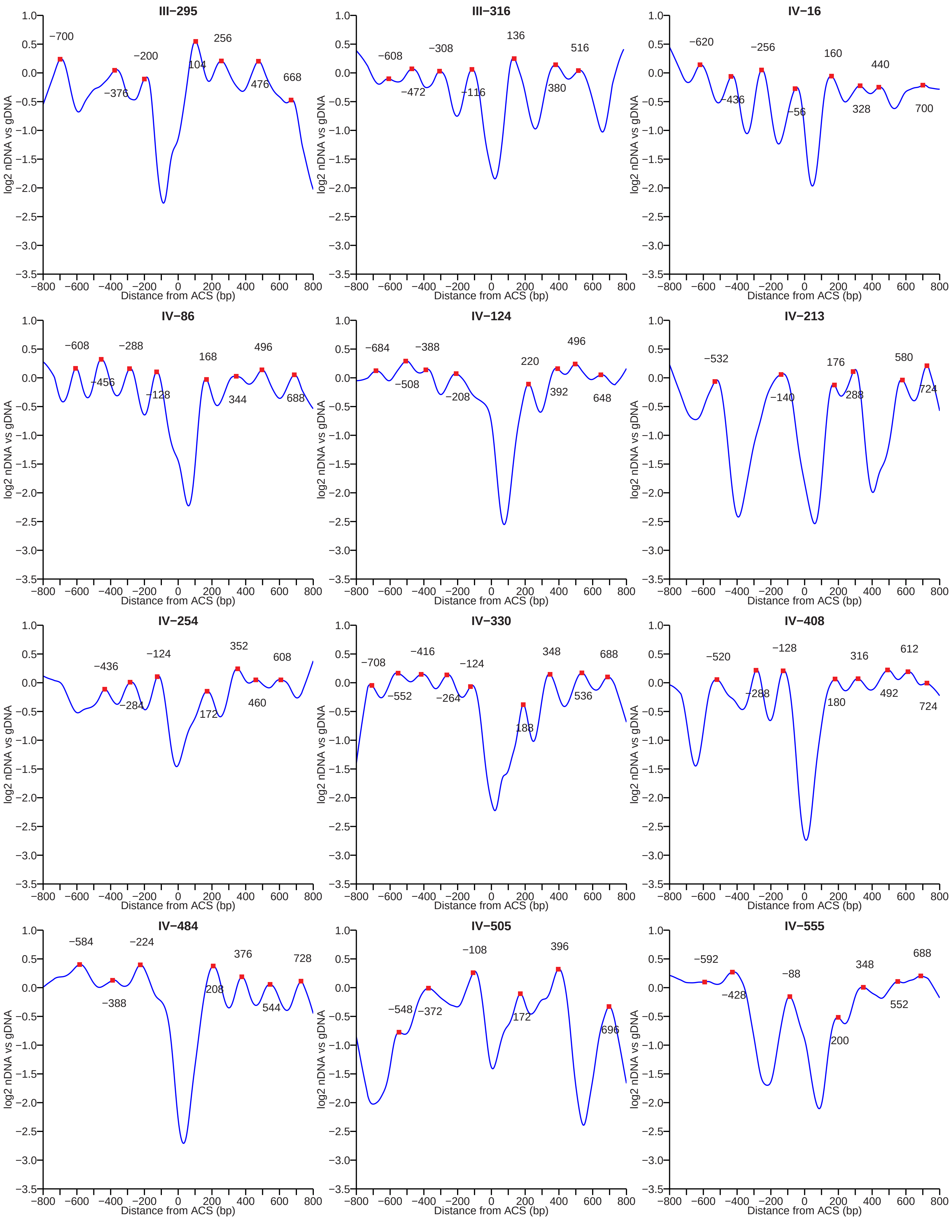
III−295
−700
256
−200
104
−376
476
668
III−316
−608
−308
136
516
−472
−116
380
IV−16
−620
−256
160
440
−436
−56
328
700
IV−86
−608
−288
168
496
−456
−128
344
688
IV−124
−684
−388
496
220
−508
−208
392
648
IV−213
−532
176
580
−140
288
724
IV−254
−436
−124
352
608
−284
172
460
IV−330
−708
−416
−124
348
688
−552
−264
536
188
IV−408
−520
−128
316
612
−288
180
492
724
IV−484
−584
−224
376
728
−388
208
544
IV−505
−108
396
−548
−372
172
696
IV−555
−592
−88
348
688
−428
552
200
log2 nDNA vs gDNA
1.0
0.5
0.0
−0.5
−1.0
−1.5
−2.0
−2.5
−3.0
−3.5
Distance from ACS (bp)
−800
−600
−400
−200
0
200
400
600
800

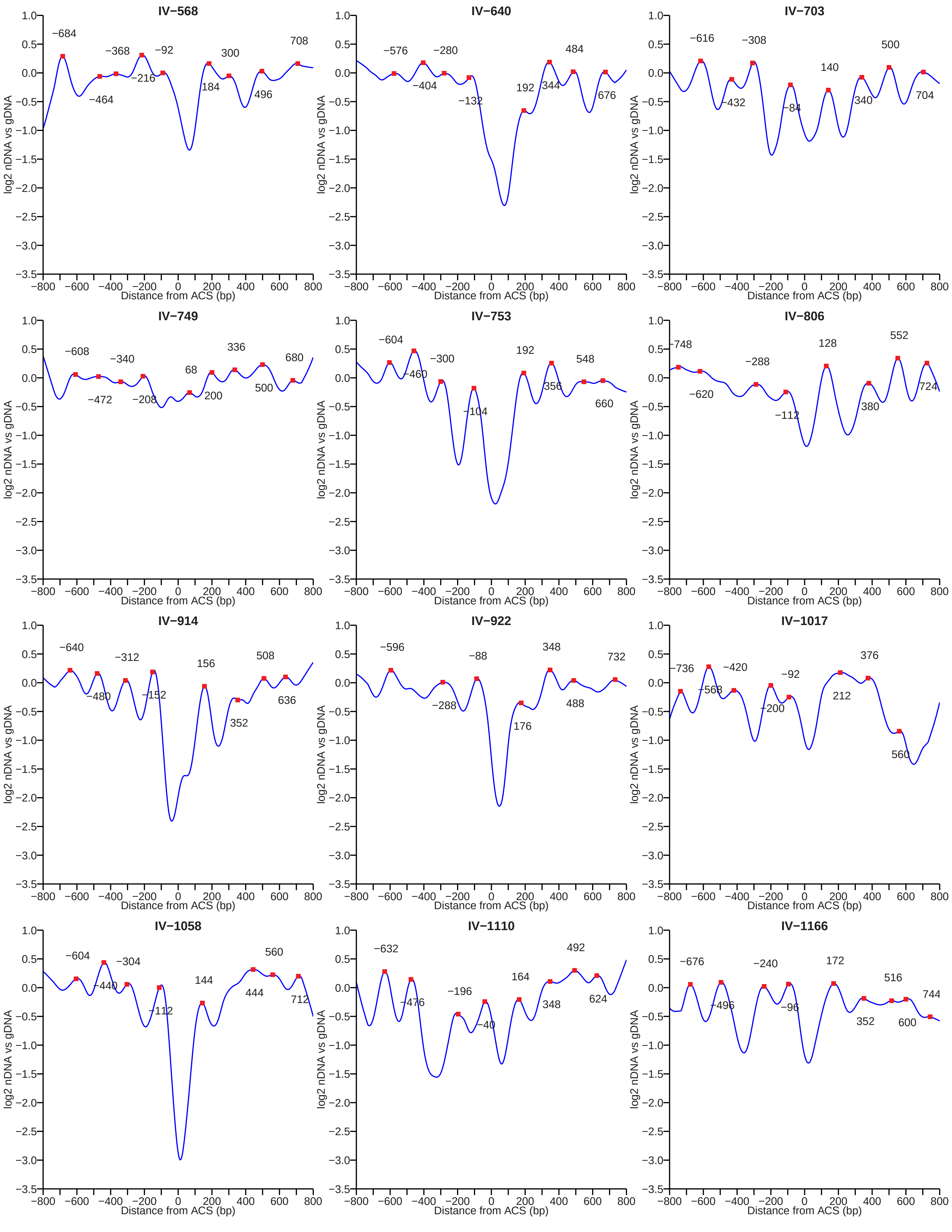
IV−568
−684
−368
−92
300
708
−464
−216
184
496
IV−640
−576
−280
484
−404
−132
192
344
676
IV−703
−616
−308
500
140
−432
−84
340
704
IV−749
−608
−340
336
680
68
−472
−208
200
500
IV−753
−604
−300
192
548
−460
−104
356
660
IV−806
−748
−288
128
552
−620
−112
380
724
IV−914
−640
−312
156
508
−480
−152
352
636
IV−922
−596
−88
348
732
−288
176
488
IV−1017
−736
−420
−92
376
−568
−200
212
560
IV−1058
−604
−304
560
144
−440
−112
444
712
IV−1110
−632
492
−196
164
−476
−40
348
624
IV−1166
−676
−240
172
516
744
−496
−96
352
600
log2 nDNA vs gDNA
Distance from ACS (bp)
1.0
0.5
0.0
−0.5
−1.0
−1.5
−2.0
−2.5
−3.0
−3.5
−800
−600
−400
−200
0
200
400
600
800

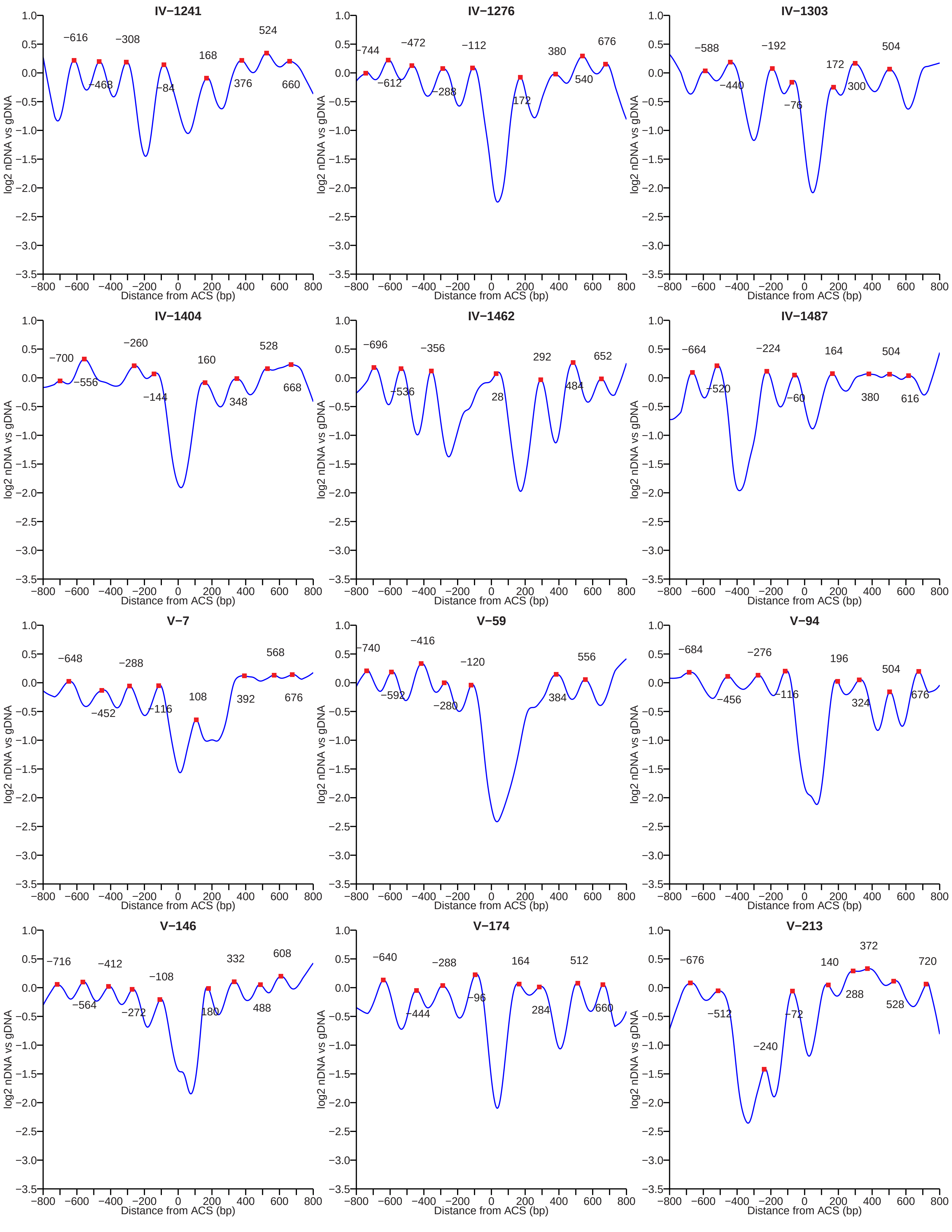
IV−1241
−616
−308
524
168
−468
−84
376
660
log2 nDNA vs gDNA
Distance from ACS (bp)
IV−1276
−744
−472
−112
380
676
−612
−288
172
540
log2 nDNA vs gDNA
Distance from ACS (bp)
IV−1303
−588
−192
504
172
−440
−76
300
log2 nDNA vs gDNA
Distance from ACS (bp)
IV−1404
−700
−260
528
160
−556
−144
348
668
log2 nDNA vs gDNA
Distance from ACS (bp)
IV−1462
−696
−356
292
652
−536
28
484
log2 nDNA vs gDNA
Distance from ACS (bp)
IV−1487
−664
−224
164
504
−520
−60
380
616
log2 nDNA vs gDNA
Distance from ACS (bp)
V−7
−648
−288
568
108
−452
−116
392
676
log2 nDNA vs gDNA
Distance from ACS (bp)
V−59
−740
−416
−120
556
−592
−280
384
log2 nDNA vs gDNA
Distance from ACS (bp)
V−94
−684
−276
196
504
−456
−116
324
676
log2 nDNA vs gDNA
Distance from ACS (bp)
V−146
−716
−412
−108
332
608
−564
−272
180
488
log2 nDNA vs gDNA
Distance from ACS (bp)
V−174
−640
−288
164
512
−444
−96
284
660
log2 nDNA vs gDNA
Distance from ACS (bp)
V−213
−676
372
140
720
−512
−72
288
528
−240
log2 nDNA vs gDNA
Distance from ACS (bp)

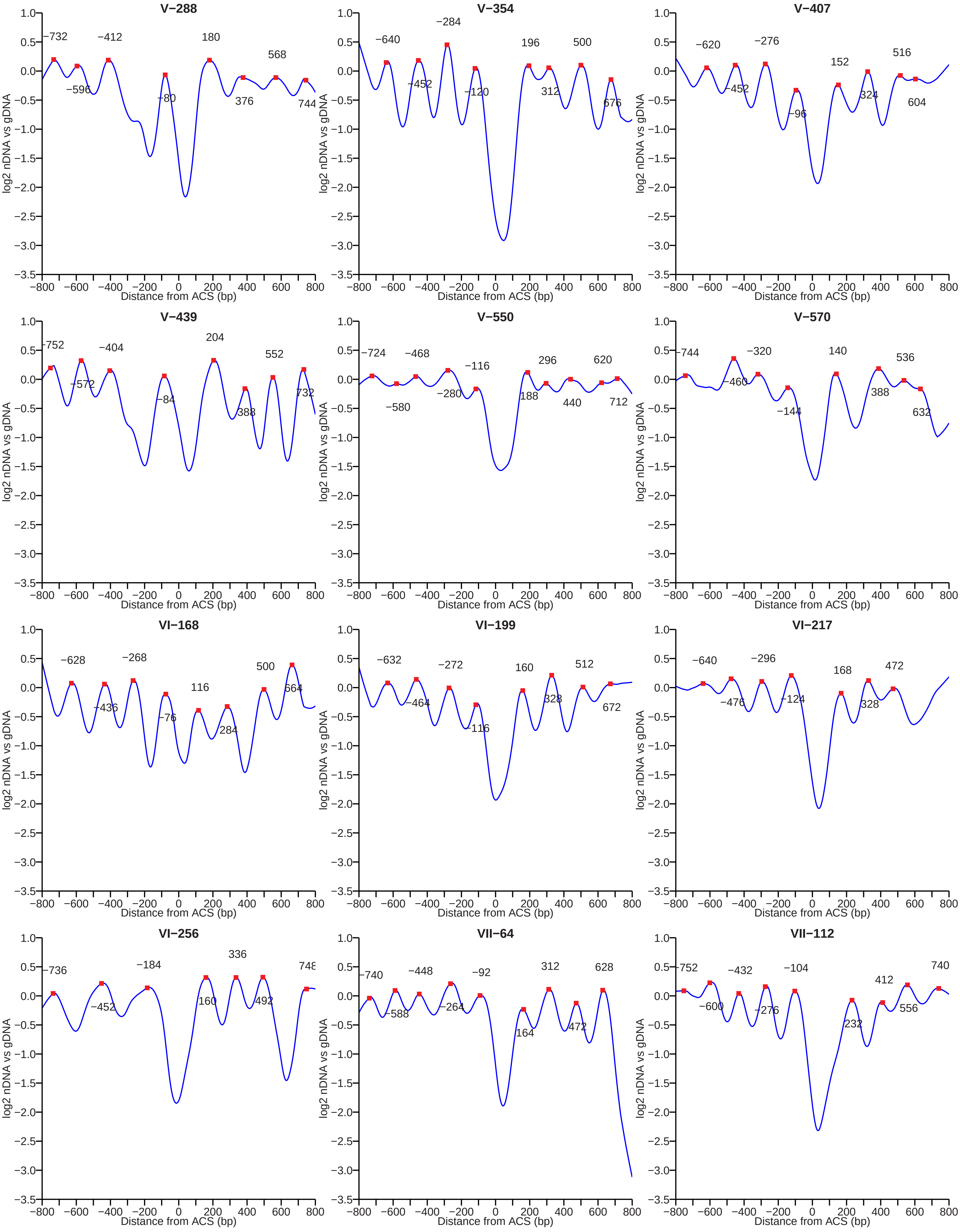
V−288
−732
−412
180
568
−596
−80
376
744
V−354
−284
−640
196
500
−452
−120
312
676
V−407
−620
−276
516
152
−452
324
604
−96
V−439
−752
−404
204
552
−572
732
−84
388
V−550
−724
−468
−116
296
620
188
712
−280
440
−580
V−570
−744
−320
140
536
−460
388
−144
632
VI−168
−628
−268
500
116
664
−436
−76
284
VI−199
−632
−272
160
512
−464
328
672
−116
VI−217
−640
−296
168
472
−476
−124
328
VI−256
−736
−184
336
748
−452
160
492
VII−64
−740
−448
−92
312
628
−588
−264
164
472
VII−112
−752
−432
−104
740
412
−600
−276
556
232
log2 nDNA vs gDNA
Distance from ACS (bp)

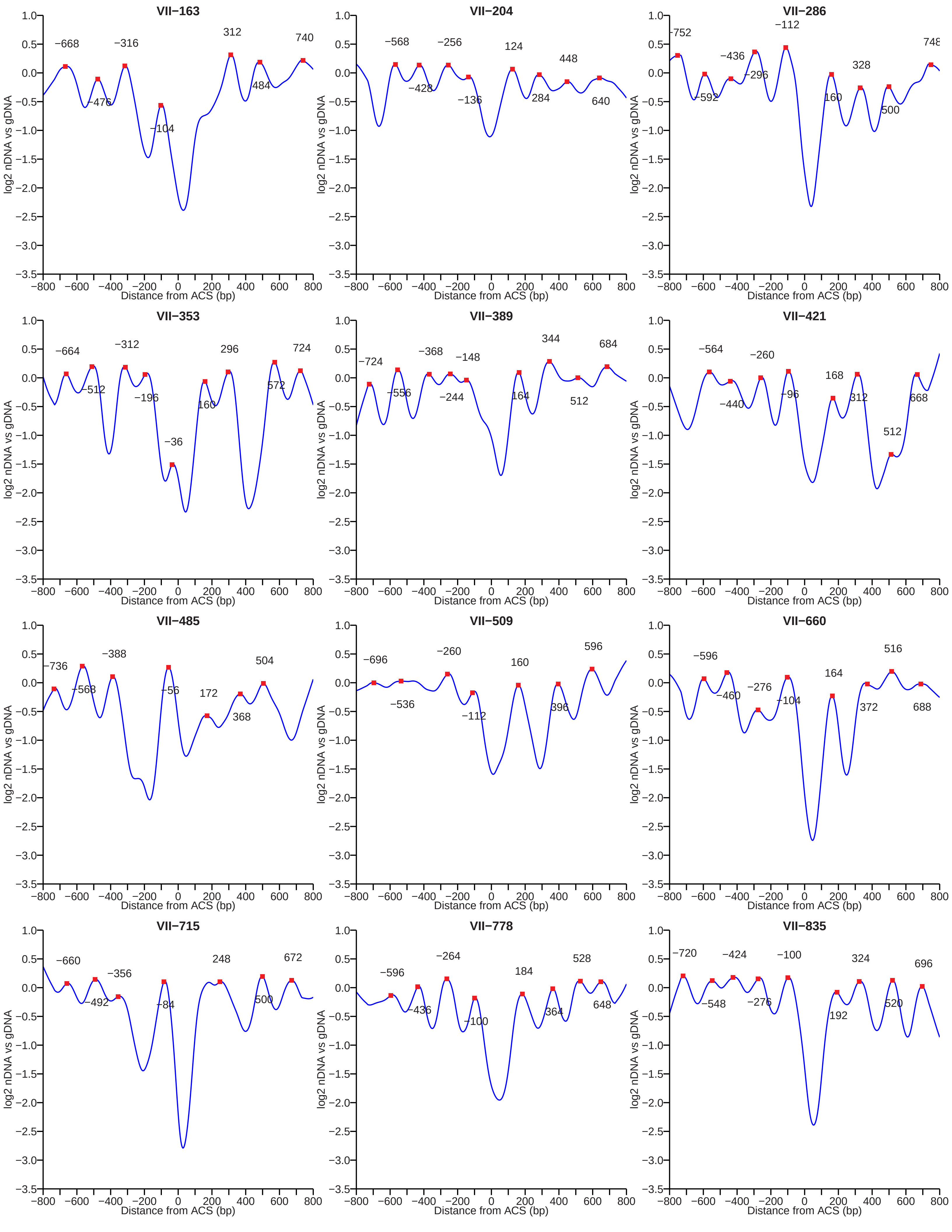

log2 nDNA vs gDNA
Distance from ACS (bp)
VII−163
−668
−316
312
740
−476
−104
484
VII−204
−568
−256
124
448
−428
−136
284
640
VII−286
−752
−112
−436
−296
328
748
−592
160
500
VII−353
−664
−312
296
724
−512
−196
−36
160
572
VII−389
−724
−368
−148
344
684
−556
−244
164
512
VII−421
−564
−260
168
−440
−96
312
668
512
VII−485
−736
−388
504
−568
−56
172
368
VII−509
−696
−260
160
596
−536
−112
396
VII−660
−596
164
516
−460
−276
−104
372
688
VII−715
−660
−356
248
672
−492
−84
500
VII−778
−596
−264
184
528
−436
−100
364
648
VII−835
−720
−424
−100
324
696
−548
−276
192
520

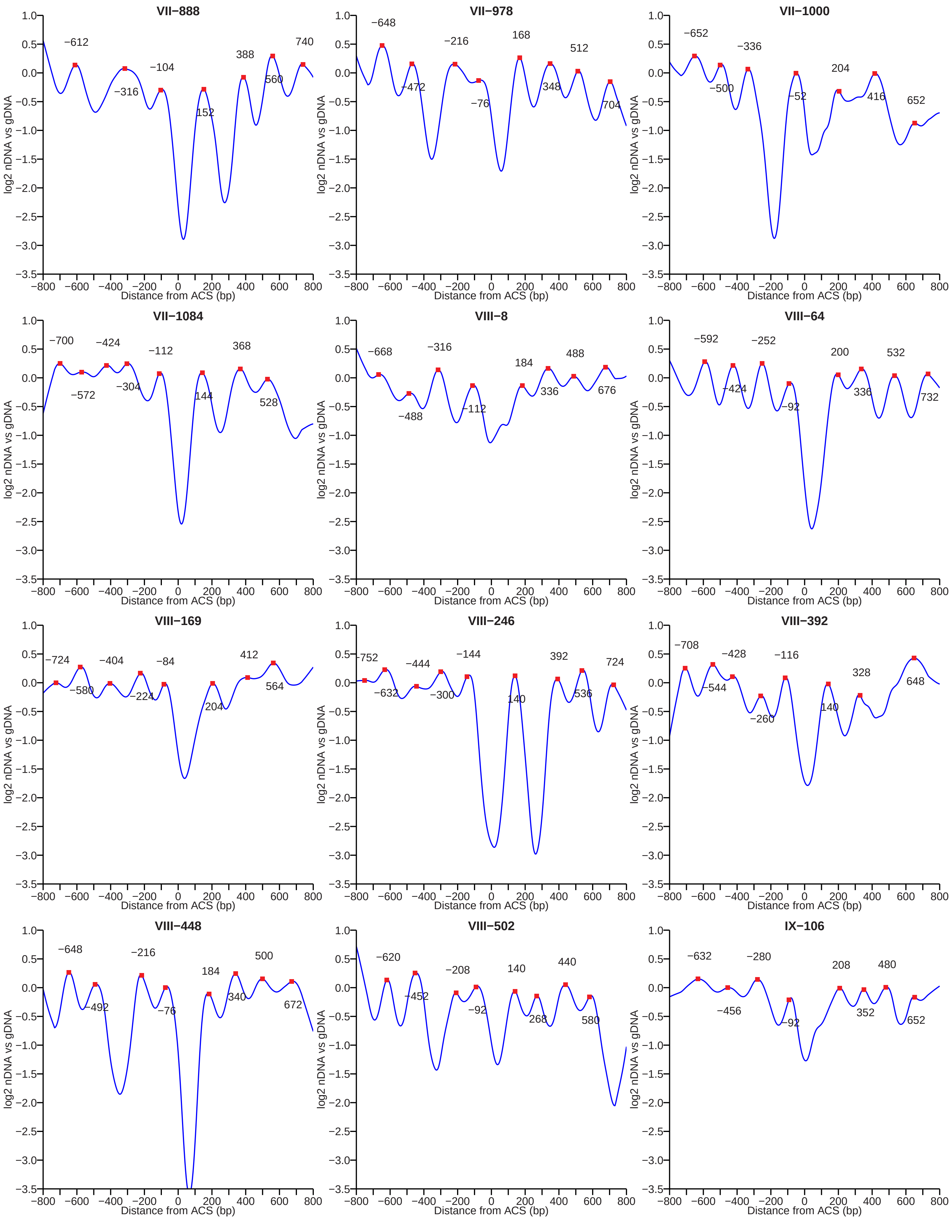
VII−888
−612
−316
−104
152
388
560
740
VII−978
−648
−472
−216
−76
168
348
512
704
VII−1000
−652
−500
−336
−52
204
416
652
VII−1084
−700
−572
−424
−304
−112
144
368
528
VIII−8
−668
−488
−316
−112
184
336
488
676
VIII−64
−592
−424
−252
−92
200
336
532
732
VIII−169
−724
−580
−404
−224
−84
204
412
564
VIII−246
−752
−632
−444
−300
−144
140
392
536
724
VIII−392
−708
−544
−428
−260
−116
140
328
648
VIII−448
−648
−492
−216
−76
184
340
500
672
VIII−502
−620
−452
−208
−92
140
268
440
580
IX−106
−632
−456
−280
−92
208
352
480
652
log2 nDNA vs gDNA
Distance from ACS (bp)

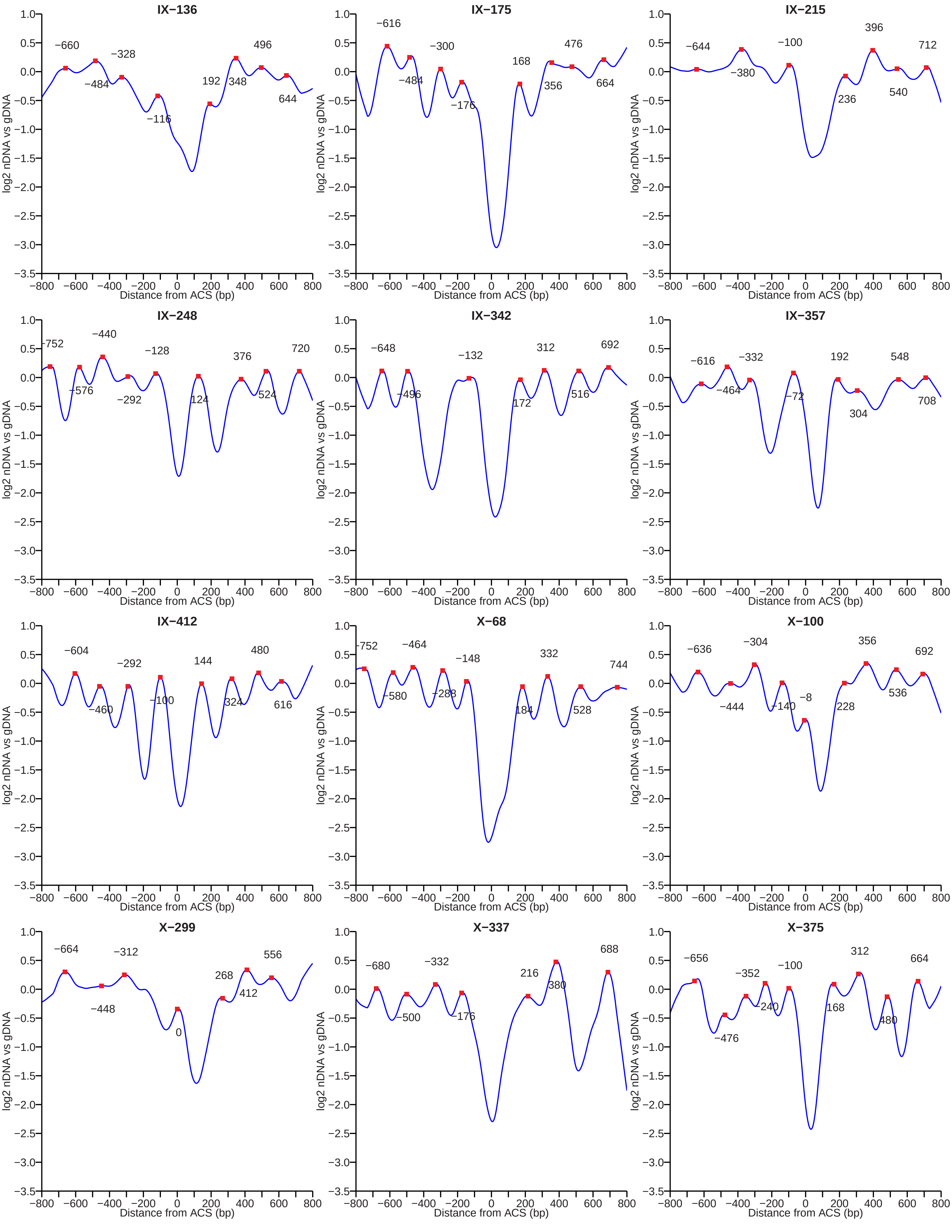
IX−136
−660
−484
−328
−116
192
348
496
644
IX−175
−616
−484
−300
−176
168
356
476
664
IX−215
−644
−380
−100
236
396
540
712
IX−248
−752
−576
−440
−292
−128
124
376
524
720
IX−342
−648
−496
−132
172
312
516
692
IX−357
−616
−464
−332
−72
192
304
548
708
IX−412
−604
−460
−292
−100
144
324
480
616
X−68
−752
−580
−464
−288
−148
184
332
528
744
X−100
−636
−444
−304
−140
−8
228
356
536
692
X−299
−664
−448
−312
0
268
412
556
X−337
−680
−500
−332
−176
216
380
688
X−375
−656
−476
−352
−240
−100
168
312
480
664
log2 nDNA vs gDNA
Distance from ACS (bp)

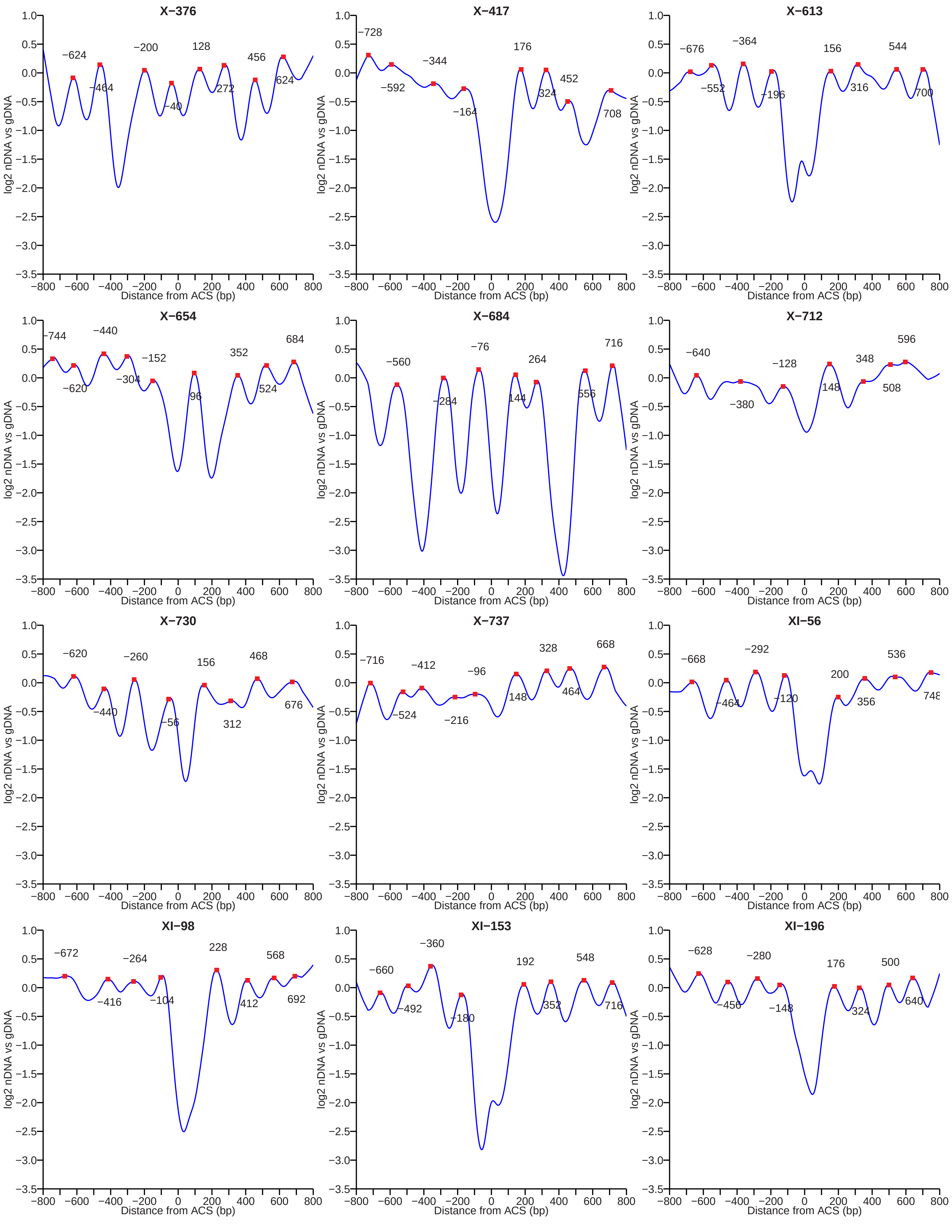
X−376
−624
−464
−200
−40
128
272
456
624
X−417
−728
−592
−344
−164
176
324
452
708
X−613
−676
−552
−364
−196
156
316
544
700
X−654
−744
−620
−440
−304
−152
96
352
524
684
X−684
−560
−284
−76
144
264
556
716
X−712
−640
−380
−128
148
348
508
596
X−730
−620
−440
−260
−56
156
312
468
676
X−737
−716
−524
−412
−216
−96
148
328
464
668
XI−56
−668
−464
−292
−120
200
356
536
748
XI−98
−672
−416
−264
−104
228
412
568
692
XI−153
−660
−492
−360
−180
192
352
548
716
XI−196
−628
−456
−280
−148
176
324
500
640
log2 nDNA vs gDNA
Distance from ACS (bp)
1.0
0.5
0.0
−0.5
−1.0
−1.5
−2.0
−2.5
−3.0
−3.5
−800
−600
−400
−200
0
200
400
600
800

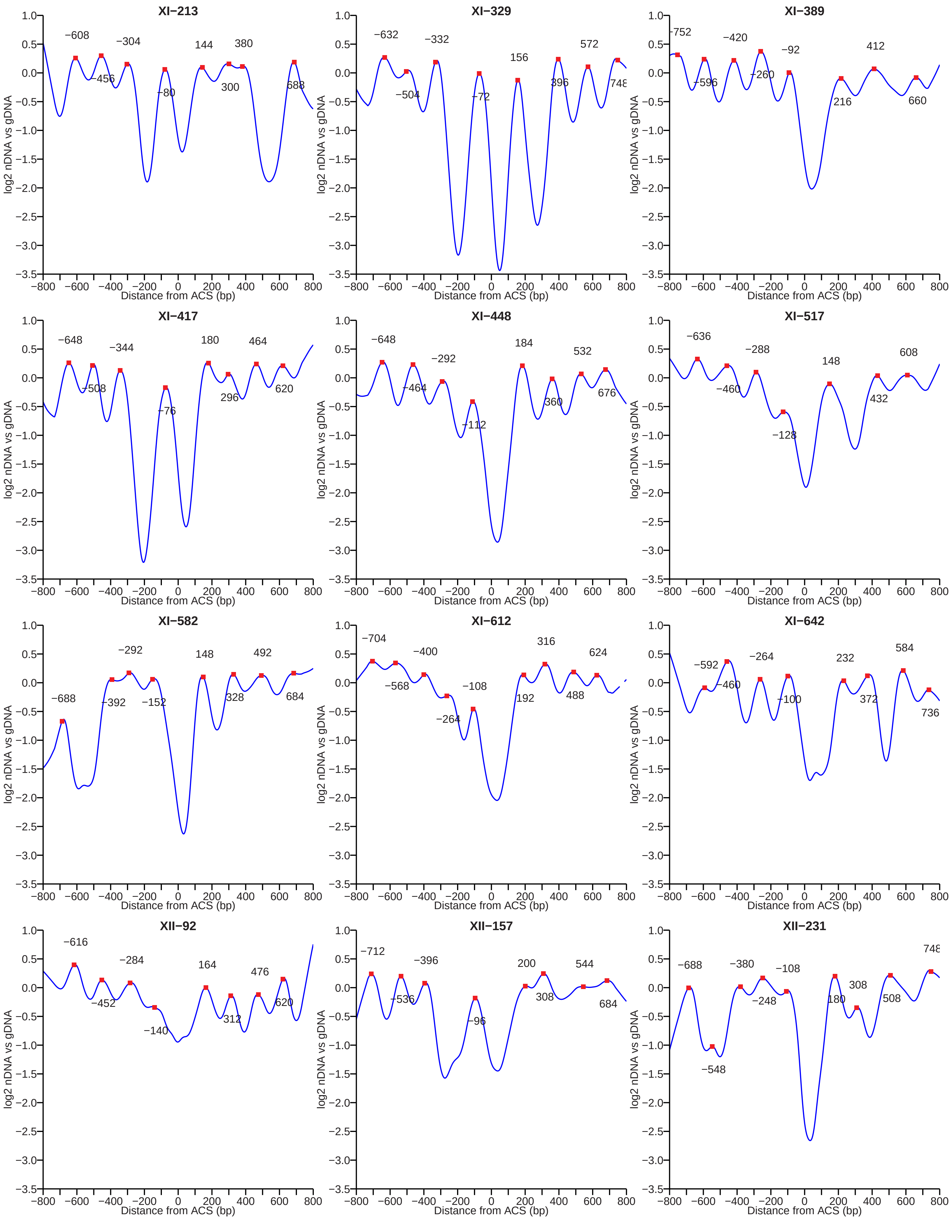
XI−213
−608
−456
−304
−80
144
300
380
688
XI−329
−632
−504
−332
−72
156
396
572
748
XI−389
−752
−596
−420
−260
−92
216
412
660
XI−417
−648
−508
−344
−76
180
296
464
620
XI−448
−648
−464
−292
−112
184
360
532
676
XI−517
−636
−460
−288
−128
148
432
608
XI−582
−688
−392
−292
−152
148
328
492
684
XI−612
−704
−568
−400
−264
−108
192
316
488
624
XI−642
−592
−460
−264
−100
232
372
584
736
XII−92
−616
−452
−284
−140
164
312
476
620
XII−157
−712
−536
−396
−96
200
308
544
684
XII−231
−688
−548
−380
−248
−108
180
308
508
748
log2 nDNA vs gDNA
Distance from ACS (bp)

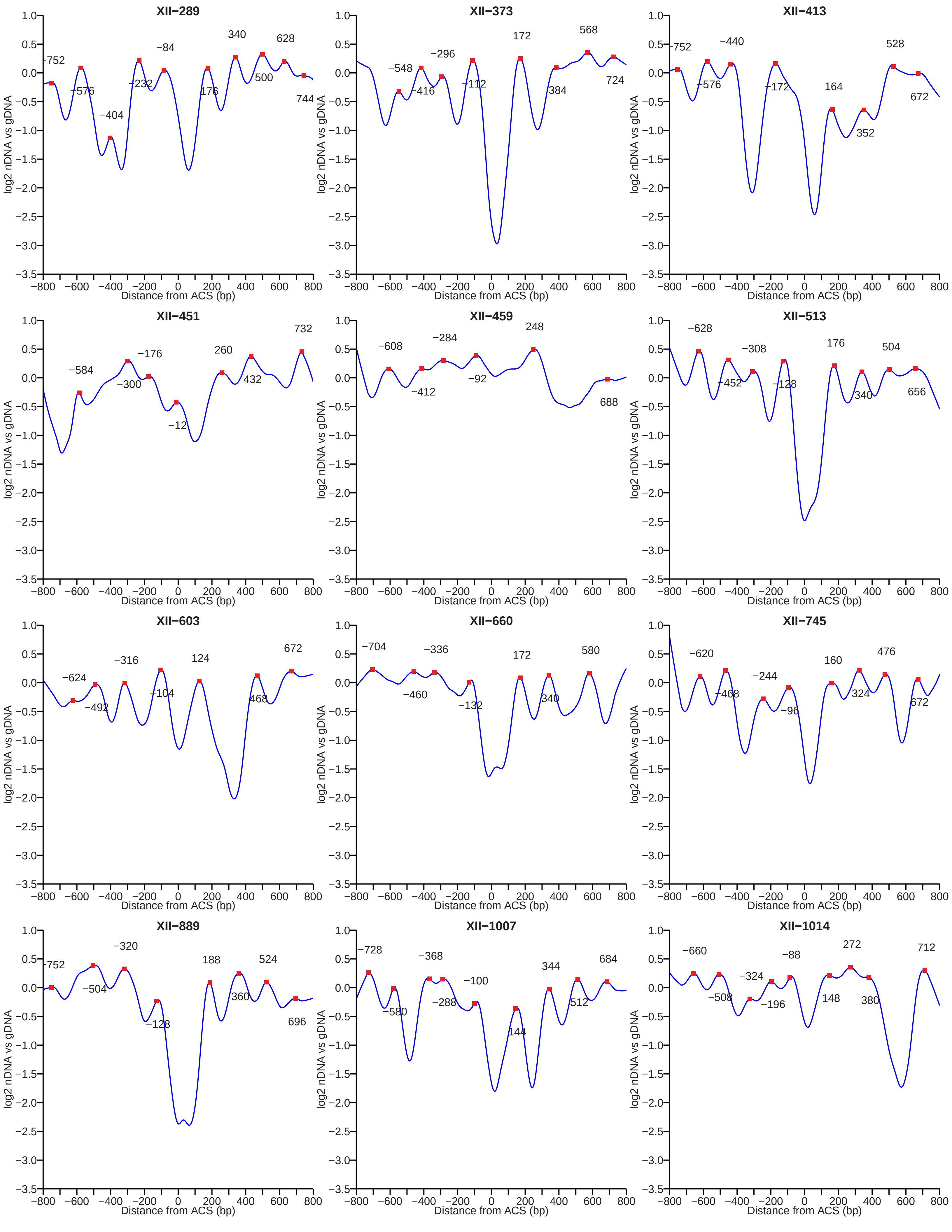
XII−289
−752
−576
−404
−232
−84
176
340
500
628
744
XII−373
−548
−416
−296
−112
172
384
568
724
XII−413
−752
−576
−440
−172
164
352
528
672
XII−451
−584
−300
−176
−12
260
432
732
XII−459
−608
−412
−284
−92
248
688
XII−513
−628
−452
−308
−128
176
340
504
656
XII−603
−624
−492
−316
−104
124
468
672
XII−660
−704
−460
−336
−132
172
340
580
XII−745
−620
−468
−244
−96
160
324
476
672
XII−889
−752
−504
−320
−128
188
360
524
696
XII−1007
−728
−580
−368
−288
−100
144
344
512
684
XII−1014
−660
−508
−324
−196
−88
148
272
380
712
log2 nDNA vs gDNA
Distance from ACS (bp)

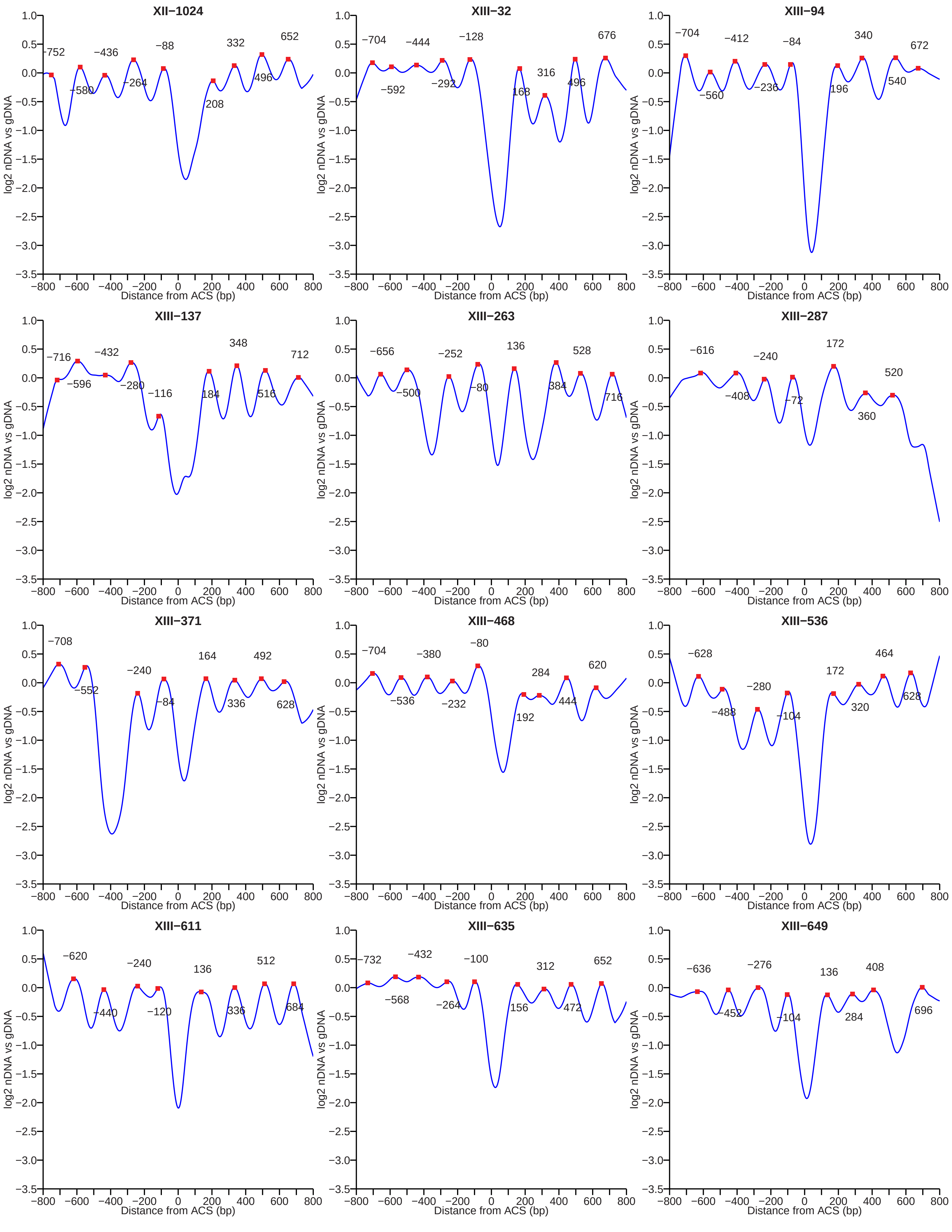

XII−1024
−752
−580
−436
−264
−88
208
332
496
652
XIII−32
−704
−592
−444
−292
−128
168
316
496
676
XIII−94
−704
−560
−412
−236
−84
196
340
540
672
XIII−137
−716
−596
−432
−280
−116
184
348
516
712
XIII−263
−656
−500
−252
−80
136
384
528
716
XIII−287
−616
−408
−240
−72
172
360
520
XIII−371
−708
−552
−240
−84
164
336
492
628
XIII−468
−704
−536
−380
−232
−80
192
284
444
620
XIII−536
−628
−488
−280
−104
172
320
464
628
XIII−611
−620
−440
−240
−120
136
336
512
684
XIII−635
−732
−568
−432
−264
−100
156
312
472
652
XIII−649
−636
−452
−276
−104
136
284
408
696
log2 nDNA vs gDNA
Distance from ACS (bp)

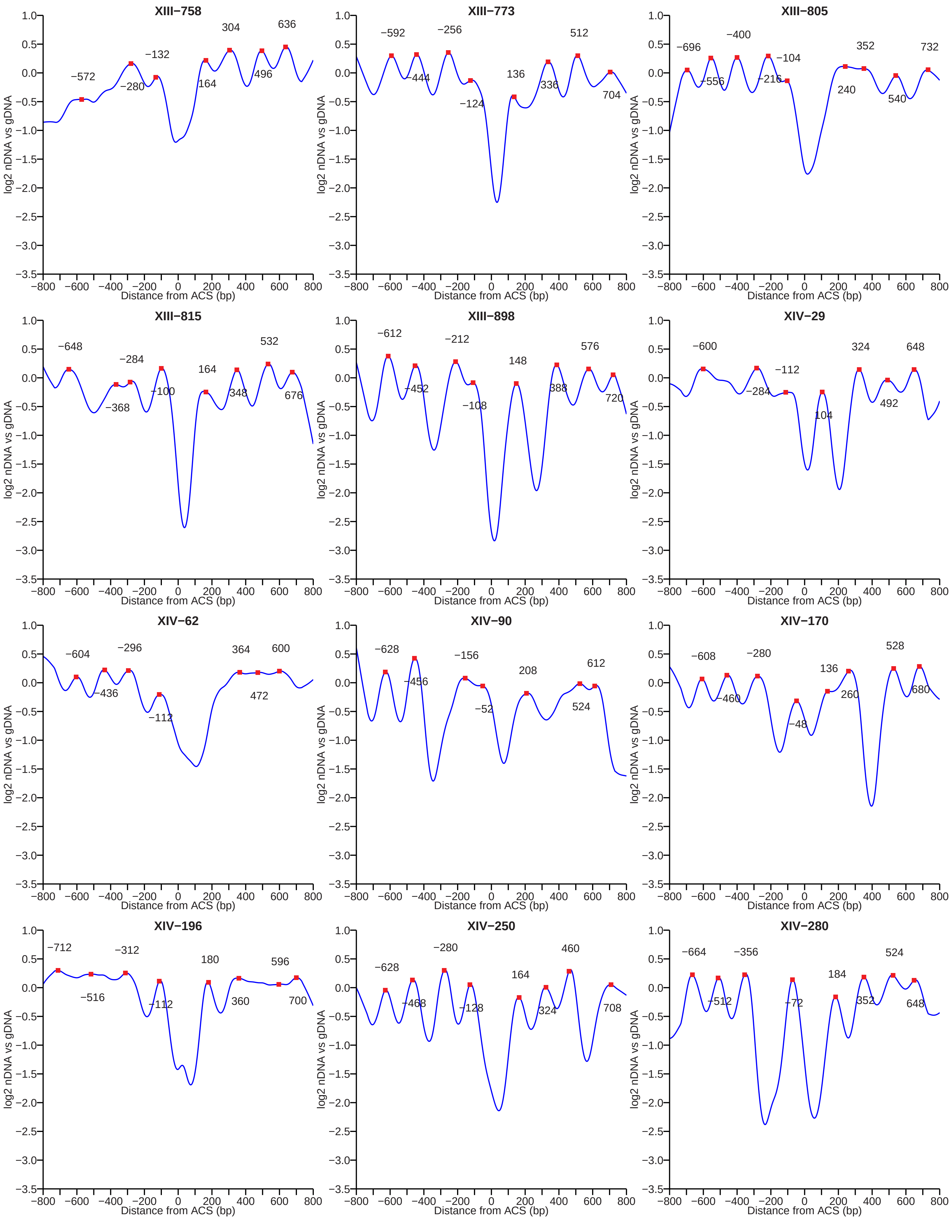
XIII−758
−572
−280
−132
164
304
496
636
XIII−773
−592
−444
−256
−124
136
336
512
704
XIII−805
−696
−556
−400
−216
−104
240
352
540
732
XIII−815
−648
−368
−284
−100
164
348
532
676
XIII−898
−612
−452
−212
−108
148
388
576
720
XIV−29
−600
−284
−112
104
324
492
648
XIV−62
−604
−436
−296
−112
364
472
600
XIV−90
−628
−456
−156
−52
208
524
612
XIV−170
−608
−460
−280
−48
136
260
528
680
XIV−196
−712
−516
−312
−112
180
360
596
700
XIV−250
−628
−468
−280
−128
164
324
460
708
XIV−280
−664
−512
−356
−72
184
352
524
648
log2 nDNA vs gDNA
Distance from ACS (bp)

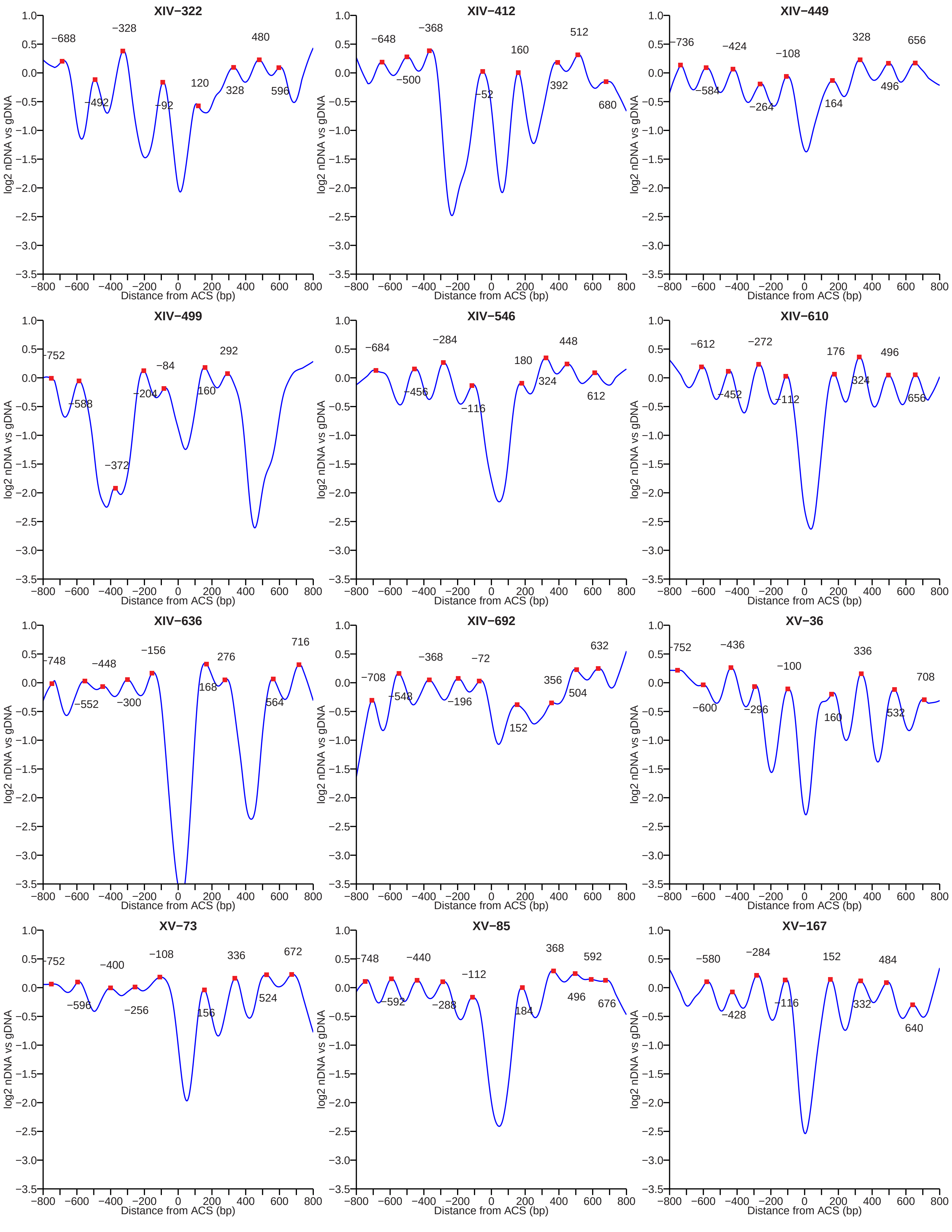

XIV−322
−688
−328
−492
−92
120
328
480
596
XIV−412
−648
−368
−500
−52
160
392
512
680
XIV−449
−736
−584
−424
−264
−108
164
328
496
656
XIV−499
−752
−588
−372
−204
−84
160
292
XIV−546
−684
−456
−284
−116
180
324
448
612
XIV−610
−612
−452
−272
−112
176
324
496
656
XIV−636
−748
−552
−448
−300
−156
168
276
564
716
XIV−692
−708
−548
−368
−196
−72
152
356
504
632
XV−36
−752
−600
−436
−296
−100
160
336
532
708
XV−73
−752
−596
−400
−256
−108
156
336
524
672
XV−85
−748
−592
−440
−288
−112
184
368
496
592
676
XV−167
−580
−428
−284
−116
152
332
484
640
log2 nDNA vs gDNA
Distance from ACS (bp)

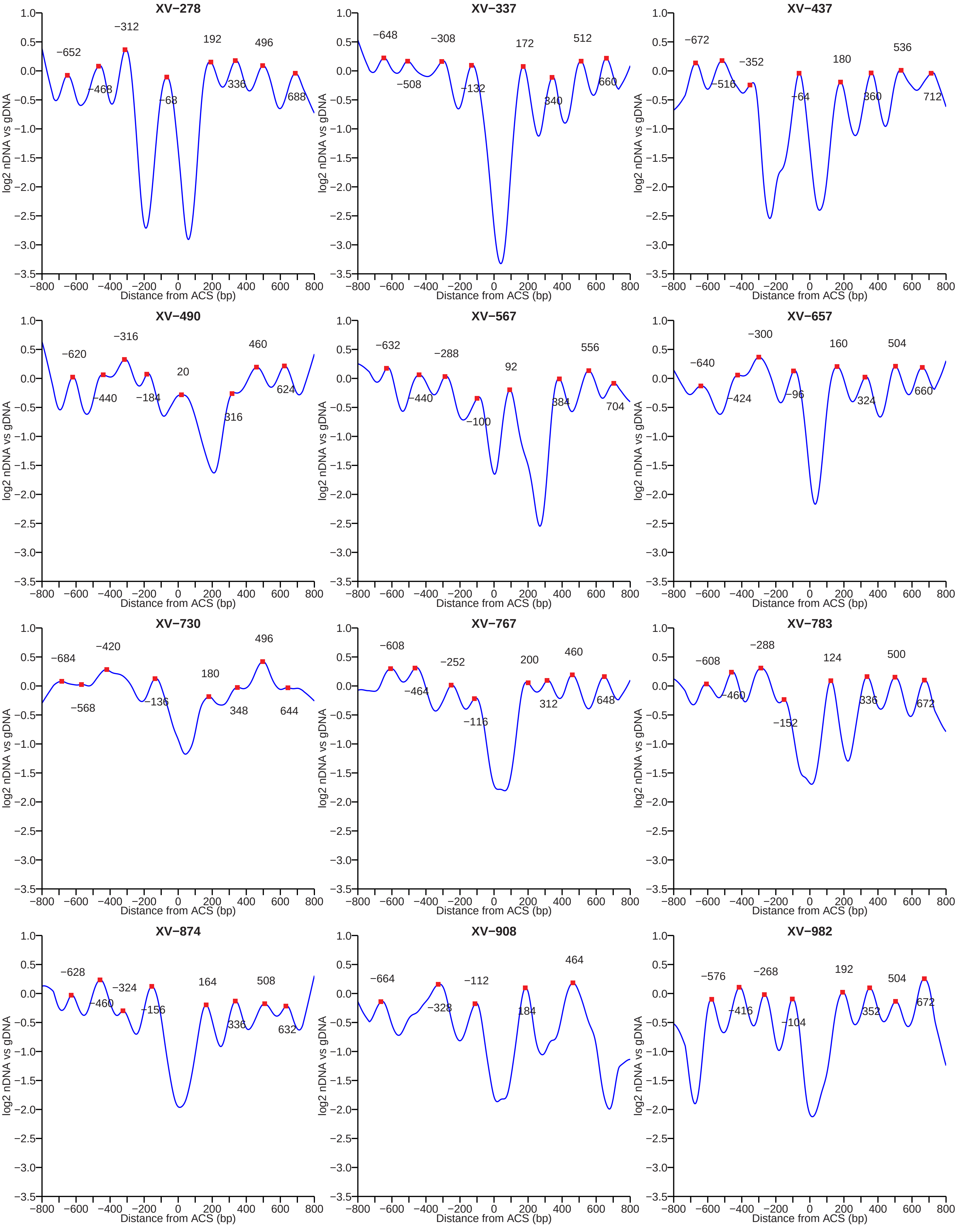
XV−278
−652
−468
−312
−68
192
336
496
688
XV−337
−648
−508
−308
−132
172
340
512
660
XV−437
−672
−516
−352
−64
180
360
536
712
XV−490
−620
−440
−316
−184
20
316
460
624
XV−567
−632
−440
−288
−100
92
384
556
704
XV−657
−640
−424
−300
−96
160
324
504
660
XV−730
−684
−568
−420
−136
180
348
496
644
XV−767
−608
−464
−252
−116
200
312
460
648
XV−783
−608
−460
−288
−152
124
336
500
672
XV−874
−628
−460
−324
−156
164
336
508
632
XV−908
−664
−328
−112
184
464
XV−982
−576
−416
−268
−104
192
352
504
672
log2 nDNA vs gDNA
Distance from ACS (bp)

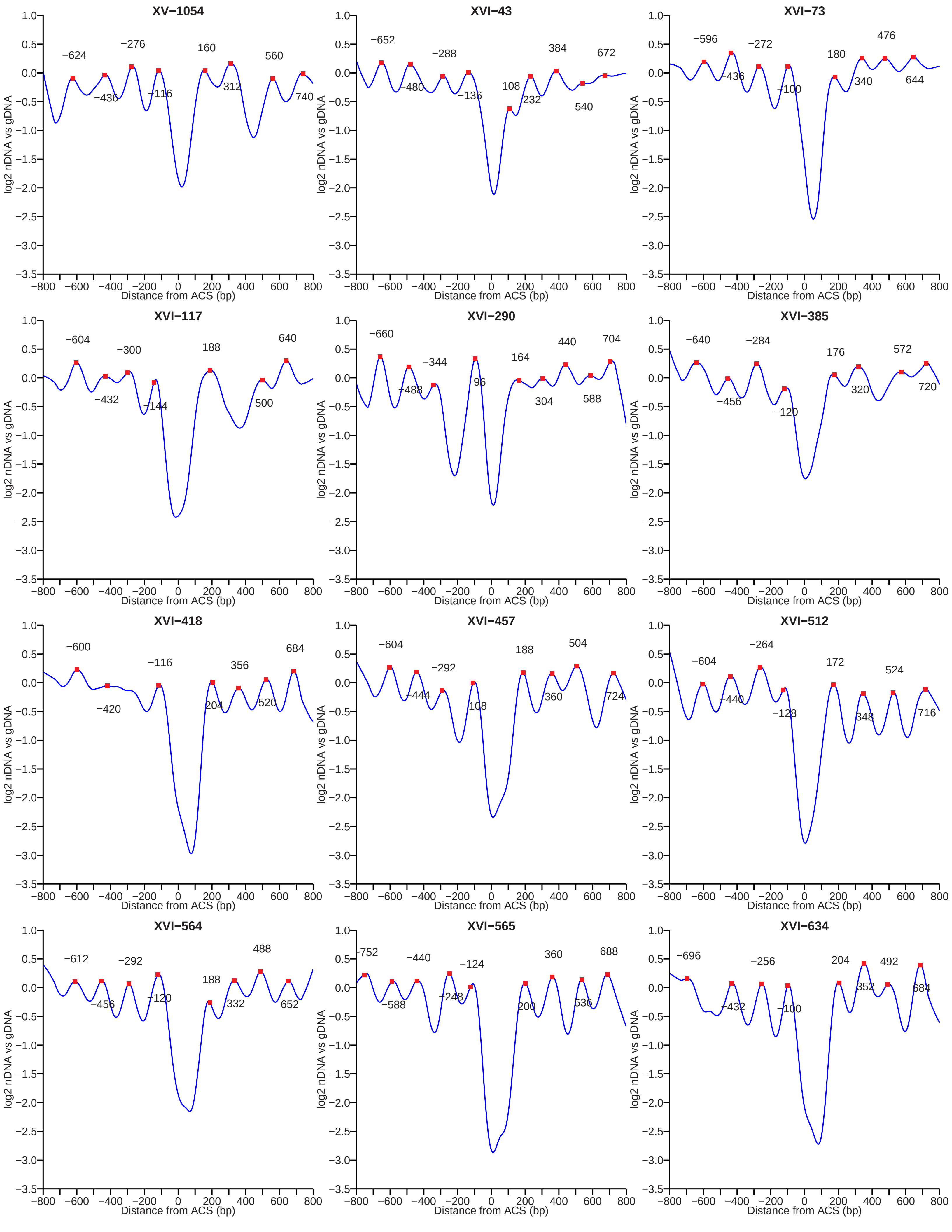

XV−1054
−624
−436
−276
−116
160
312
560
740
XVI−43
−652
−480
−288
−136
108
232
384
540
672
XVI−73
−596
−436
−272
−100
180
340
476
644
XVI−117
−604
−432
−300
−144
188
500
640
XVI−290
−660
−488
−344
−96
164
304
440
588
704
XVI−385
−640
−456
−284
−120
176
320
572
720
XVI−418
−600
−420
−116
204
356
520
684
XVI−457
−604
−444
−292
−108
188
360
504
724
XVI−512
−604
−440
−264
−128
172
348
524
716
XVI−564
−612
−456
−292
−120
188
332
488
652
XVI−565
−752
−588
−440
−248
−124
200
360
536
688
XVI−634
−696
−432
−256
−100
204
352
492
684
log2 nDNA vs gDNA
Distance from ACS (bp)

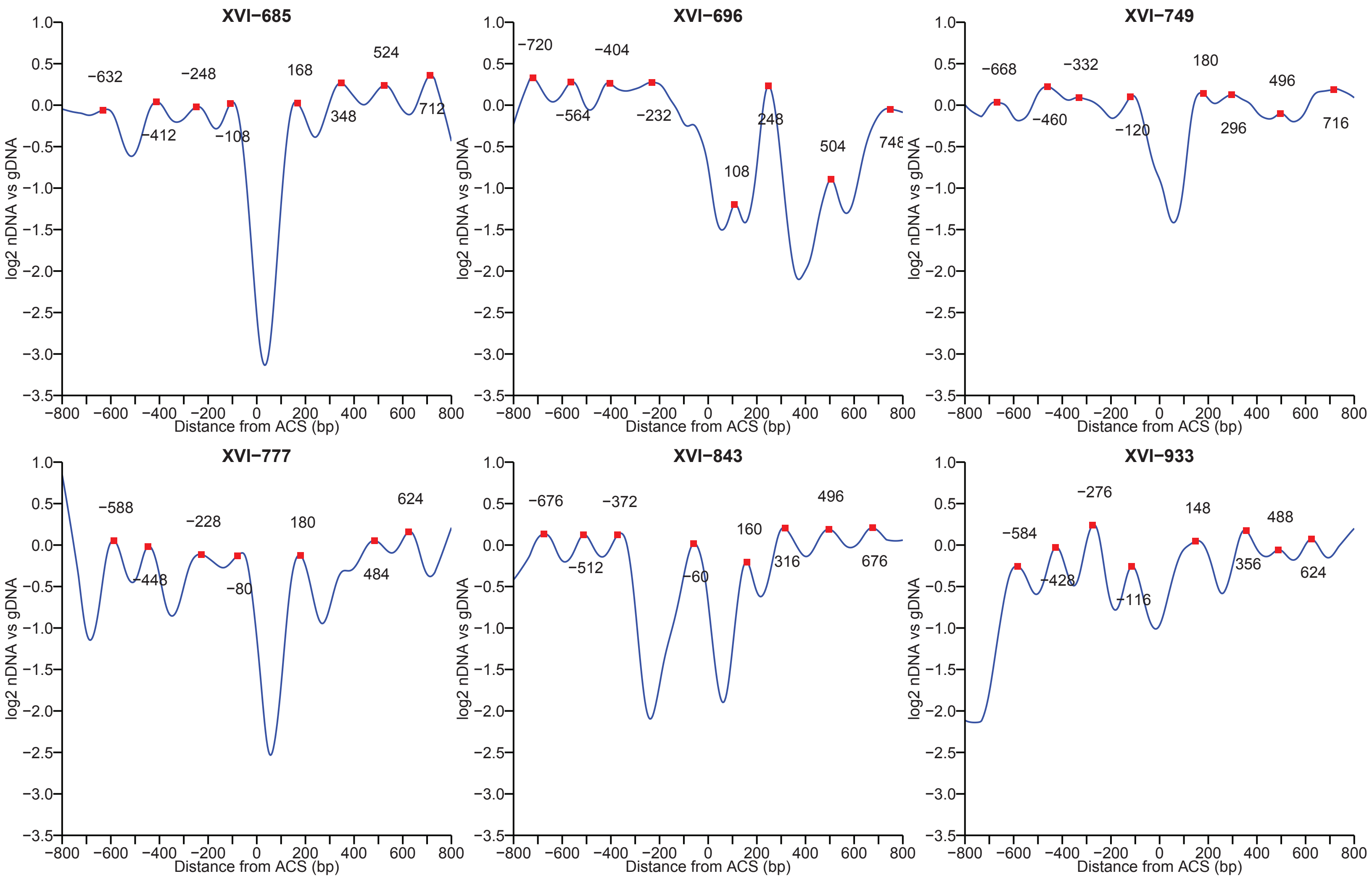

Figure S3. ACS-centered nucleosome profiles for each origin in the wild-type dataset. Individual origins were LOESS smoothed using a span which included 35 probes. The locations of nucleosome midpoints are indicated (▪) and their locations relative to the ACS are indicated. Origin names are based on the naming conventions of OriDB [9].
