# Supplementary material for: Diversity of Eukaryotic DNA Replication Origins Revealed by Genome-Wide Analysis of Chromatin Structure
Source: PLoS Genet. 2010 Sep 2;6(9):e1001092. doi: 10.1371/journal.pgen.1001092 (PMC2932696; doi:10.1371/journal.pgen.1001092)
Supplement: Figure S4 — Flow cytometric analysis of DNA content during Orc2 depletion. (0.51 MB PDF) [file pgen.1001092.s004.pdf]

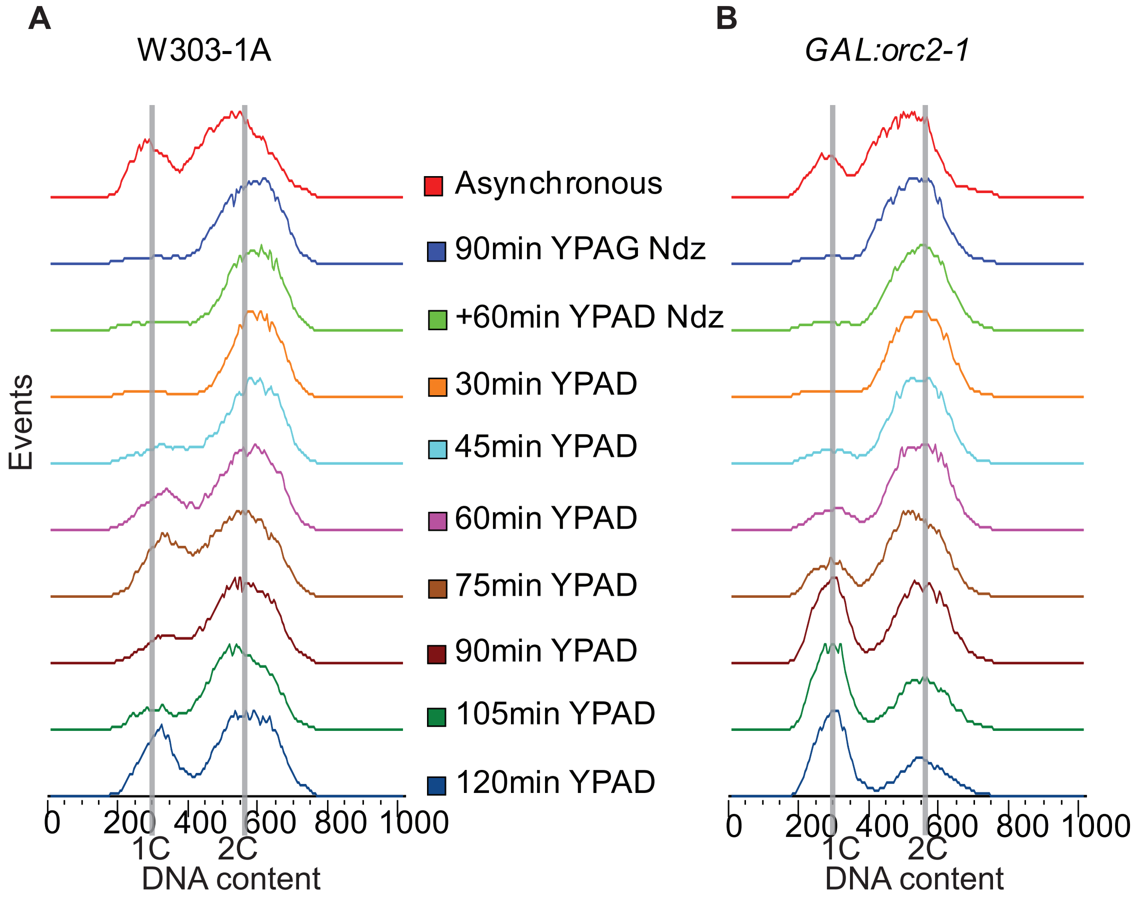

Figure S4. Flow cytometric analysis of DNA content during Orc2 depletion. A. The wild type control strain. B. The Orc2 depletion strain. Both strains were arrested in G2 in nocodazole (NDZ) in galactose medium (YPAG) to allow expression of Orc2. Glucose was added (YPAD) to shut off Orc2. After 60 minutes cells were released into the cell cycle in glucose to continually repress Orc2 expression. The wild type cells enter mitosis between 45 and 60 minutes post-release, as evidenced by the appearance of 1C cells (corresponding to G1 cells). They then progress rapidly through S phase, arriving at a 2C (G2) DNA content by 90 to 105 minutes. By contrast, after passing through mitosis, the *GAL:orc2-1* cells accumulate in G1, indicating that they are unable to enter S phase due to the absence of Orc2. Cells were cross-linked at 120 minutes post-release for preparation of nucleosomal DNA.
