# Supplementary material for: Diversity of Eukaryotic DNA Replication Origins Revealed by Genome-Wide Analysis of Chromatin Structure
Source: PLoS Genet. 2010 Sep 2;6(9):e1001092. doi: 10.1371/journal.pgen.1001092 (PMC2932696; doi:10.1371/journal.pgen.1001092)
Supplement: Figure S5 — NDR width distributions for wild type S288c and W303. (0.18 MB PDF) [file pgen.1001092.s005.pdf]

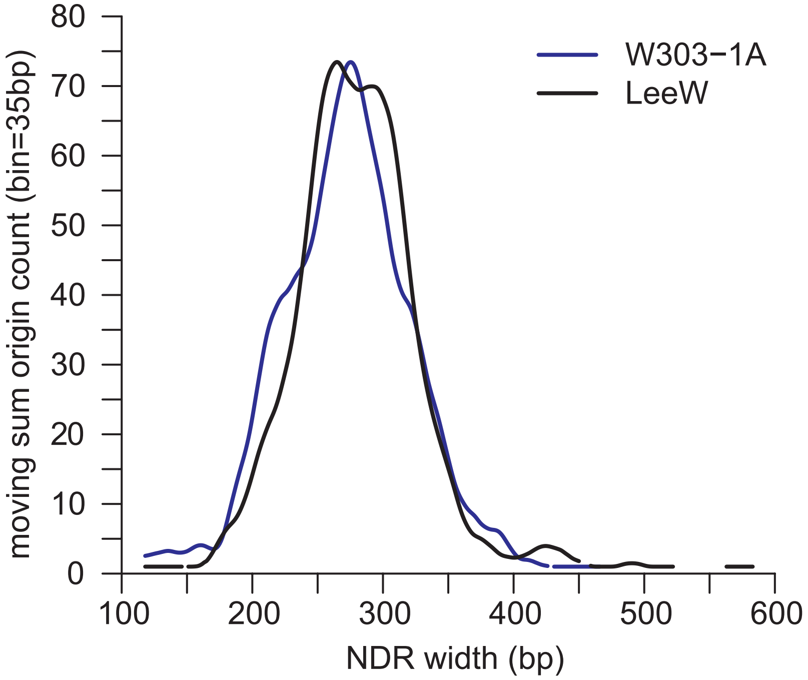

Figure S5. Moving sum comparison of wild-type nucleosome depleted regions (NDRs). NDR width corresponds to the distance between the two nucleosome centers on either side of the ACS. The moving sum NDR width was determined using a range of 100 to 600 bp and a moving window of 35 bp. 90% of origins in the Lee et al. dataset have NDR widths between 212 and 364 bp. The W303-1A wild-type strain had 90% of origins with widths between 196 and 352 bp.
