# Supplementary material for: Diversity of Eukaryotic DNA Replication Origins Revealed by Genome-Wide Analysis of Chromatin Structure
Source: PLoS Genet. 2010 Sep 2;6(9):e1001092. doi: 10.1371/journal.pgen.1001092 (PMC2932696; doi:10.1371/journal.pgen.1001092)
Supplement: Figure S6 — ACS-centered nucleosome profiles for each origin in GAL:orc2 and wild type control. (1.53 MB PDF) [file pgen.1001092.s006.pdf]

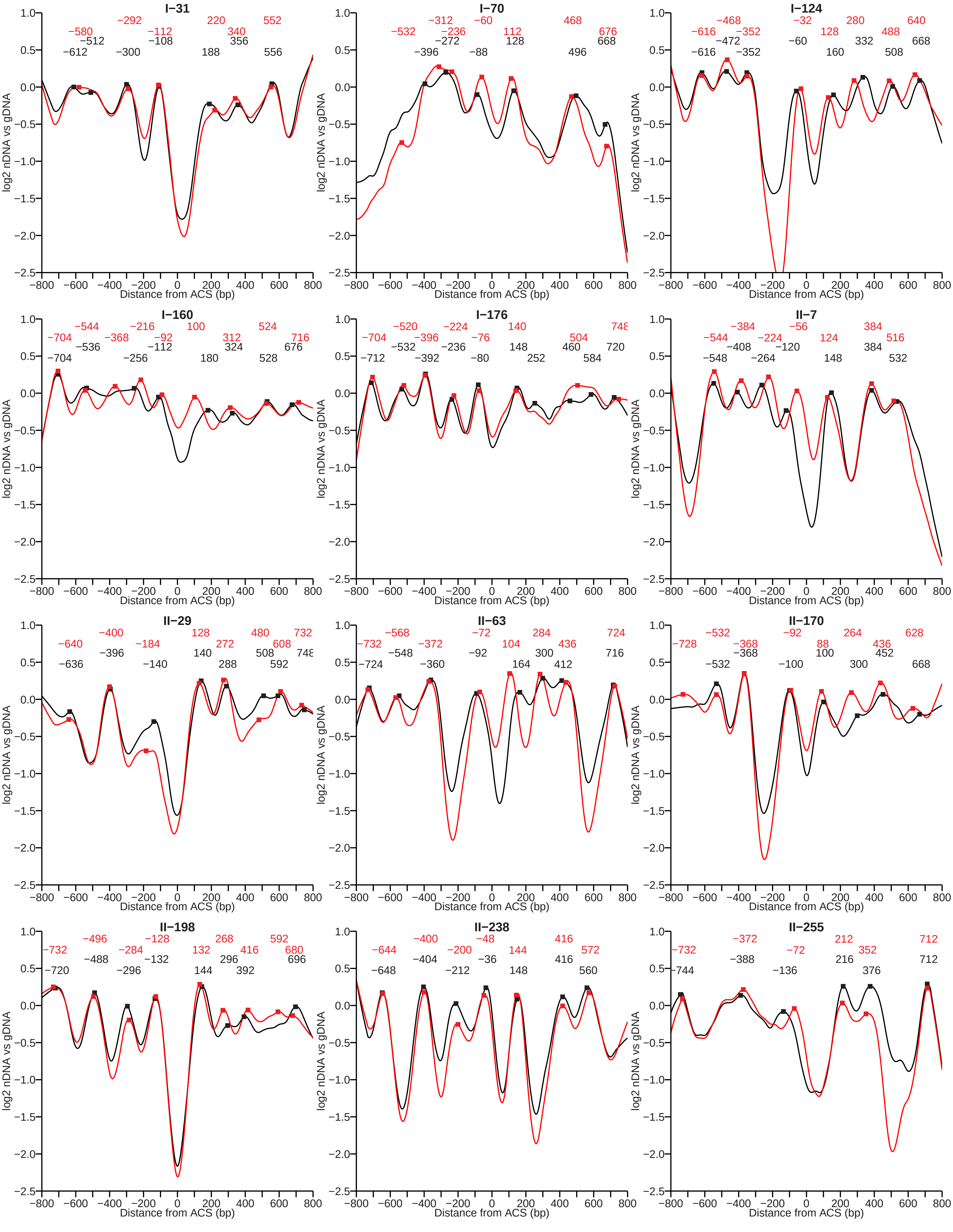
I−31
−292 220 552
−580 −112 340
−512 −108 356
−612 −300 188 556
I−70
−312 −60 468
−532 −236 112 676
−272 128 668
−396 −88 496
I−124
−468 −32 280 640
−616 −352 128 488
−472 −60 332 668
−616 −352 160 508
I−160
−544 −216 100 524
−704 −368 −92 312 716
−536 −112 324 676
−704 −256 180 528
I−176
−520 −224 140 748
−704 −396 −76 504
−532 −236 148 460 720
−712 −392 −80 252 584
II−7
−384 −56 384
−544 −224 124 516
−408 −120 384
−548 −264 148 532
II−29
−400 128 480 732
−640 −184 272 608
−396 140 508 748
−636 −140 288 592
II−63
−568 −72 284 724
−732 −372 104 436
−548 −92 300 716
−724 −360 164 412
II−170
−532 −92 264 628
−728 −368 88 436
−368 100 452
−532 −100 300 668
II−198
−496 −128 268 592
−732 −284 132 416 680
−488 −132 296 696
−720 −296 144 392
II−238
−400 −48 416
−644 −200 144 572
−404 −36 416
−648 −212 148 560
II−255
−372 212 712
−732 −72 352
−388 216 712
−744 −136 376
log2 nDNA vs gDNA
Distance from ACS (bp)

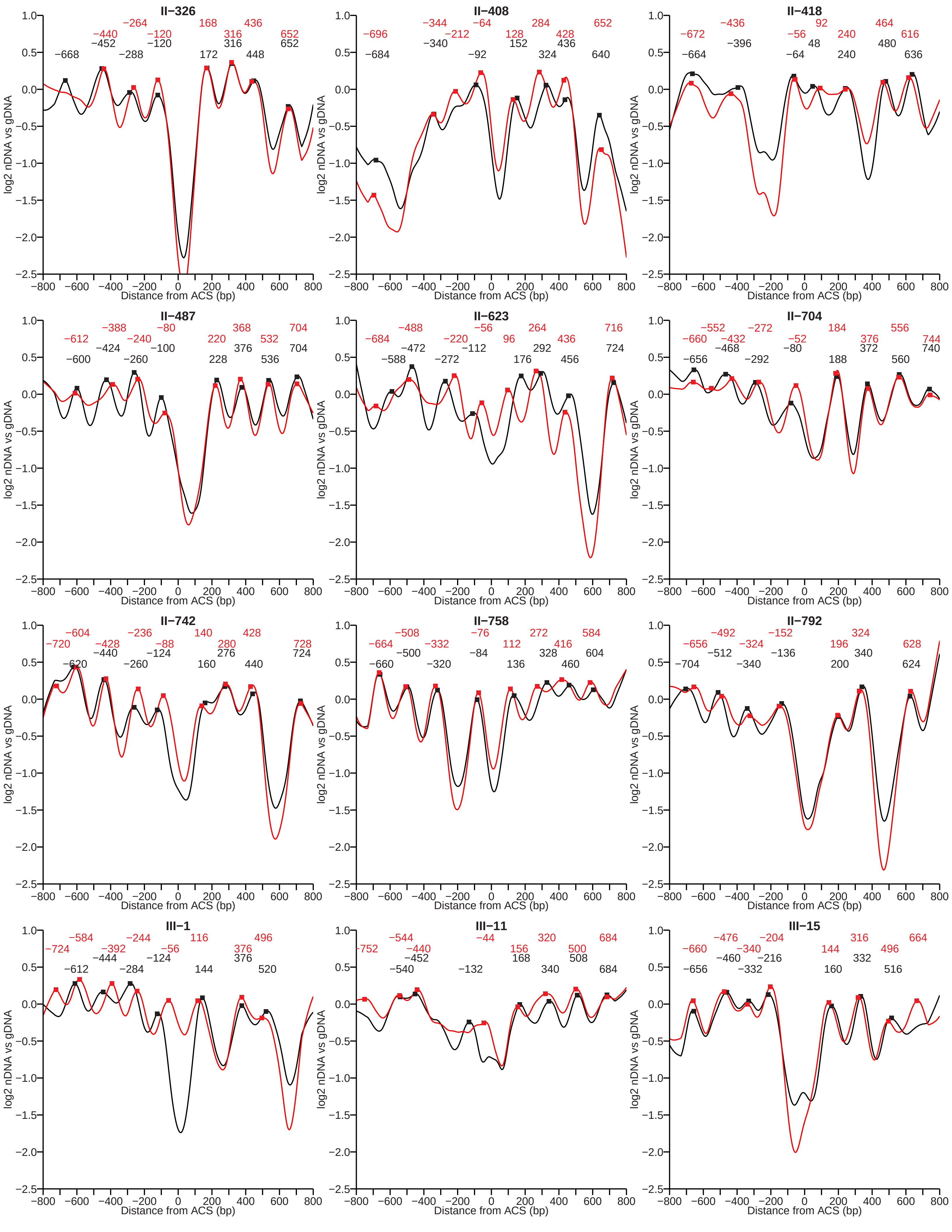

II−326
−264 168 436
−440 −120 316 652
−452 −120 316 652
−668 −288 172 448
II−408
−344 −64 284 652
−696 −212 128 428
−340 152 436
−684 −92 324 640
II−418
−436 92 464
−672 −56 240 616
−396 48 480
−664 −64 240 636
II−487
−388 −80 368 704
−612 −240 220 532
−424 −100 376 704
−600 −260 228 536
II−623
−488 −56 264 716
−684 −220 96 436
−472 −112 292 724
−588 −272 176 456
II−704
−552 −272 184 556
−660 −432 −52 376 744
−468 −80 372 740
−656 −292 188 560
II−742
−604 −236 140 428
−720 −428 −88 280 728
−440 −124 276 724
−620 −260 160 440
II−758
−508 −76 272 584
−664 −332 112 416
−500 −84 328 604
−660 −320 136 460
II−792
−492 −152 324
−656 −324 196 628
−512 −136 340
−704 −340 200 624
III−1
−584 −244 116 496
−724 −392 −56 376
−444 −124 376
−612 −284 144 520
III−11
−544 −44 320 684
−752 −440 156 500
−452 168 508
−540 −132 340 684
III−15
−476 −204 316 664
−660 −340 144 496
−460 −216 332
−656 −332 160 516
log2 nDNA vs gDNA
Distance from ACS (bp)

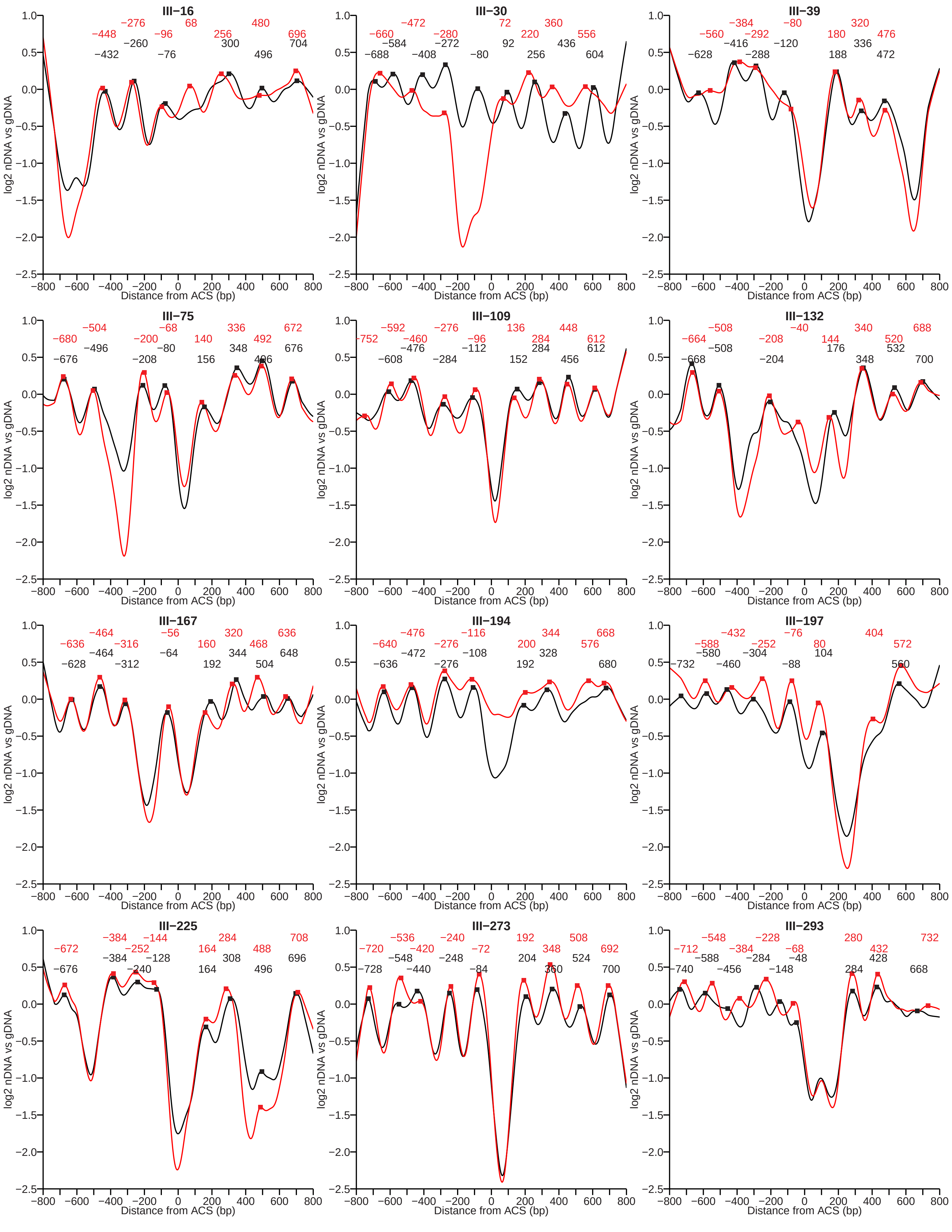
III−16
−448 −276 −96 68 256 480 696
−432 −260 −76 300 496 704
III−30
−660 −472 −280 72 220 360 556
−688 −584 −408 −272 −80 92 256 436 604
III−39
−560 −384 −292 −80 180 320 476
−628 −416 −288 −120 188 336 472
III−75
−680 −504 −200 −68 140 336 492 672
−676 −496 −208 −80 156 348 496 676
III−109
−752 −592 −460 −276 −96 136 284 448 612
−608 −476 −284 −112 152 284 456 612
III−132
−664 −508 −208 −40 144 340 520 688
−668 −508 −204 176 348 532 700
III−167
−636 −464 −316 −56 160 320 468 636
−628 −464 −312 −64 192 344 504 648
III−194
−640 −476 −276 −116 200 344 576 668
−636 −472 −276 −108 192 328 680
III−197
−588 −432 −252 −76 80 404 572
−732 −580 −460 −304 −88 104 560
III−225
−672 −384 −252 −144 164 284 488 708
−676 −384 −240 −128 164 308 496 696
III−273
−720 −536 −420 −240 −72 192 348 508 692
−728 −548 −440 −248 −84 204 360 524 700
III−293
−712 −548 −384 −228 −68 280 432 732
−740 −588 −456 −284 −148 −48 284 428 668
log2 nDNA vs gDNA
Distance from ACS (bp)
1.0 0.5 0.0 −0.5 −1.0 −1.5 −2.0 −2.5
−800 −600 −400 −200 0 200 400 600 800

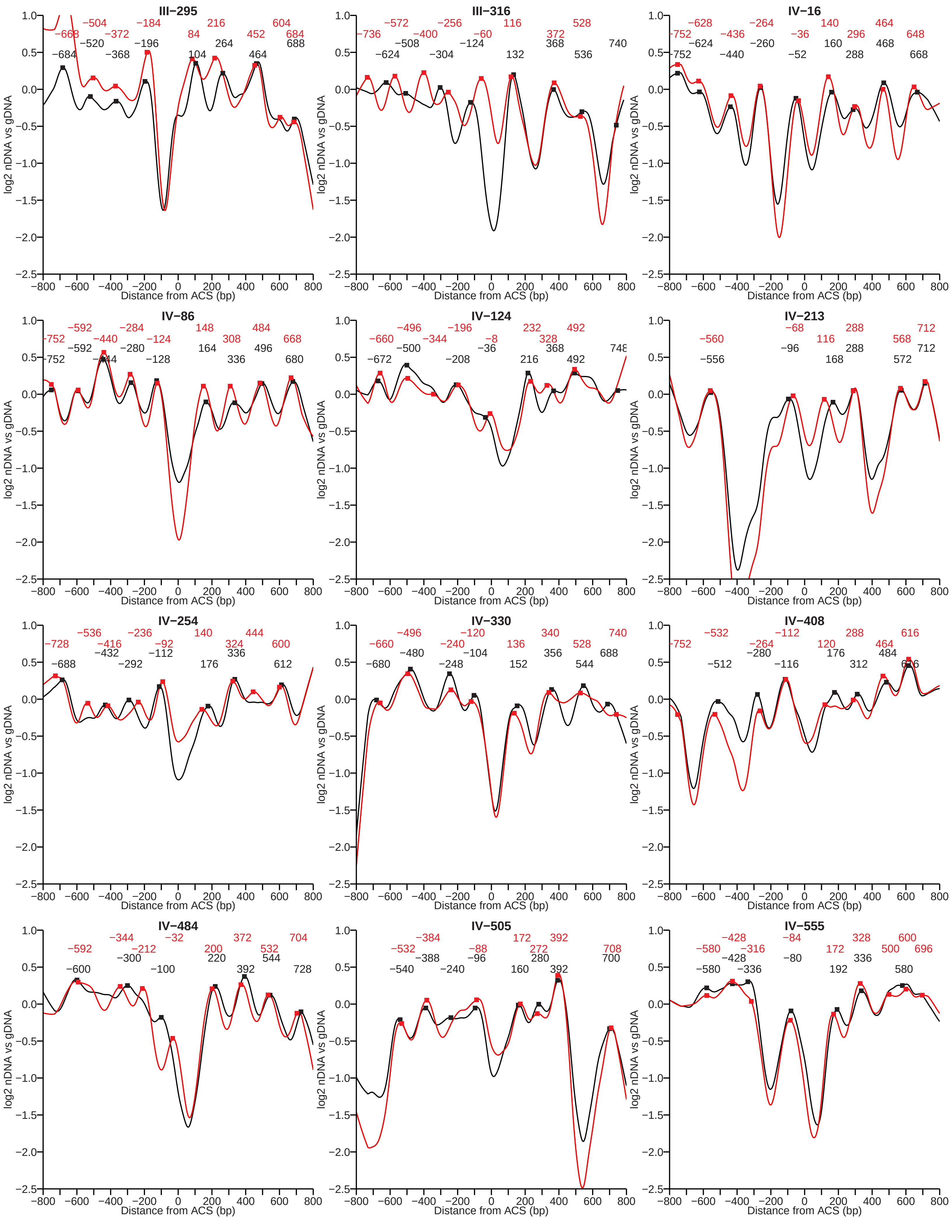
III−295
−504 −184 216 604
−668 −372 84 452 684
−520 −196 264 688
−684 −368 104 464
III−316
−572 −256 116 528
−736 −400 −60 372
−508 −124 368 740
−624 −304 132 536
IV−16
−628 −264 140 464
−752 −436 −36 296 648
−624 −260 160 468
−752 −440 −52 288 668
IV−86
−592 −284 148 484
−752 −440 −124 308 668
−592 −280 164 496
−752 −444 −128 336 680
IV−124
−496 −196 232 492
−660 −344 −8 328
−500 −36 368 748
−672 −208 216 492
IV−213
−68 288 712
−560 116 568
−96 288 712
−556 168 572
IV−254
−536 −236 140 444
−728 −416 −92 324 600
−432 −112 336
−688 −292 176 612
IV−330
−496 −120 340 740
−660 −240 136 528
−480 −104 356 688
−680 −248 152 544
IV−408
−532 −112 288 616
−752 −264 120 464
−280 176 484
−512 −116 312 616
IV−484
−344 −32 372 704
−592 −212 200 532
−300 220 544
−600 −100 392 728
IV−505
−384 172 392
−532 −88 272 708
−388 −96 280 700
−540 −240 160 392
IV−555
−428 −84 328 600
−580 −316 172 500 696
−428 −80 336
−580 −336 192 580
log2 nDNA vs gDNA
Distance from ACS (bp)

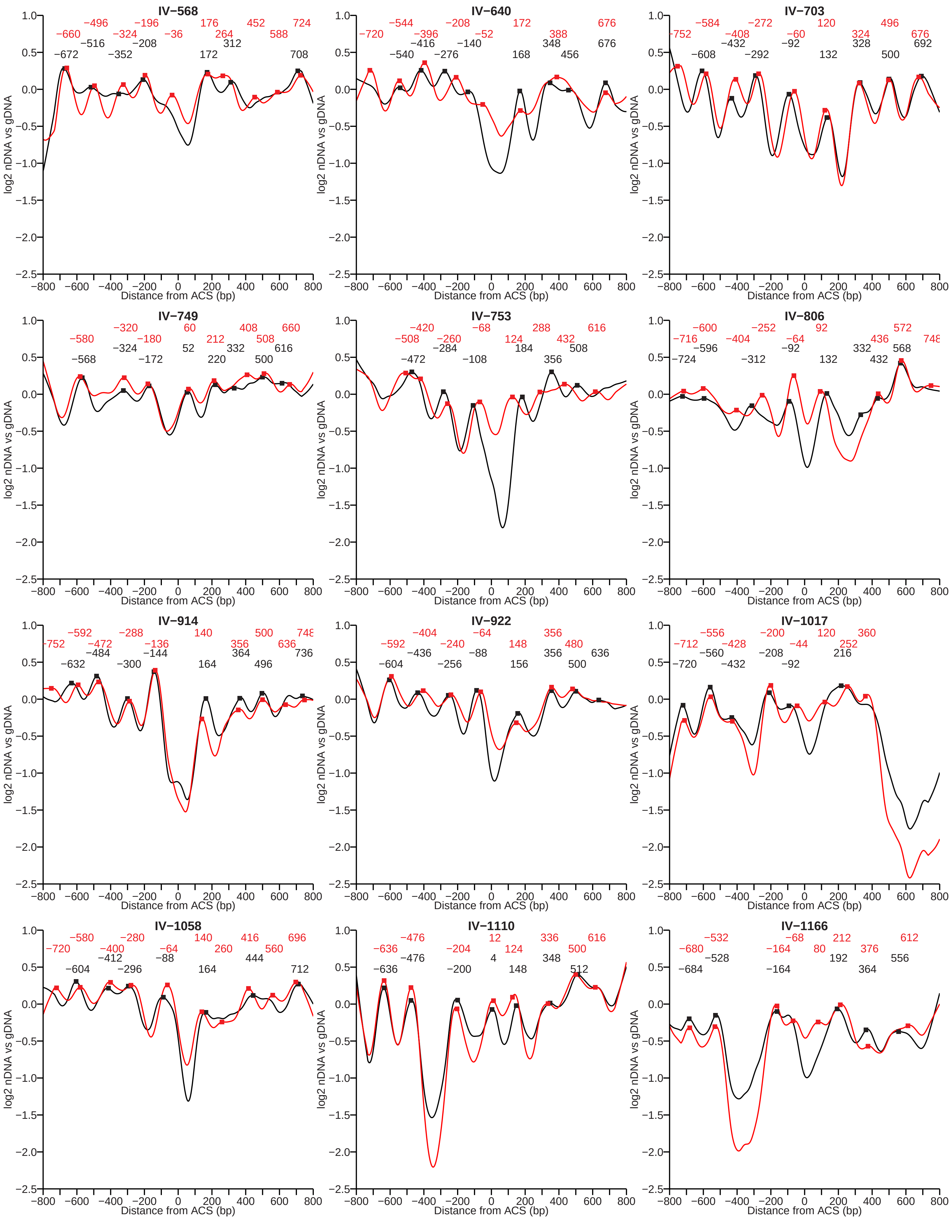
IV−568
−496 −196 176 452 724
−660 −324 −36 264 588
−516 −208 312
−672 −352 172 708
IV−640
−544 −208 172 676
−720 −396 −52 388
−416 −140 348 676
−540 −276 168 456
IV−703
−584 −272 120 496
−752 −408 −60 324 676
−432 −92 328 692
−608 −292 132 500
IV−749
−320 60 408 660
−580 −180 212 508
−324 52 332 616
−568 −172 220 500
IV−753
−420 −68 288 616
−508 −260 124 432
−284 184 508
−472 −108 356
IV−806
−600 −252 92 572
−716 −404 −64 436 748
−596 −92 332 568
−724 −312 132 432
IV−914
−592 −288 140 500 748
−752 −472 −136 356 636
−484 −144 364 736
−632 −300 164 496
IV−922
−404 −64 356
−592 −240 148 480
−436 −88 356 636
−604 −256 156 500
IV−1017
−556 −200 120 360
−712 −428 −44 252
−560 −208 216
−720 −432 −92
IV−1058
−580 −280 140 416 696
−720 −400 −64 260 560
−412 −88 444
−604 −296 164 712
IV−1110
−476 12 336 616
−636 −204 124 500
−476 4 348
−636 −200 148 512
IV−1166
−532 −68 212 612
−680 −164 80 376
−528 192 556
−684 −164 364
log2 nDNA vs gDNA
1.0 0.5 0.0 −0.5 −1.0 −1.5 −2.0 −2.5
Distance from ACS (bp)
−800 −600 −400 −200 0 200 400 600 800

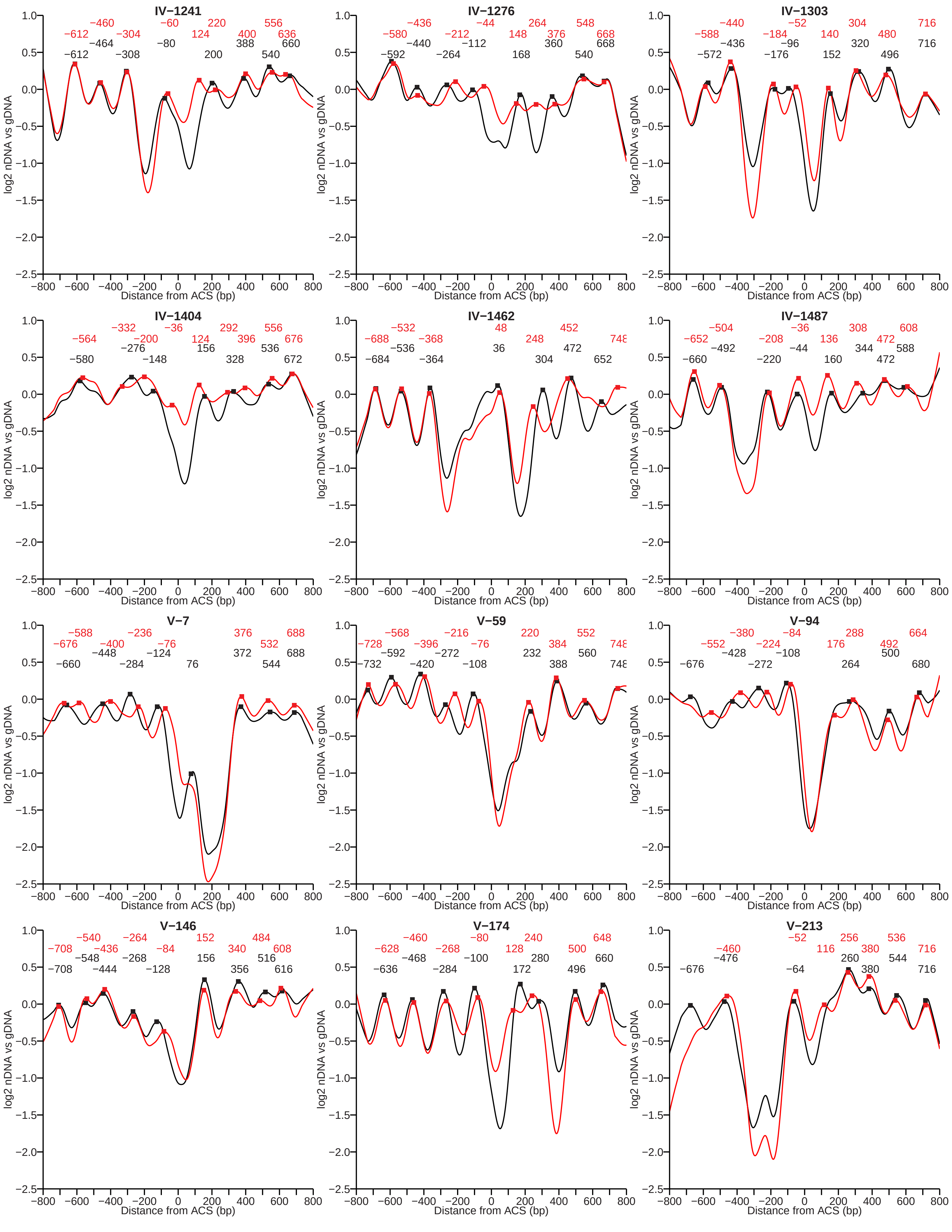

IV−1241
−460 −612 −304 −60 220 124 400 556 636
−464 −80 388 660 −612 −308 200 540
IV−1276
−436 −580 −212 −44 264 148 376 548 668
−440 −112 360 668 −592 −264 168 540
IV−1303
−440 −588 −184 −52 140 304 480 716
−436 −96 320 716 −572 −176 152 496
IV−1404
−332 −564 −36 −200 124 292 396 556 676
−276 156 536 −580 −148 328 672
IV−1462
−532 −688 −368 48 248 452 748
−536 36 472 −684 −364 304 652
IV−1487
−504 −652 −36 −208 136 308 472 608
−492 −44 344 588 −660 −220 160 472
V−7
−588 −676 −400 −236 −76 376 532 688
−448 −124 372 688 −660 −284 76 544
V−59
−568 −728 −396 −216 −76 220 384 552 748
−592 −272 232 560 −732 −420 −108 388 748
V−94
−380 −552 −224 −84 288 176 492 664
−428 −108 500 −676 −272 264 680
V−146
−540 −708 −436 −264 −84 152 340 484 608
−548 −268 156 516 −708 −444 −128 356 616
V−174
−460 −628 −268 −80 128 240 500 648
−468 −100 280 660 −636 −284 172 496
V−213
−460 −52 116 256 380 536 716
−476 260 544 −676 −64 380 716
log2 nDNA vs gDNA
Distance from ACS (bp)

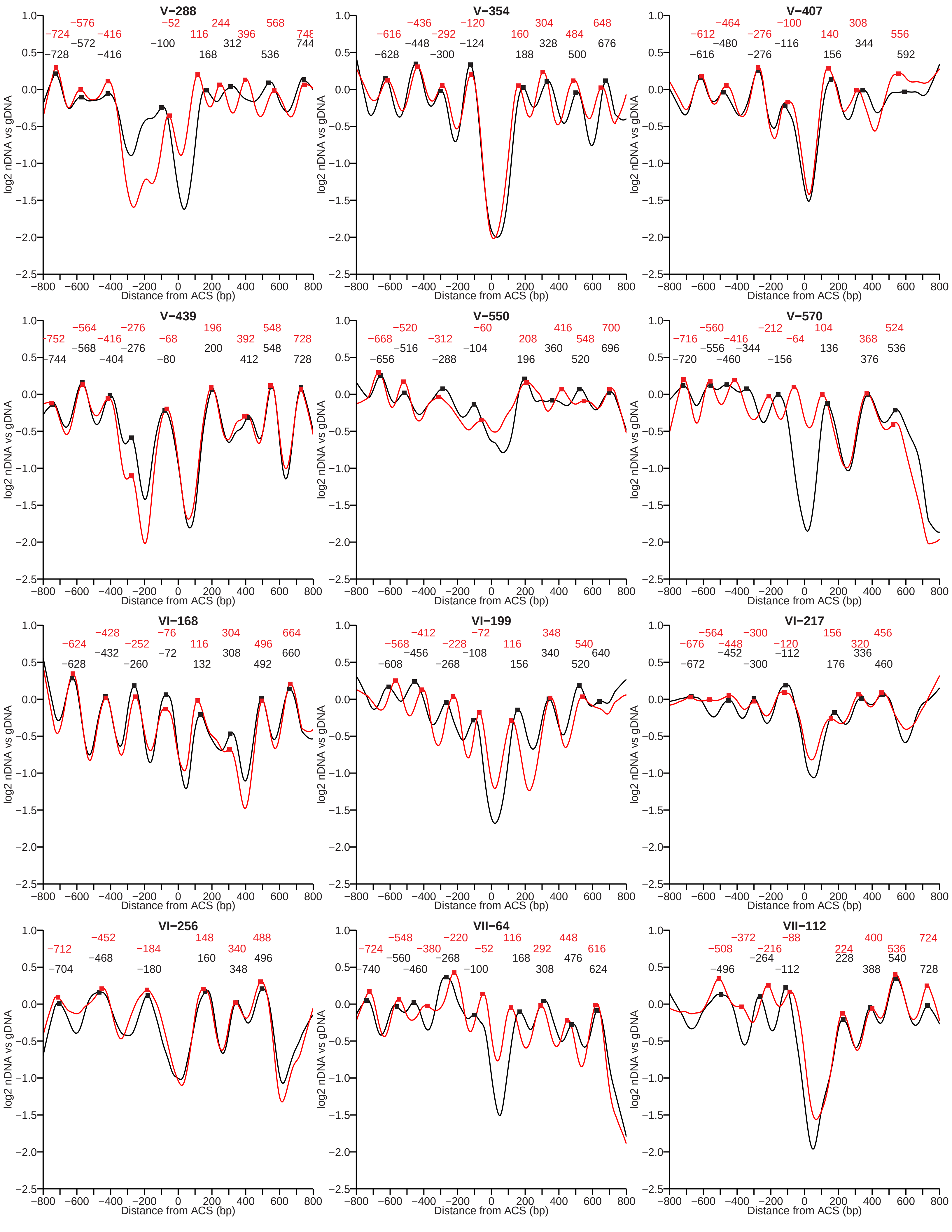

V−288
−576 −52 244 568
−724 −416 116 396 748
−572 −100 312 744
−728 −416 168 536
V−354
−436 −120 304 648
−616 −292 160 484
−448 −124 328 676
−628 −300 188 500
V−407
−464 −100 308
−612 −276 140 556
−480 −116 344
−616 −276 156 592
V−439
−564 −276 196 548
−752 −416 −68 392 728
−568 −276 200 548
−744 −404 −80 412 728
V−550
−520 −60 416 700
−668 −312 208 548
−516 −104 360 696
−656 −288 196 520
V−570
−560 −212 104 524
−716 −416 −64 368
−556 −344 136 536
−720 −460 −156 376
VI−168
−428 −76 304 664
−624 −252 116 496
−432 −72 308 660
−628 −260 132 492
VI−199
−412 −72 348
−568 −228 116 540
−456 −108 340 640
−608 −268 156 520
VI−217
−564 −300 156 456
−676 −448 −120 320
−452 −112 336
−672 −300 176 460
VI−256
−452 148 488
−712 −184 340
−468 160 496
−704 −180 348
VII−64
−548 −220 116 448
−724 −380 −52 292 616
−560 −268 168 476
−740 −460 −100 308 624
VII−112
−372 −88 400 724
−508 −216 224 536
−264 228 540
−496 −112 388 728
log2 nDNA vs gDNA
Distance from ACS (bp)

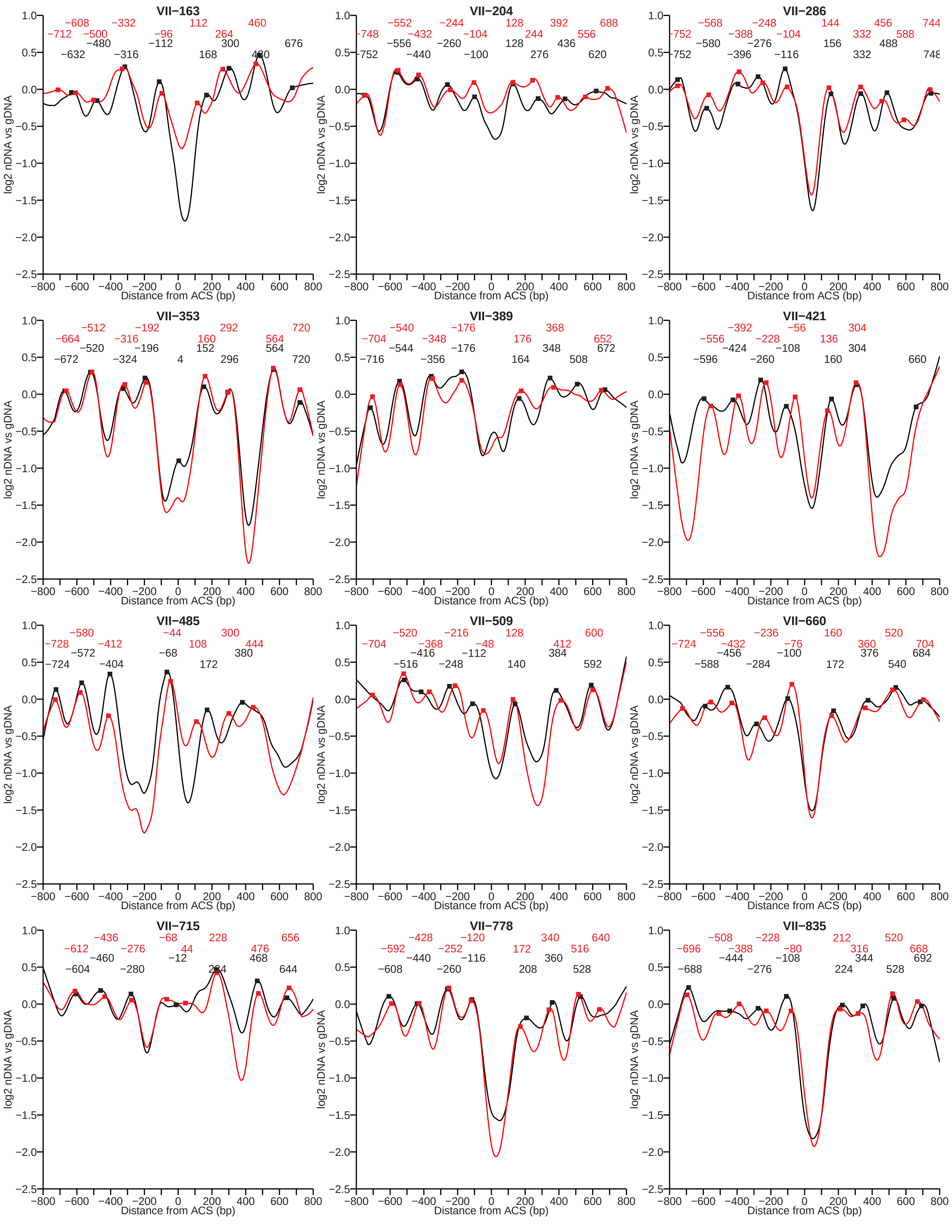

VII−163
−608 −332 112 460
−712 −500 −96 264
−480 −112 300 676
−632 −316 168 480
VII−204
−552 −244 128 392 688
−748 −432 −104 244 556
−556 −260 128 436
−752 −440 −100 276 620
VII−286
−568 −248 144 456 744
−752 −388 −104 332 588
−580 −276 156 488
−752 −396 −116 332 748
VII−353
−512 −192 292 720
−664 −316 160 564
−520 −196 152 564
−672 −324 4 296 720
VII−389
−540 −176 368
−704 −348 176 652
−544 −176 348 672
−716 −356 164 508
VII−421
−392 −56 304
−556 −228 136
−424 −108 304
−596 −260 160 660
VII−485
−580 −44 300
−728 −412 108 444
−572 −68 380
−724 −404 172
VII−509
−520 −216 128 600
−704 −368 −48 412
−416 −112 384
−516 −248 140 592
VII−660
−556 −236 160 520
−724 −432 −76 360 704
−456 −100 376 684
−588 −284 172 540
VII−715
−436 −68 228 656
−612 −276 44 476
−460 −12 468
−604 −280 224 644
VII−778
−428 −120 340 640
−592 −252 172 516
−440 −116 360
−608 −260 208 528
VII−835
−508 −228 212 520
−696 −388 −80 316 668
−444 −108 344 692
−688 −276 224 528
log2 nDNA vs gDNA
Distance from ACS (bp)

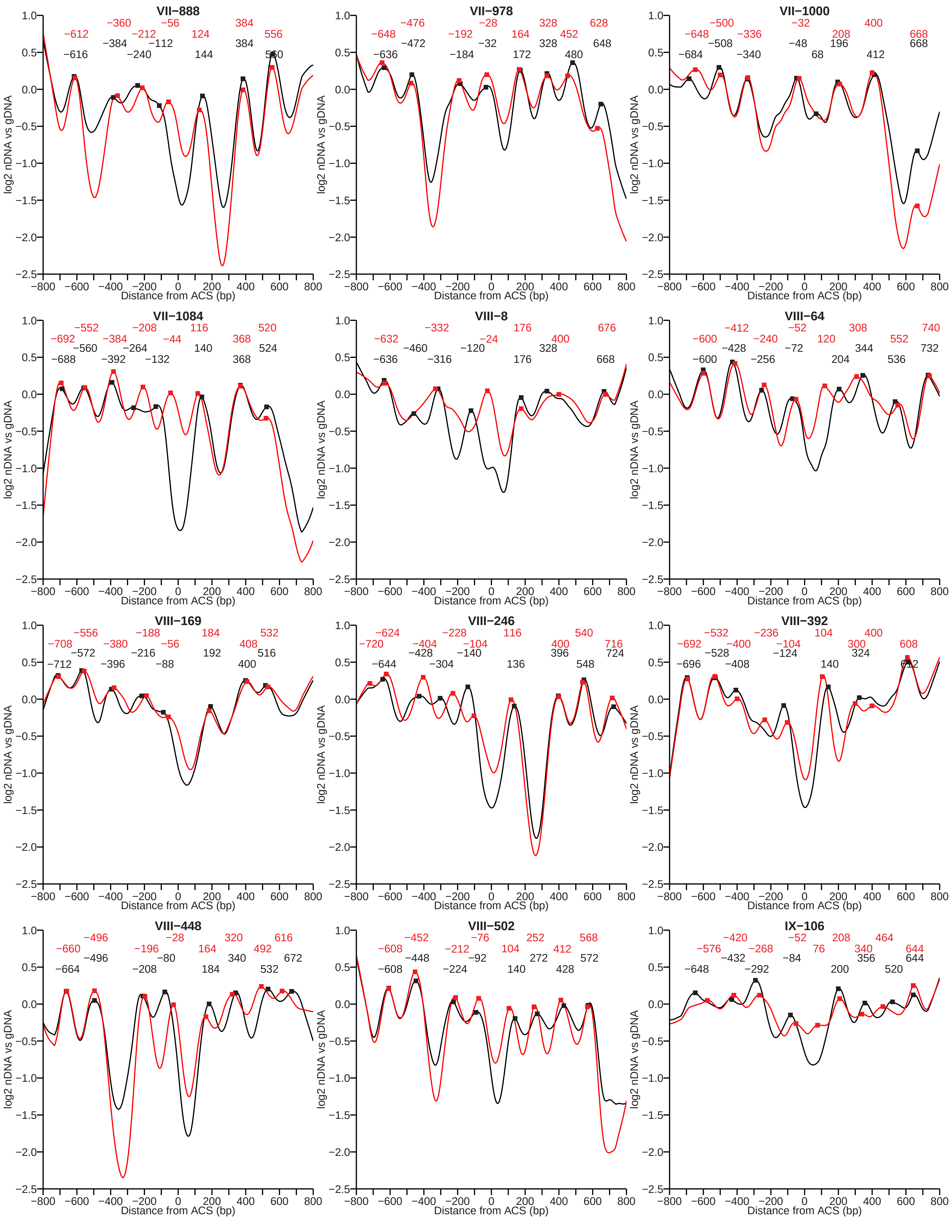

VII−888
−612 −360 −212 −56 124 384 556
−616 −384 −240 −112 144 384 560
VII−978
−648 −476 −192 −28 164 328 452 628
−636 −472 −184 −32 172 328 480 648
VII−1000
−648 −500 −336 −32 208 400 668
−684 −508 −340 −48 68 196 412 668
VII−1084
−692 −552 −384 −208 −44 116 368 520
−688 −560 −392 −264 −132 140 368 524
VIII−8
−632 −332 −24 176 400 676
−636 −460 −316 −120 176 328 668
VIII−64
−600 −412 −240 −52 120 308 552 740
−600 −428 −256 −72 204 344 536 732
VIII−169
−708 −556 −380 −188 −56 184 408 532
−712 −572 −396 −216 −88 192 400 516
VIII−246
−720 −624 −404 −228 −104 116 400 540 716
−644 −428 −304 −140 136 396 548 724
VIII−392
−692 −532 −400 −236 −104 104 300 400 608
−696 −528 −408 −124 140 324 612
VIII−448
−660 −496 −196 −28 164 320 492 616
−664 −496 −208 −80 184 340 532 672
VIII−502
−608 −452 −212 −76 104 252 412 568
−608 −448 −224 −92 140 272 428 572
IX−106
−576 −420 −268 −52 76 208 340 464 644
−648 −432 −292 −84 200 356 520 644
log2 nDNA vs gDNA
Distance from ACS (bp)

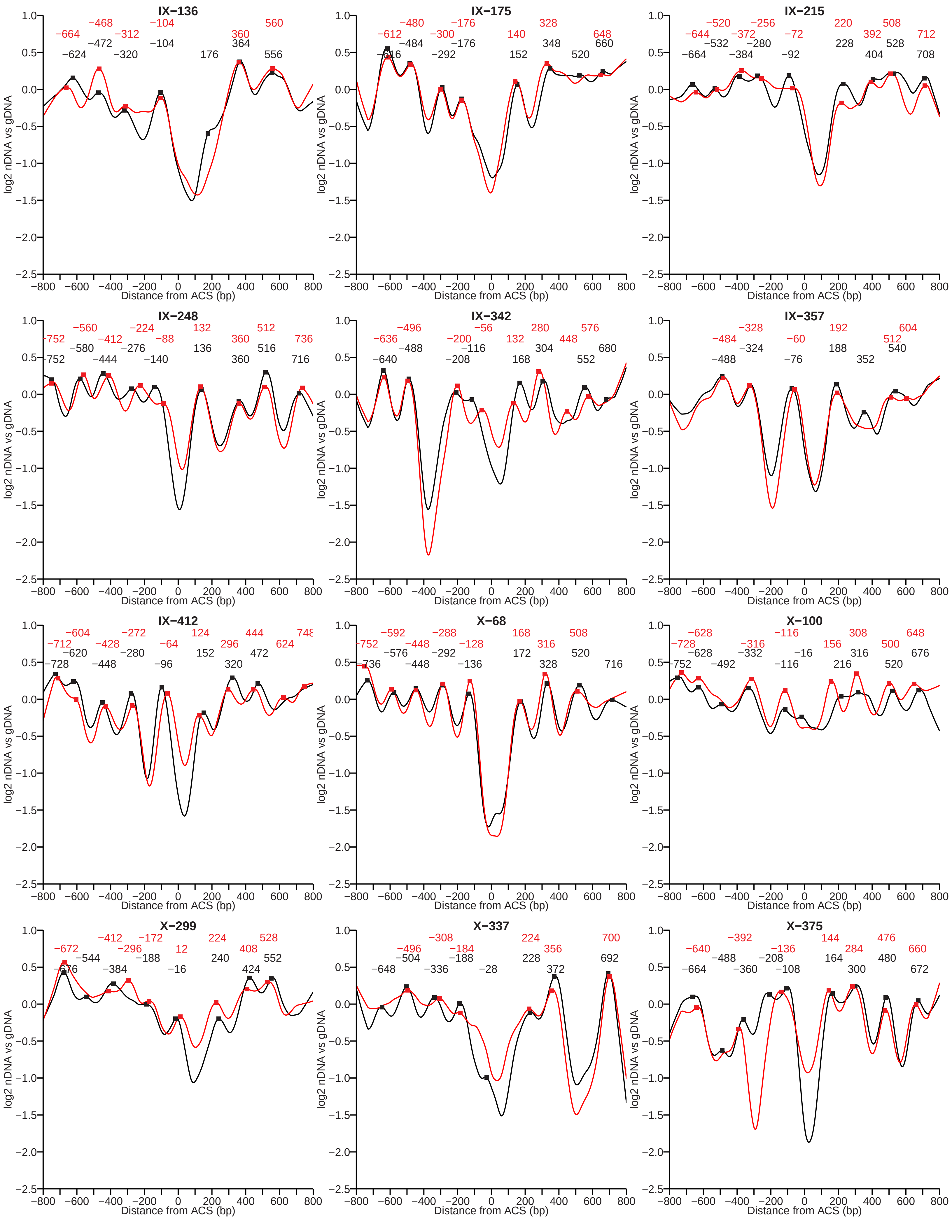

IX−136
−664 −468 −312 −104 360 560
−624 −472 −320 −104 176 364 556
IX−175
−612 −480 −300 −176 140 328 648
−616 −484 −292 −176 152 348 520 660
IX−215
−644 −520 −372 −256 −72 220 392 508 712
−664 −532 −384 −280 −92 228 404 528 708
IX−248
−752 −560 −412 −224 −88 132 360 512 736
−752 −580 −444 −276 −140 136 360 516 716
IX−342
−636 −496 −200 −56 132 280 448 576
−640 −488 −208 −116 168 304 552 680
IX−357
−484 −328 −60 192 512 604
−488 −324 −76 188 352 540
IX−412
−712 −604 −428 −272 −64 124 296 444 624 748
−728 −620 −448 −280 −96 152 320 472
X−68
−752 −592 −448 −288 −128 168 316 508
−736 −576 −448 −292 −136 172 328 520 716
X−100
−728 −628 −316 −116 156 308 500 648
−752 −628 −492 −332 −116 −16 216 316 520 676
X−299
−672 −412 −296 −172 12 224 408 528
−676 −544 −384 −188 −16 240 424 552
X−337
−496 −308 −184 224 356 700
−648 −504 −336 −188 −28 228 372 692
X−375
−640 −392 −136 144 284 476 660
−664 −488 −360 −208 −108 164 300 480 672
log2 nDNA vs gDNA
Distance from ACS (bp)

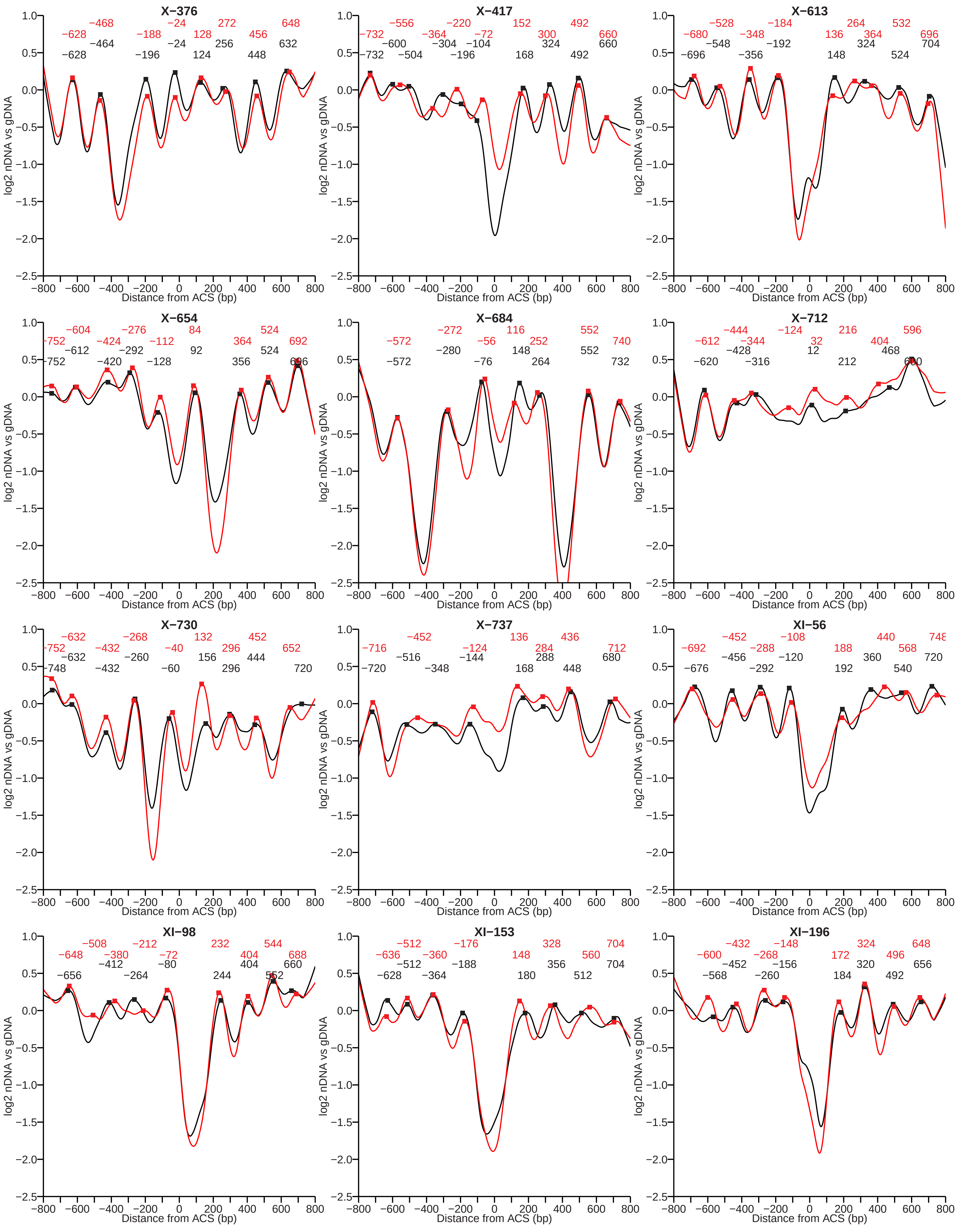
X−376
−468 −24 272 648
−628 −188 128 456
−464 −24 256 632
−628 −196 124 448
X−417
−556 −220 152 492
−732 −364 −72 300 660
−600 −304 −104 324 660
−732 −504 −196 168 492
X−613
−528 −184 264 532
−680 −348 136 364 696
−548 −192 324 704
−696 −356 148 524
X−654
−604 −276 84 524
−752 −424 −112 364 692
−612 −292 92 524
−752 −420 −128 356 696
X−684
−272 116 552
−572 −56 252 740
−280 148 552
−572 −76 264 732
X−712
−444 −124 216 596
−612 −344 32 404
−428 12 468
−620 −316 212 600
X−730
−632 −268 132 452
−752 −432 −40 296 652
−632 −260 156 444
−748 −432 −60 296 720
X−737
−452 136 436
−716 −124 284 712
−516 −144 288 680
−720 −348 168 448
XI−56
−452 −108 440 748
−692 −288 188 568
−456 −120 360 720
−676 −292 192 540
XI−98
−508 −212 232 544
−648 −380 −72 404 688
−412 −80 404 660
−656 −264 244 552
XI−153
−512 −176 328 704
−636 −360 148 560
−512 −188 356 704
−628 −364 180 512
XI−196
−432 −148 324 648
−600 −268 172 496
−452 −156 320 656
−568 −260 184 492
log2 nDNA vs gDNA
Distance from ACS (bp)
1.0 0.5 0.0 −0.5 −1.0 −1.5 −2.0 −2.5
−800 −600 −400 −200 0 200 400 600 800

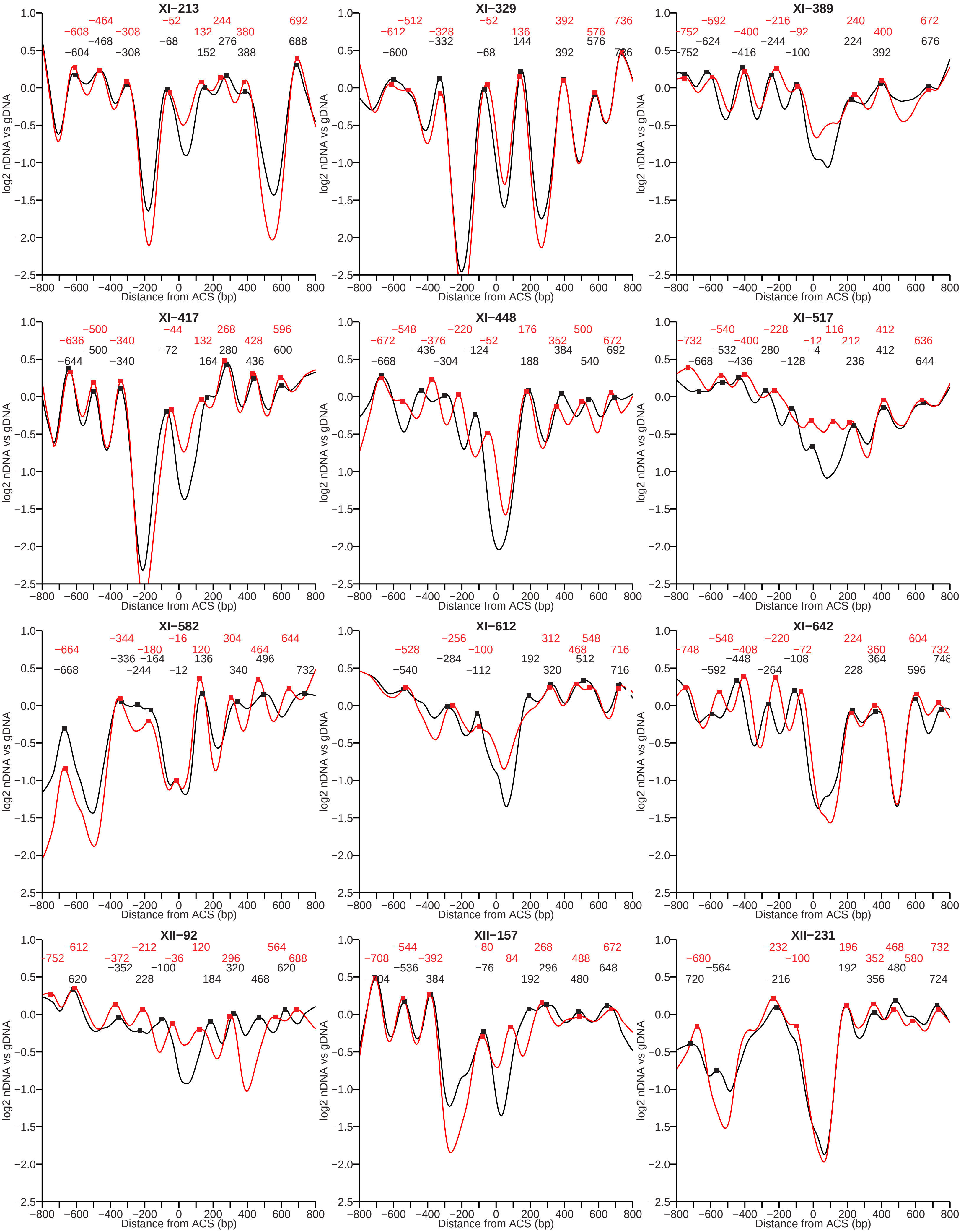

XI–213
−464 −52 244 692
−608 −308 132 380
−468 −68 276 688
−604 −308 152 388
XI–329
−512 −52 392 736
−612 −328 136 576
−332 144 576
−600 −68 392 736
XI–389
−592 −216 240 672
−752 −400 −92 400
−624 −244 224 676
−752 −416 −100 392
XI–417
−500 −44 268 596
−636 −340 132 428
−500 −72 280 600
−644 −340 164 436
XI–448
−548 −220 176 500
−672 −376 −52 352 672
−436 −124 384 692
−668 −304 188 540
XI–517
−540 −228 116 412
−732 −400 −12 212 636
−532 −280 −4 412
−668 −436 −128 236 644
XI–582
−344 −16 304 644
−664 −180 120 464
−336 −164 136 496
−668 −244 −12 340 732
XI–612
−256 312 548
−528 −100 468 716
−284 192 512
−540 −112 320 716
XI–642
−548 −220 224 604
−748 −408 −72 360 732
−448 −108 364 748
−592 −264 228 596
XII–92
−612 −212 120 564
−752 −372 −36 296 688
−352 −100 320 620
−620 −228 184 468
XII–157
−544 −80 268 672
−708 −392 84 488
−536 −76 296 648
−704 −384 192 480
XII–231
−232 196 468 732
−680 −100 352 580
−564 192 480
−720 −216 356 724
log2 nDNA vs gDNA
Distance from ACS (bp)
1.0
0.5
0.0
−0.5
−1.0
−1.5
−2.0
−2.5
−800 −600 −400 −200 0 200 400 600 800

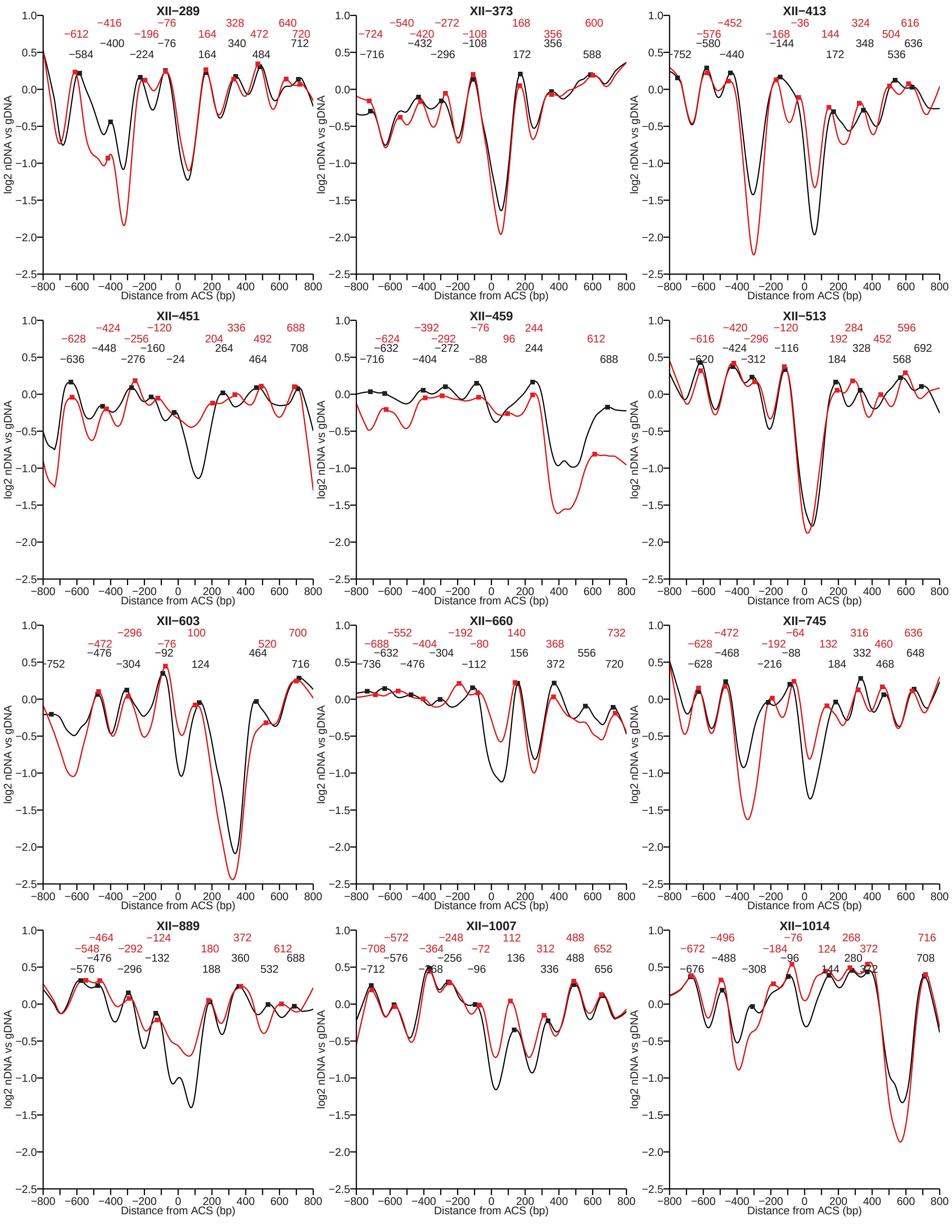
XII−289
−416 −76 328 640
−612 −196 164 472 720
−400 −76 340 712
−584 −224 164 484
XII−373
−540 −272 168 600
−724 −420 −108 356
−432 −108 356
−716 −296 172 588
XII−413
−452 −36 324 616
−576 −168 144 504
−580 −144 348 636
−752 −440 172 536
XII−451
−424 −120 336 688
−628 −256 204 492
−448 −160 264 708
−636 −276 −24 464
XII−459
−392 −76 244
−624 −292 96 612
−632 −272 244
−716 −404 −88 688
XII−513
−420 −120 284 596
−616 −296 192 452
−424 −116 328 692
−620 −312 184 568
XII−603
−296 100 700
−472 −76 520
−476 −92 464
−752 −304 124 716
XII−660
−552 −192 140 732
−688 −404 −80 368
−632 −304 156 556
−736 −476 −112 372 720
XII−745
−472 −64 316 636
−628 −192 132 460
−468 −88 332 648
−628 −216 184 468
XII−889
−464 −124 372
−548 −292 180 612
−476 −132 360 688
−576 −296 188 532
XII−1007
−572 −248 112 488
−708 −364 −72 312 652
−576 −256 136 488
−712 −368 −96 336 656
XII−1014
−496 −76 268 716
−672 −184 124 372
−488 −96 280 708
−676 −308 144 372
log2 nDNA vs gDNA
Distance from ACS (bp)

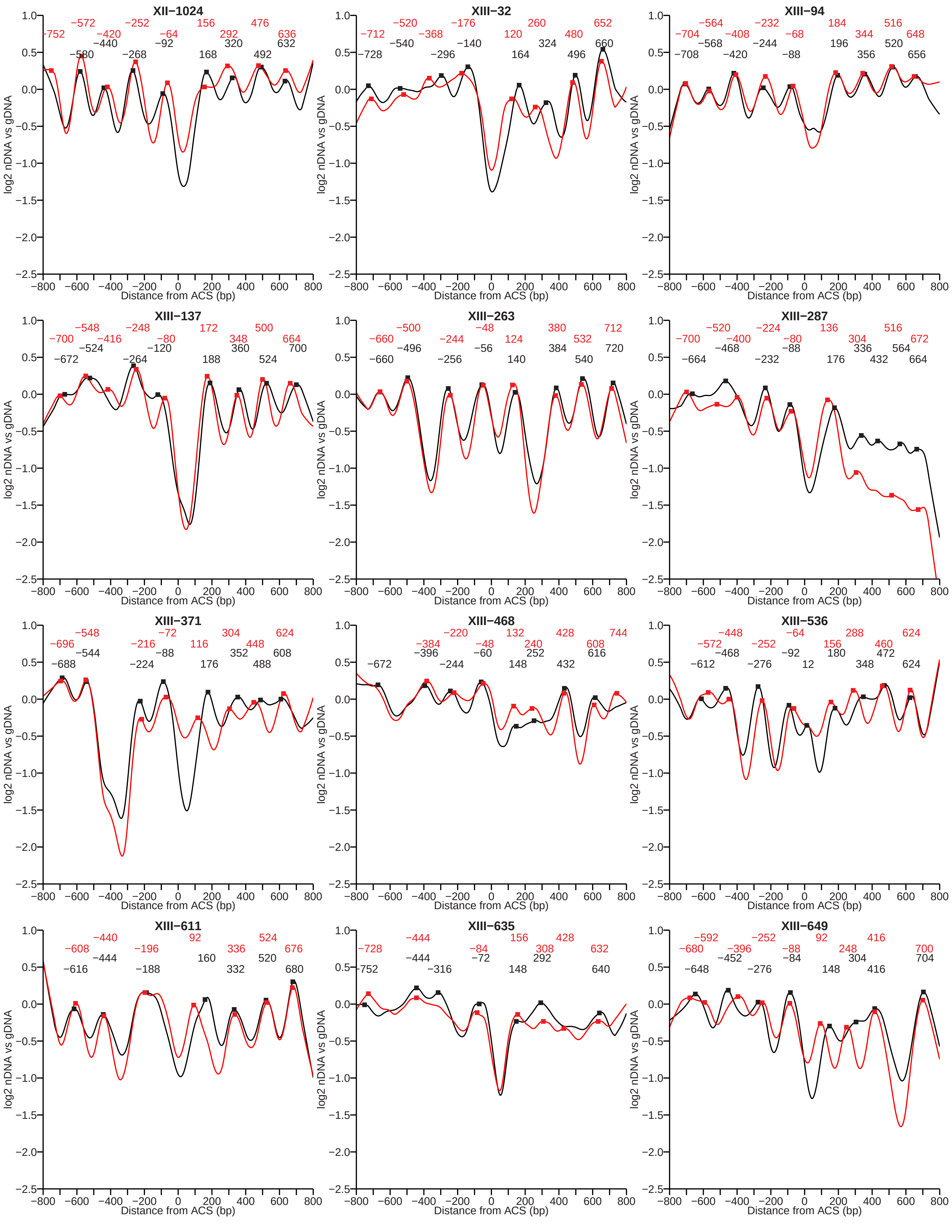
XII−1024
−752 −572 −420 −252 −64 156 292 476 636
−580 −440 −268 −92 168 320 492 632
XIII−32
−712 −520 −368 −176 120 260 480 652
−728 −540 −296 −140 164 324 496 660
XIII−94
−704 −564 −408 −232 −68 184 344 516 648
−708 −568 −420 −244 −88 196 356 520 656
XIII−137
−700 −548 −416 −248 −80 172 348 500 664
−672 −524 −264 −120 188 360 524 700
XIII−263
−660 −500 −244 −48 124 380 532 712
−660 −496 −256 −56 140 384 540 720
XIII−287
−700 −520 −400 −224 −80 136 304 516 672
−664 −468 −232 −88 176 336 432 564 664
XIII−371
−696 −548 −216 −72 116 304 448 624
−688 −544 −224 −88 176 352 488 608
XIII−468
−384 −220 −48 132 240 428 608 744
−672 −396 −244 −60 148 252 432 616
XIII−536
−572 −448 −252 −64 156 288 460 624
−612 −468 −276 −92 12 180 348 472 624
XIII−611
−608 −440 −196 92 336 524 676
−616 −444 −188 160 332 520 680
XIII−635
−728 −444 −84 156 308 428 632
−752 −444 −316 −72 148 292 640
XIII−649
−680 −592 −396 −252 −88 92 248 416 700
−648 −452 −276 −84 148 304 416 704
log2 nDNA vs gDNA
Distance from ACS (bp)
1.0 0.5 0.0 −0.5 −1.0 −1.5 −2.0 −2.5
−800 −600 −400 −200 0 200 400 600 800

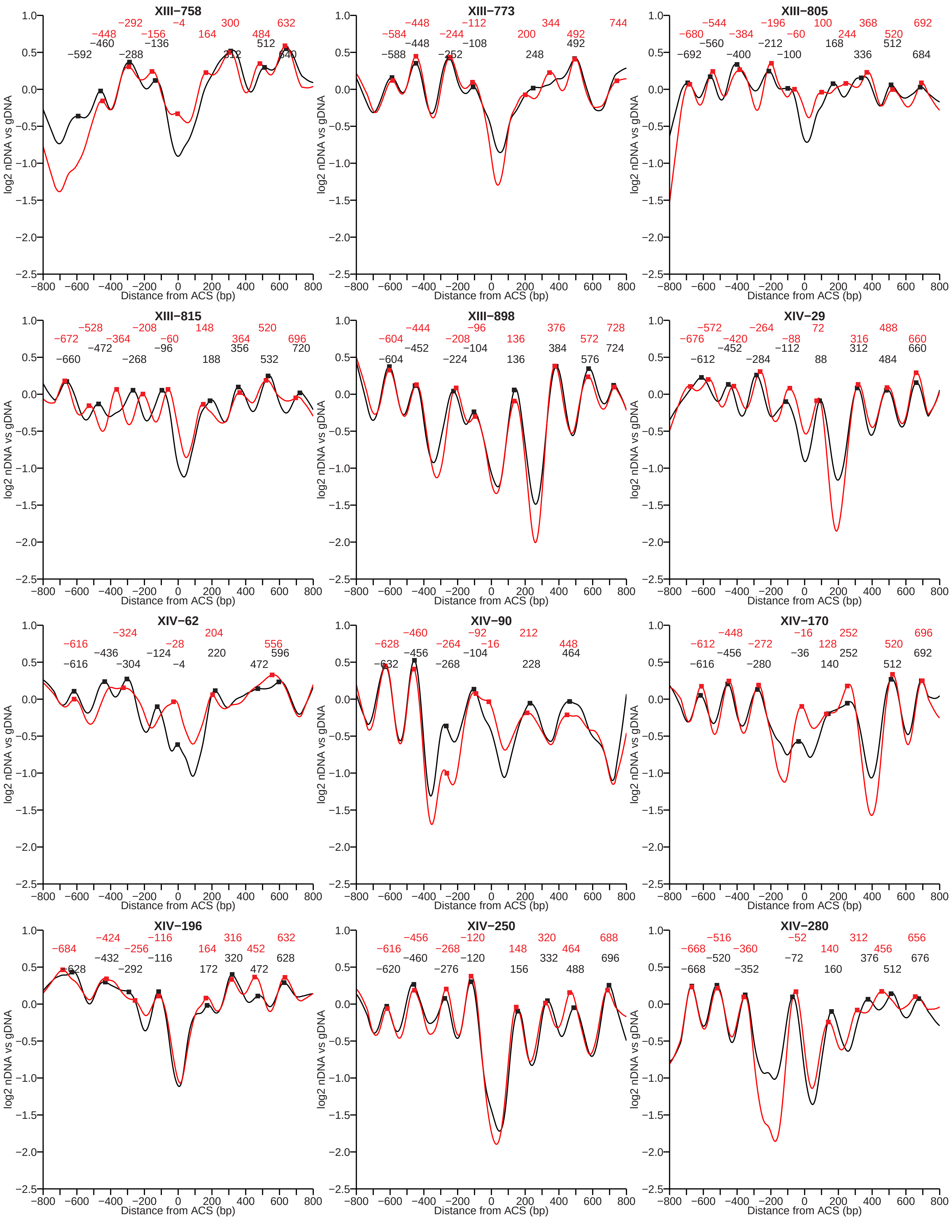
XIII−758
−292 −4 300 632
−448 −156 164 484
−460 −136 512
−592 −288 312 640
XIII−773
−448 −112 344 744
−584 −244 200 492
−448 −108 492
−588 −252 248
XIII−805
−544 −196 100 368 692
−680 −384 −60 244 520
−560 −212 168 512
−692 −400 −100 336 684
XIII−815
−528 −208 148 520
−672 −364 −60 364 696
−472 −96 356 720
−660 −268 188 532
XIII−898
−444 −96 376 728
−604 −208 136 572
−452 −104 384 724
−604 −224 136 576
XIV−29
−572 −264 72 488
−676 −420 −88 316 660
−452 −112 312 660
−612 −284 88 484
XIV−62
−324 204
−616 −28 556
−436 −124 220 596
−616 −304 −4 472
XIV−90
−460 −92 212
−628 −264 −16 448
−456 −104 464
−632 −268 228
XIV−170
−448 −16 252 696
−612 −272 128 520
−456 −36 252 692
−616 −280 140 512
XIV−196
−424 −116 316 632
−684 −256 164 452
−432 −116 320 628
−628 −292 172 472
XIV−250
−456 −120 320 688
−616 −268 148 464
−460 −120 332 696
−620 −276 156 488
XIV−280
−516 −52 312 656
−668 −360 140 456
−520 −72 376 676
−668 −352 160 512
log2 nDNA vs gDNA
Distance from ACS (bp)
1.0 0.5 0.0 −0.5 −1.0 −1.5 −2.0 −2.5
−800 −600 −400 −200 0 200 400 600 800

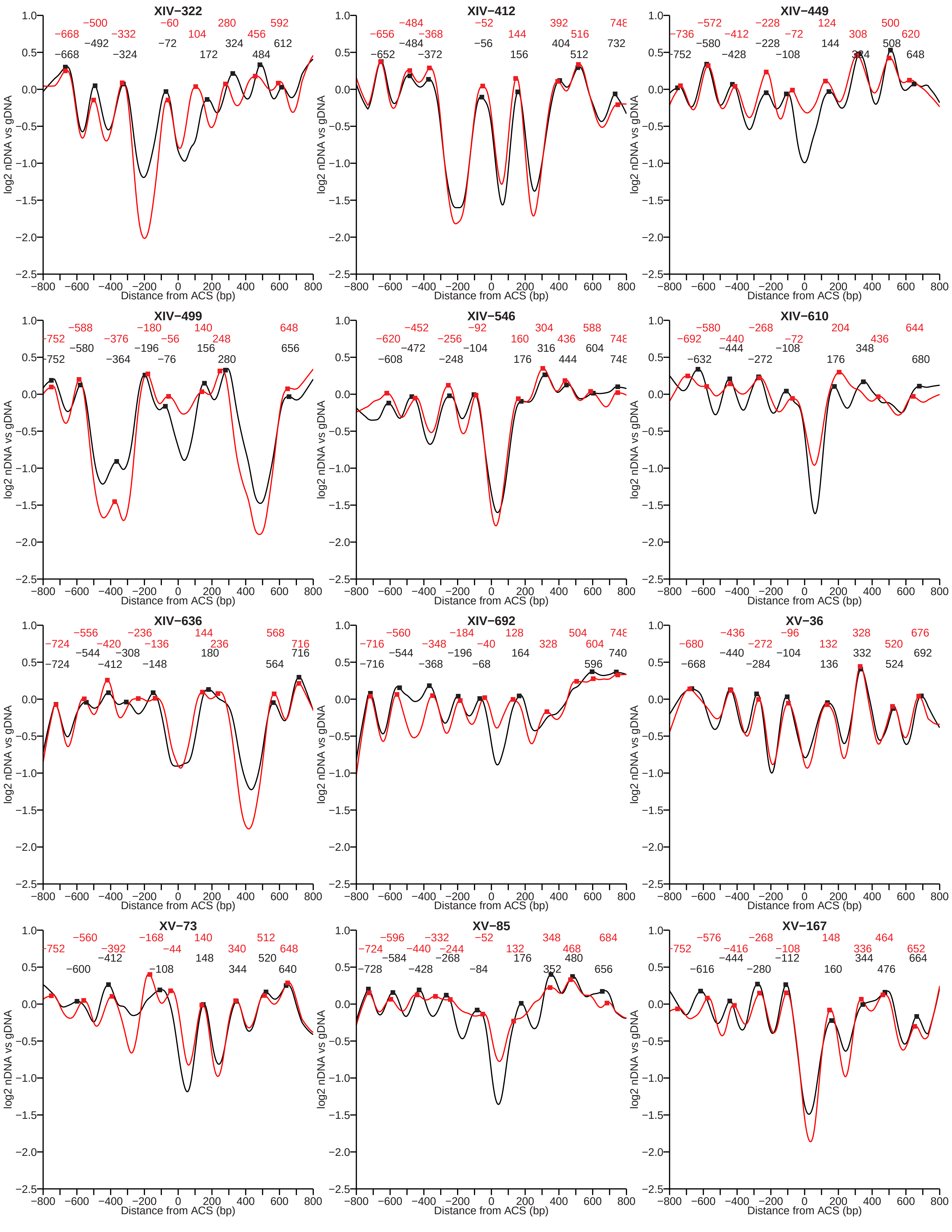

XIV−322
−500 −60 280 592
−668 −332 104 456
−492 −72 324 612
−668 −324 172 484
XIV−412
−484 −52 392 748
−656 −368 144 516
−484 −56 404 732
−652 −372 156 512
XIV−449
−572 −228 124 500
−736 −412 −72 308 620
−580 −228 144 508
752 −428 −108 324 648
XIV−499
−588 −180 140 648
−752 −376 −56 248
−580 −196 156 656
−752 −364 −76 280
XIV−546
−452 −92 304 588
−620 −256 160 436 748
−472 −104 316 604
−608 −248 176 444 748
XIV−610
−580 −268 204 644
−692 −440 −72 436
−444 −108 348
−632 −272 176 680
XIV−636
−556 −236 144 568
−724 −420 −136 236 716
−544 −308 180 716
−724 −412 −148 564
XIV−692
−560 −184 128 504 748
−716 −348 −40 328 604
−544 −196 164 740
−716 −368 −68 596
XV−36
−436 −96 328 676
−680 −272 132 520
−440 −104 332 692
−668 −284 136 524
XV−73
−560 −168 140 512
−752 −392 −44 340 648
−412 148 520
−600 −108 344 640
XV−85
−596 −332 −52 348 684
−724 −440 −244 132 468
−584 −268 176 480
−728 −428 −84 352 656
XV−167
−576 −268 148 464
−752 −416 −108 336 652
−444 −112 344 664
−616 −280 160 476
log2 nDNA vs gDNA
Distance from ACS (bp)

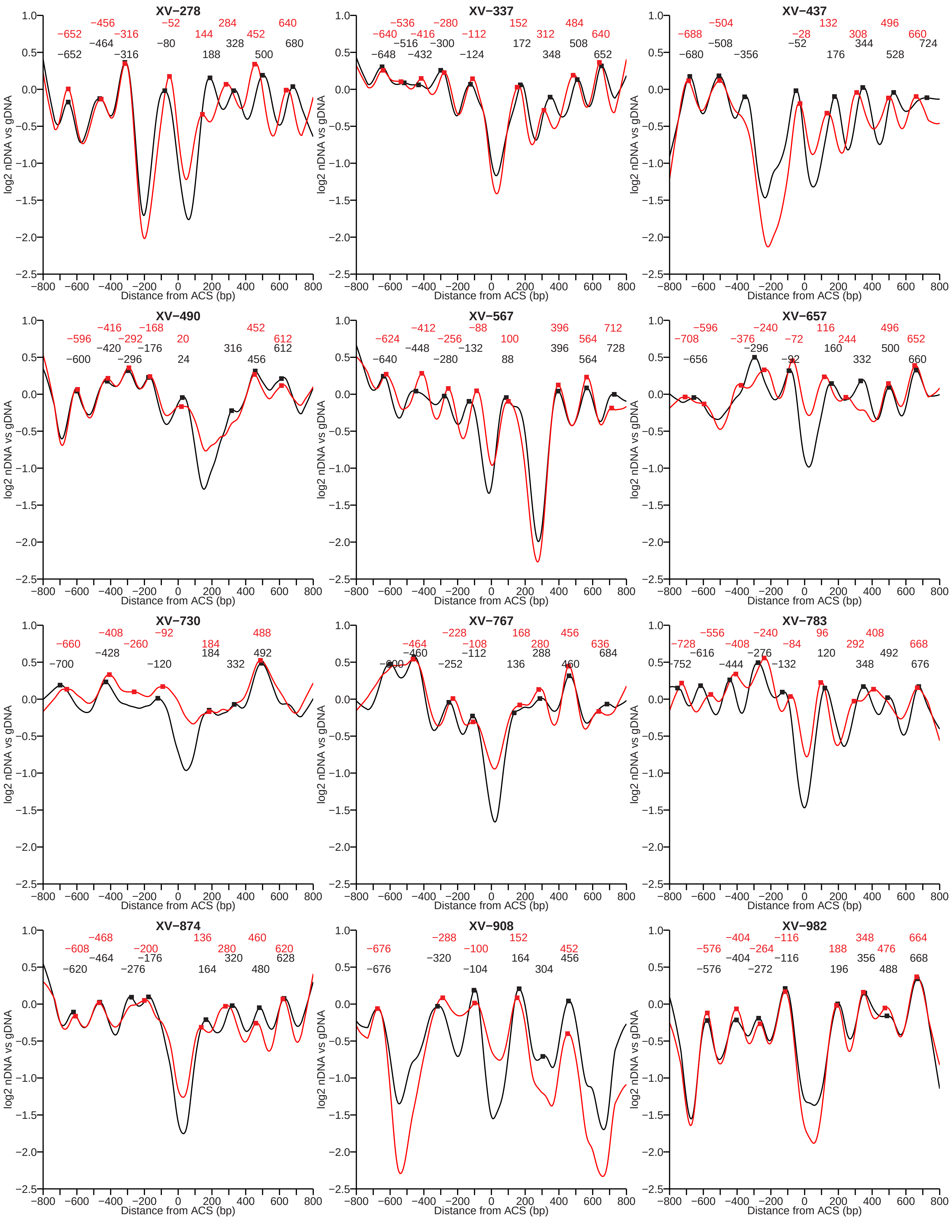
XV−278
XV−337
XV−437
XV−490
XV−567
XV−657
XV−730
XV−767
XV−783
XV−874
XV−908
XV−982
log2 nDNA vs gDNA
Distance from ACS (bp)

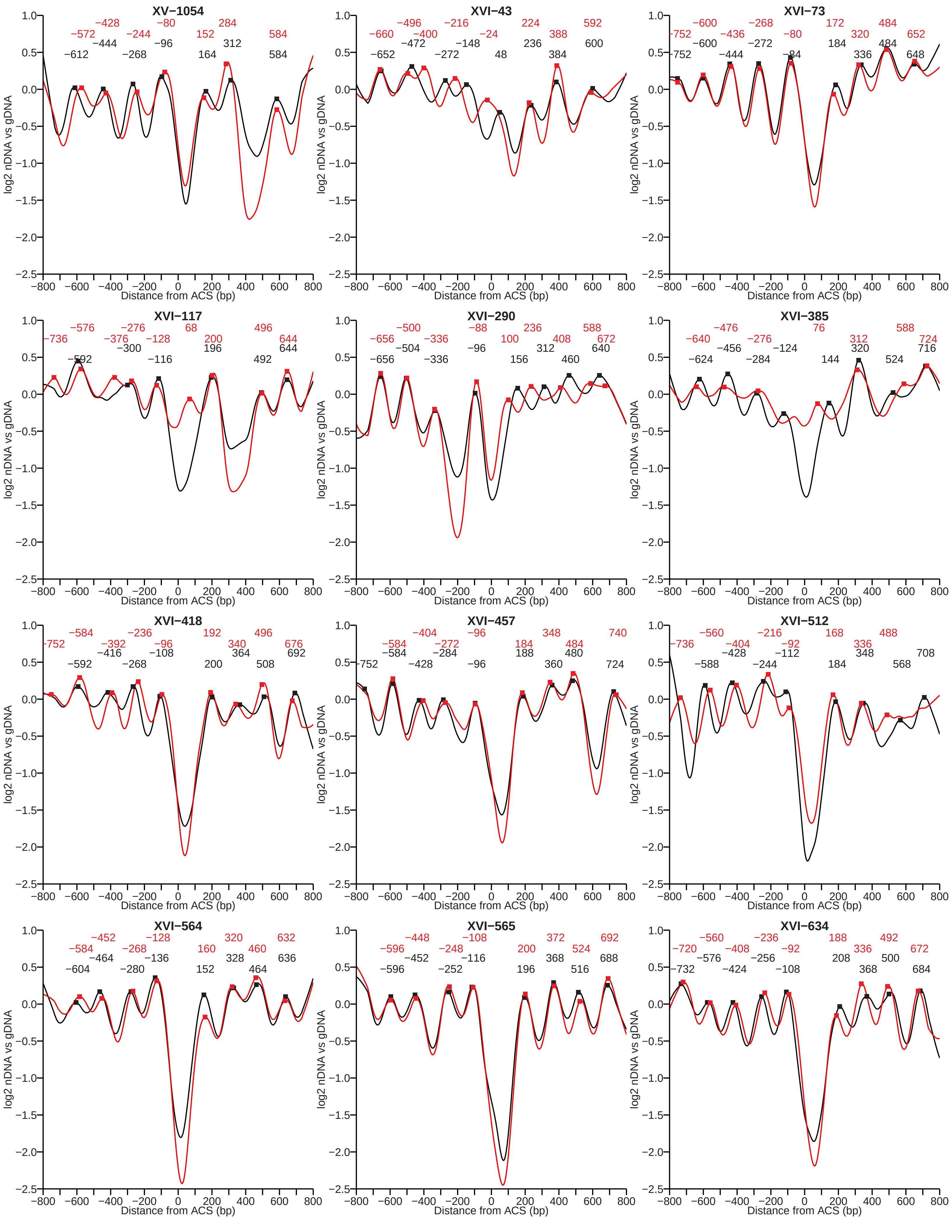
XV–1054
−428 −572 −244 −80 152 284 584
−444 −612 −268 −96 312 164 584
XVI–43
−496 −660 −400 −216 −24 224 388 592
−472 −652 −148 −272 48 236 600 384
XVI–73
−752 −600 −436 −268 −80 172 320 484 652
−600 −752 −444 −272 −84 184 336 484 648
XVI–117
−576 −736 −376 −276 −128 68 200 496 644
−592 −300 −116 196 492 644
XVI–290
−656 −500 −336 −88 100 236 408 588 672
−504 −656 −336 −96 312 156 460 640
XVI–385
−640 −476 −276 76 312 588 724
−624 −456 −284 −124 144 320 524 716
XVI–418
−752 −584 −392 −236 −96 192 340 496 676
−592 −416 −268 −108 200 364 508 692
XVI–457
−584 −404 −272 −96 184 348 484 740
−752 −584 −428 −284 −96 188 360 480 724
XVI–512
−736 −560 −404 −216 −92 168 336 488
−588 −428 −244 −112 184 348 568 708
XVI–564
−584 −452 −268 −128 160 320 460 632
−604 −464 −280 −136 152 328 464 636
XVI–565
−596 −448 −248 −108 200 372 524 692
−596 −452 −252 −116 196 368 516 688
XVI–634
−720 −560 −408 −236 −92 188 336 492 672
−732 −576 −424 −256 −108 208 368 500 684
log2 nDNA vs gDNA
Distance from ACS (bp)

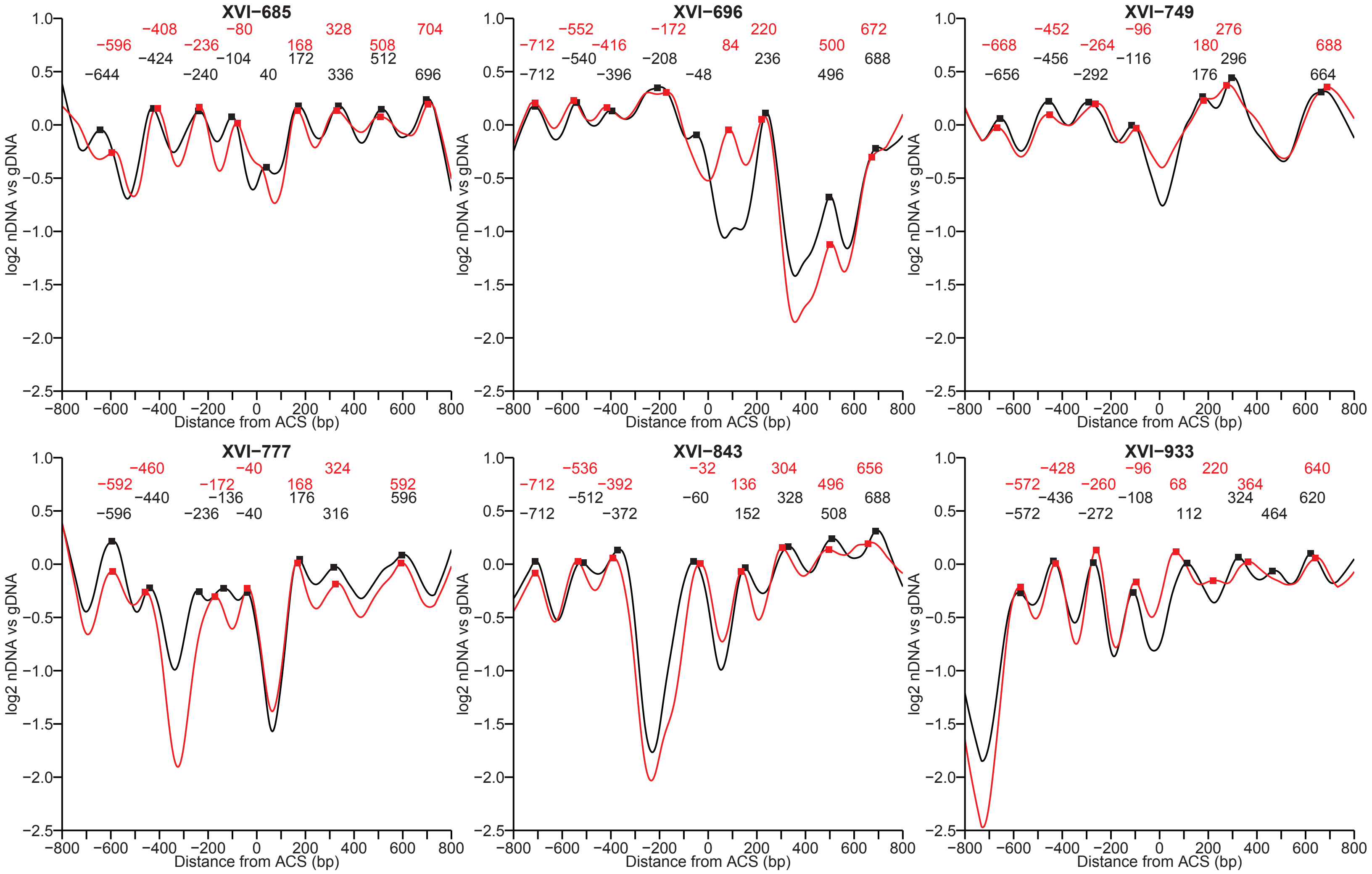

Figure S6. ACS-centered nucleosome profiles for each origin in *GAL:orc2-1* (red line) and the wild-type control (black line) datasets. Individual origins were LOESS smoothed using a span which included 35 probes. The nucleosome calls for each origin in *GAL:orc2-1* are indicated (red square) and their nucleosome locations are indicated with red text. The nucleosome calls for each origin in the wild-type control dataset (W303-1A) are indicated (black square) and their nucleosome locations are indiciated with black text. Origin names are based on the naming conventions of OriDB [9].
