# Supplementary material for: Diversity of Eukaryotic DNA Replication Origins Revealed by Genome-Wide Analysis of Chromatin Structure
Source: PLoS Genet. 2010 Sep 2;6(9):e1001092. doi: 10.1371/journal.pgen.1001092 (PMC2932696; doi:10.1371/journal.pgen.1001092)
Supplement: Figure S7 — The effect of ORC depletion on nucleosome occupancy at TSS elements. (0.23 MB PDF) [file pgen.1001092.s007.pdf]

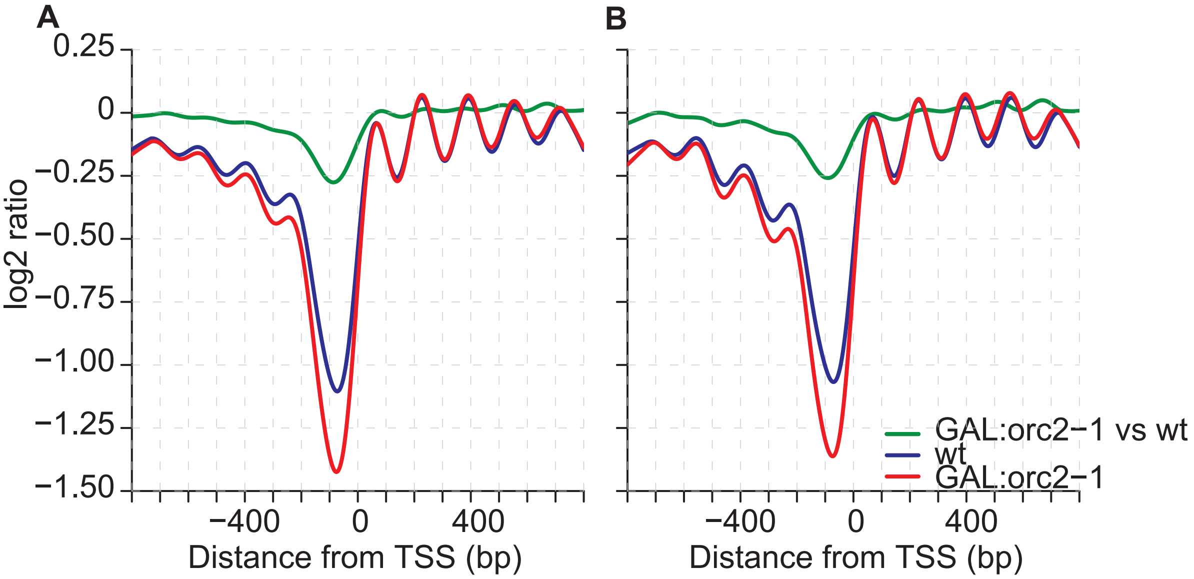

Figure S7. Average TSS centered profiles of the Orc2 depletion strain (—), the wild-type control strain (—) and the difference between the Orc2 depletion strain and the wild-type control strain (—). A. The average TSS profile for the complete dataset of 5015 genes. B. The average TSS profile for a random subset of 222 TSS profiles.
